# OSCAR functions as a collagen I receptor to suppress hippo signaling and reprogram lipid metabolism in clear-cell renal cell carcinoma

**DOI:** 10.1038/s41419-026-08713-1

**Published:** 2026-04-08

**Authors:** Hengyu Shi, Jian Shi, Xuejiao Dong, Songming Wu, Qingyang Lv, Qiangqiang Huang, Daojia Miao, Feiyi Lu, Chengtao Wang, Xiaoping Zhang, Huageng Liang

**Affiliations:** 1 Department of Urology, Union Hospital, Tongji Medical College, Huazhong University of Science and Technology, Wuhan, China; 2 Department of Pathogen Biology, School of Basic Medicine, Tongji Medical College, Huazhong University of Science and Technology, Wuhan, China

**Keywords:** Renal cell carcinoma, Cancer metabolism, Oncogenes

## Abstract

Extensive extracellular matrix (ECM) remodeling is a hallmark of clear-cell renal cell carcinoma (ccRCC), and collagen I has been widely implicated in ccRCC progression through multiple oncogenic pathways. However, the receptor-level mechanisms by which collagen I engages specific signaling and metabolic programs remain incompletely understood. Here, we demonstrate that collagen I is highly expressed in ccRCC and predicts poor prognosis. We further uncover OSCAR as its key functional receptor, mediating tumor progression and metabolic reprogramming through Hippo signaling modulation. Mechanistically, collagen I binding induces OSCAR internalization and its interaction with the Hippo regulator SAV1. This disrupts SAV1 membrane localization, allowing YAP to enter the nucleus and activate downstream genes, which enhances proliferation, metastasis, and de novo fatty acid synthesis. Furthermore, we designed a lipid nanoparticle (CCP-LNP) that blocks the collagen I–OSCAR interaction and effectively suppresses tumor progression in vitro and in vivo. These findings reveal a collagen I–OSCAR–Hippo axis that links ECM signaling to metabolic reprogramming and suggest a potential therapeutic strategy for ccRCC.

## Introduction

As the dominant histopathological variant of renal cancer, clear cell renal cell carcinoma (ccRCC) comprises more than 70% of reported cases, with both incidence and mortality rates steadily rising on a global scale [1, 2]. Earlier studies on renal cancer largely centered on tumor cell oncogenic alterations; however, growing evidence underscores the importance of the tumor microenvironment (TME), particularly the extracellular matrix (ECM), in modulating tumor progression [3–5]. The ECM is largely produced by cancer-associated fibroblasts (CAFs) and consists of diverse structural and functional proteins [6, 7]. As the predominant ECM protein, collagen I supports tissue architecture and interacts with receptors including DDR1 and integrins to initiate multiple signaling pathways. These pathways control important biological processes like tumor cell growth, movement, and metabolism [8–10]. Recent studies have confirmed that collagen I promotes tumor progression in ccRCC [11–13]. However, the functional receptors of collagen I and the downstream signaling cascades mediating its effects in ccRCC remain unclear.

Osteoclast-associated receptor (OSCAR) belongs to the immunoglobulin superfamily and is classified within the leukocyte receptor complex (LRC). It is strongly expressed in osteoclasts and can trigger signaling pathways related to osteoclast differentiation when it binds to collagen. [14–18]. Although some bioinformatic analyses have suggested a potential association between OSCAR expression and cancer progression, its role and the pathways underlying its activity in malignant tumors have not been thoroughly investigated. [19, 20].

Disruption of Hippo pathway regulation is strongly correlated with abnormal tissue proliferation and cancer formation [21]. Upstream signals—including mechanical stress, adhesion, polarity, receptor tyrosine kinase activation, and metabolism—regulate the pathway via this kinase complex [22]. Meanwhile, recent studies have demonstrated that the Hippo signaling pathway influences the progression of multiple malignancies, including renal cell carcinoma, by regulating lipid metabolism [23, 24]. Functional inactivation of SAV1 has been shown to contribute to tumor progression in several cancer types, among them ccRCC [25, 26]. However, the upstream regulatory mechanisms controlling SAV1 function in ccRCC remain largely undefined.

Our findings demonstrate that the binding of collagen I to OSCAR induces receptor endocytosis, enabling interaction with cytoplasmic SAV1. This interaction impedes the membrane translocation of SAV1, thereby suppressing Hippo pathway activity and promoting malignant progression and lipid reprogramming in ccRCC. Based on this mechanism, we engineered a CLP-loaded LNP to competitively disrupt the collagen I–OSCAR interaction, thereby restoring Hippo pathway activity and markedly suppressing ccRCC growth.

## Materials and methods

### Patient samples

Tumor specimens together with paired adjacent normal tissues were collected from 36 patients with pathologically confirmed ccRCC at Union Hospital (Wuhan, China). All participants received nephrectomy prior to any radiotherapy or chemotherapy. Written informed consent was obtained from all participants, and the study was conducted with approval from the Ethics Committee of Union Hospital (Approval No. IEC-072).

### Human cell lines

Human renal proximal tubular epithelial clone (HK-2 (RRID: CVCL_0302)) cells, human embryonic kidney 293 T (HEK293T (RRID: CVCL_0063)) cells, and ccRCC cell lines (CAKI-1 (RRID: CVCL_0234), 786-O (RRID: CVCL_1051), and A498 (RRID: CVCL_1056)) were purchased from the American Type Culture Collection (ATCC, Manassas, VA, USA). OS-RC-2 (RRID: CVCL_1626) cells were sourced from the Cell Bank of the Chinese Academy of Sciences (Shanghai, China). All cell lines were authenticated by short tandem repeat (STR) profiling in and tested prior to the experiments to confirm the absence of mycoplasma contamination. HEK293T, HK-2, A498, and CAKI-1 cells were maintained in Dulbecco’s Modified Eagle Medium (DMEM; Gibco, Waltham, MA, USA) containing 10% fetal bovine serum (FBS; Gibco) and incubated at 37 °C in a humidified environment with 5% CO₂. OS-RC-2 and 786-O cells were cultured in RPMI-1640 medium (Gibco) under identical conditions.

### Transfection and infection

For transient transfection, ccRCC cells were subjected to transfection with Lipofectamine™ 3000 (Invitrogen, Waltham, MA, USA) according to the manufacturer’s guidelines. Plasmids were transfected into HEK293T cells using polyethyleneimine (PEI, Polysciences, Niles, IL, USA) following the standard protocol. For RNA interference, cells were transfected with siRNAs using Lipofectamine™ RNAiMAX (Invitrogen) following the manufacturer’s protocol. Cells were collected 48 hours after transfection for subsequent experiments.

Lentiviral shRNA vectors targeting OSCAR and SAV1 were constructed in PLKO.1-TRC and pFB-8 backbones, respectively. HA-tagged SAV1, MST1, LATS1, MOB1, NF2, and KIBRA (pFC vector) were synthesized by Focus Bioscience, Inc. (Shanghai, China). Full-length and truncated FLAG-tagged OSCAR plasmids (pcDNA3.1 vector) were synthesized by GentleGen (Suzhou, China). siRNAs targeting DDR1, DDR2, ITGA1, ITGB1, ITGA2, ITGA11, GP6, and OSCAR were synthesized by JTSBIO Co., Ltd (Wuhan, China). All siRNA sequences and plasmid information are listed in Supplementary Table S2.

### Collagen I (Coll I) treatment in multi-well plates

Commercial Rat Tail Collagen I (Gibco) was diluted in sterile PBS/glycerol solution (0.5 M glycerol, 0.2 M sodium phosphate, pH 7.4) and applied to cell culture experiments in multi-well plates. A final concentration of 50 μg/mL Collagen I was applied to the cells. For experiments using 6-well plates, each well received 100 μg of Collagen I protein (prepared in PBS/glycerol) in 2 mL of culture medium.

### Real-time PCR

RNA from ccRCC tissues and cell lines was isolated with Trizol reagent (Beyotime, Shanghai, China). Complementary DNA (cDNA) synthesis was carried out using a First Strand cDNA Synthesis Kit (Vazyme, Nanjing, China), and gene expression was quantified by qPCR employing a SYBR Green qPCR Kit (Vazyme) on a qTOWER thermal cycler (Analytik Jena, Jena, Germany). Quantitative PCR (qPCR) was performed starting with a 3-minute denaturation at 95 °C, followed by 45 amplification cycles of 95 °C for 5 s and 62 °C for 30 s. Relative gene expression levels were calculated using the 2^–^ΔΔCt approach. All primers were custom-synthesized by Tsingke (Beijing, China) and their sequences are provided in Supplementary Table S3.

### Western blotting and co-IP

Proteins (30 μg) from ccRCC tissues or cell lines were resolved on polyacrylamide gels and transferred to PVDF membranes (Roche, Basel, Switzerland) for further analysis. Primary antibodies were applied to the membranes and incubated at 4 °C for 12–14 h, after which the membranes were incubated with corresponding secondary antibodies at room temperature for 2 h. Protein signals were detected using a ChemiDoc imaging system (Bio-Rad, Hercules, CA, USA).

For co-immunoprecipitation experiments, 293 T cells were first lysed to obtain total protein extracts. The lysates were then incubated with either control IgG or the designated primary antibodies at 4 °C for 12 to 14 h. Protein A/G magnetic beads (MedChemExpress, Monmouth Junction, NJ, USA) were added to capture the antibody–protein complexes and allowed to bind for 2 h at room temperature. Following incubation, the beads were thoroughly washed to eliminate nonspecific interactions, and the precipitated proteins were subsequently subjected to Western blot analysis.A comprehensive list of the antibodies employed is provided in Supplementary Table S4.

### RNA-sequencing (RNA-seq)

RNA sequencing was performed using the Illumina NovaSeq platform (Shanghai Personal Biotechnology Co., Shanghai, China). Total RNA was initially isolated and purified using the GeneJET RNA Purification Kit (Thermo Scientific, Waltham, MA, USA), and its integrity was evaluated with a Bioanalyzer system (Agilent Technologies, Santa Clara, CA, USA) employing the RNA 6000 Nano Chip. Enrichment of mRNA was performed using oligo(dT) magnetic beads (NEB, Ipswich, MA, USA), followed by construction of strand-specific cDNA libraries using the NEBNext Ultra Directional RNA Library Prep Kit for Illumina (NEB). Qualified libraries were sequenced with 150 bp paired-end reads on an Illumina platform at Novogene (Beijing, China), with four biological replicates included for each condition.

### Cell viability and anti-proliferative activity assay

To evaluate cell proliferation and the anti-proliferative effect of CCP-LNP, ccRCC cells were seeded into 96-well plates at a density of 2000 cells per well and incubated for 24 h at 37 °C in a humidified environment containing 5% CO₂ to allow cell attachment. For the proliferation assay, cells were refreshed with medium and evaluated at 0, 24, 48, 72, and 96 h using the Cell Counting Kit-8 (Vazyme).Cells were incubated with 10 μL CCK-8 solution in 100 μL DMEM per well at 37 °C in the dark, and the absorbance at 450 nm was measured with a NanoDrop spectrophotometer.

The anti-proliferative effect of CCP-LNP was evaluated by treating cells with different concentrations of the nanoparticles for 72 h. Following treatment, cells were washed with PBS and subjected to CellTiter-Glo® luminescent assay (Promega, USA) according to the manufacturer’s protocol. Luminescence signals were measured using a NanoDrop spectrophotometer.

### Colony formation assays

For colony formation assays, ccRCC cells were plated in 6-well plates at 1 × 10³ cells per well. After 14 days of culture, cells were washed with PBS, fixed in methanol, and stained with 0.05% crystal violet (Biosharp, Hefei, China). Representative colonies were then imaged.

### Transwell assays

Before the assay, ccRCC cells and HLECs were serum-starved for 24 h. Transwell inserts (Corning, Corning, NY, USA) were pretreated with Matrigel (Corning) in the upper chambers, after which 1 × 10⁵ cells were seeded. After incubating overnight, cells that migrated to the underside of the membrane were fixed with methanol, stained using 0.05% crystal violet, and imaged in randomly chosen fields.

For migration assays, Transwell inserts without Matrigel coating were used under the same conditions to assess migratory capacity.

### TG detection

Cells were grown in 6 cm culture dishes to approximately 50% confluence and harvested after 24 h. For tissue specimens, 0.1 g of subcutaneous tumor tissue or cell pellets were mechanically homogenized in 0.9 mL Triton X-100 (Beyotime) and centrifuged at 600 × g for 10 min. Triglyceride levels in the resulting supernatants were quantified using a commercial Triglyceride Assay Kit (Jiancheng, Nanjing, China) following the manufacturer’s protocol.

### BODIPY (493/503) staining

For fluorescence imaging, ccRCC cells were rinsed with PBS, permeabilized using Triton X-100, and stained with 1 μM BODIPY 493/503 dye for 30 min at room temperature in the dark. Cells were then washed twice with PBS. Nuclei were counterstained with DAPI, and intracellular lipid droplets (LDs) were visualized using a confocal laser scanning microscope (excitation: 493 nm; emission: 503 nm). For flow cytometry, cells were trypsinized, resuspended in complete medium, washed once with PBS, and stained with 1 μM BODIPY 493/503 for 15 min at room temperature in the dark. Stained cells were analyzed on a Becton Dickinson flow cytometer, and data were processed using FlowJo software.

### Immunofluorescence

ccRCC cells were plated onto 15 mm confocal dishes (Biosharp) at 1 × 10⁵ cells per coverslip. After PBS washes, Cells grown on coverslips were initially fixed in 4% methanol for 10 min to preserve cellular structures, followed by permeabilization with 0.5% Triton X-100 for 20 min at ambient temperature to allow antibody access. After thorough washing, samples were incubated with primary antibodies overnight at 4 °C. Subsequently, fluorescently conjugated secondary antibodies were applied for 2 h at room temperature. Nuclei were counterstained with DAPI (Beyotime) for half an hour, and confocal images were acquired using a Nikon fluorescence microscope. A complete list of antibodies is provided in Supplementary Table S4.

### Lipidomics analysis

Lipidomic analysis was performed by Shanghai Personal Biotechnology Co., Ltd. CAKI-1 cells from each experimental group were expanded according to the service requirements, with four biological replicates per group across a total of eight 10 cm culture dishes. Cells were harvested, snap-frozen in liquid nitrogen, and pulverized under cryogenic conditions. Lipids were isolated with a chloroform:methanol mixture (2:1, v/v), subsequently dissolved in acetonitrile, and subjected to liquid chromatography–tandem mass spectrometry (LC–MS/MS) analysis, maintaining a flow rate of 0.3 mL/min. Lipid species were identified based on their mass-to-charge (m/z) ratios and characteristic fragmentation patterns. Raw data were processed with Lipidyzer software for denoising, peak detection, and alignment, and the relative abundance of each lipid species was quantified for downstream analyses.

### Animal models

Male BALB/c nude mice, aged four weeks and weighing 22–35 g, were purchased from Hubei Beiente Biotechnology Co., Ltd. and maintained under specific pathogen-free (SPF) conditions at the Wuhan Laboratory Animal Center, Tongji Medical College. Each nude mouse was assigned a unique random number, and animals were allocated to the experimental groups according to the ascending order of these random numbers. Blinding was applied during data collection and outcome assessment, and the investigator was unaware of group assignments throughout the experiment.

The subcutaneous tumor model was generated by injecting 2 × 10⁶ CAKI-1 cells in 100 μL culture medium into the right-side flank of the nude mice (*n* = 5). Euthanasia was performed on day 44 after cell injection, or earlier if tumors exceeded 1.5 cm in diameter, using CO₂ inhalation followed by cervical dislocation.The tumors were subsequently removed, imaged, and their weights recorded.

The orthotopic renal tumor model was established by injecting 2 × 10⁶ CAKI-1-LUC cells under the exposed renal capsule, whereas the metastatic model was generated via tail vein injection of 4 × 10⁶ A498 cells (*n* = 5). Tumor growth and dissemination were monitored by bioluminescence imaging using the LagoX system (Spectral Instruments Imaging, Tucson, AZ, USA) following intraperitoneal administration of d-luciferin.

For biodistribution assessment, liposomes were labeled with Cy7 (MedChemExpress) to generate CCP-LNP-Cy7. When tumor volumes reached 400–500 mm³, tumor-bearing mice were randomly assigned to groups (*n* = 3) and intravenously injected with either CLP-Cy7 or CCP-LNP-Cy7. In vivo fluorescence imaging was conducted at 0, 3, 8, and 24 h post-injection using the LagoX system (Spectral Instruments Imaging). At 24 h, mice were sacrificed, and major organs (heart, liver, spleen, lung, kidney) along with tumors were harvested for ex vivo imaging.

To assess therapeutic efficacy, tumor-bearing mice with palpable tumors on day 10 post-inoculation were randomly assigned to five groups (*n* = 5) and intravenously treated with PBS (control), free CLP, or CCP-LNP at an equivalent CLP dose of 2 mg·kg⁻¹ every 4 days for a total of five injections. Tumor volume and body weight were recorded every 4 days. Mice were sacrificed on day 44 or once tumors reached 1.5 cm in diameter, and both tumors and key organs were harvested for subsequent analyses. Tumor volume was calculated using the formula: V (mm³) = L × W² / 2, where L and W denote tumor length and width, respectively.

### Toxicity evaluation

Mouse body weight was monitored throughout the in vivo antitumor study to evaluate systemic toxicity. Upon completion of the study, animals were sacrificed, and the heart, liver, spleen, lungs, and kidneys were harvested for histological examination. Organs were fixed in 10% neutral formalin, paraffin-embedded, sectioned, and stained with H&E. Tissue sections were examined under a microscope by a qualified toxicologist to identify any pathological changes or organ damage.

### H&E staining

Tissue samples embedded in paraffin were sliced and underwent routine dewaxing and rehydration. Following washes, the sections were stained with hematoxylin for 10 min and differentiated, then counterstained with eosin for 5 min. Processed slides were imaged using a Leica microscope to document histological features.

### IHC

IHC analysis was performed on tissue samples from subcutaneous tumor models and clinical ccRCC patients. Tissue samples were initially fixed in 4% paraformaldehyde and then processed through dehydration and paraffin embedding. Embedded blocks were sectioned and the slices underwent deparaffinization and rehydration. Sections were subsequently treated with specific primary antibodies, followed by incubation with the appropriate secondary antibodies. Stained slides were visualized and representative images were obtained using a Leica microscope. All patients provided written consent allowing their tissue samples to be used in this study. A complete list of antibodies is provided in Supplementary Table S4.

### Oil Red O Staining

Fresh tumor specimens were first embedded in optimal cutting temperature (OCT) compound and then cryosectioned into 8–10 μm slices. The sections were allowed to air-dry, fixed in 4% paraformaldehyde for 10 min, washed with PBS, and subsequently stained in freshly prepared 0.3% Oil Red O solution for 15–30 min in the dark. Excess dye was removed with 70% ethanol and PBS washes. Nuclei were counterstained with hematoxylin. Intracellular lipid accumulation was examined and documented using light microscopy.

### Preparation of Liposomes

Liposomes encapsulating CLP (GPOGPOGPOGPOGPOGPOGPAGFOGPOGPOGPOGPOGPOG) were prepared using the ethanol injection method. First, DSPE-PEG-MAL (MedChemExpress)and CBP(CQDSETRTFY) were mixed at a 1:1 molar ratio and incubated at room temperature for 6 h to obtain DSPE-PEG-CBP. Separately, DOPE (7.44 mg,MedChemExpress), SPC (7.6 mg,MedChemExpress), and CHEMS (5.13 mg,MedChemExpress) were dissolved in 1 mL of absolute ethanol to form a lipid phase. An aqueous solution was prepared by dissolving 0.5 mg of CLP together with 1.12 mg of DSPE-PEG-CBP in 4 mL of PBS at pH 7.4.

The ethanol-based lipid solution was then slowly injected into the aqueous CLP solution under continuous ultrasonication (room temperature, 40 kHz, 30 min) to form a uniform emulsion. The mixture was subsequently subjected to ultrafiltration to remove free drug and organic solvent. Blank liposomes (Ctrl-LNP) without CLP were prepared using the same method as a control.

### Transmission electron microscopy (TEM) analysis of liposome morphology

The morphology of liposomes was examined using a transmission electron microscope (HITACHI, Tokyo, Japan). Briefly, 3 μL of liposome suspension was dropped onto a glow-discharged 300-mesh carbon-coated copper grid. Excess liquid was carefully blotted off using filter paper. For contrast enhancement, the samples were subsequently subjected to negative staining using 1% (w/v) phosphotungstic acid.After drying at room temperature, the grids were imaged under TEM at an acceleration voltage of 80 kV.

### Physicochemical characterization of liposomes

Liposome physicochemical properties, including particle size, and zeta potential, were measured at 25 °C using a NanoBrook 900Plus PALS instrument (Brookhaven Instruments Corporation, New York, NY, USA). Samples were appropriately diluted with distilled water and filtered through a 0.2 μm membrane prior to measurement. All assays were performed in triplicate to ensure reproducibility.

### In vitro drug release

A 1 mL aliquot of CCP-LNP-Cy7 was loaded into a dialysis bag (MWCO 6–8 kDa) and immersed in 40 mL PBS (pH 6.5 or 7.4) containing 0.5% Tween-80. The system was incubated at 37 °C with gentle shaking (140 rpm). At predetermined time points (0, 1, 2, 4, 8, 12, 24, and 48 h), buffer samples were collected, and the fluorescence of Cy7 was measured (excitation: 750 nm; emission: 770 nm) to assess the release profile of CLP.

### In vitro stability of CCP-LNP

The in vitro stability of CCP-LNP was evaluated under two conditions: storage at 4 °C for 90 days and incubation in fetal bovine serum (FBS) at 37 °C for 72 h. For storage stability, CCP-LNP was maintained at 4 °C, and its particle size was measured at predetermined time points over a period of 90 days.

For serum stability, CCP-LNP was mixed with an equal volume of FBS and incubated at 37 °C with gentle shaking (45 rpm). Samples were collected at defined time points (0, 1, 2, 4, 8, 12, 24, and 48 h) to monitor changes in particle size and evaluate colloidal stability. The evaluation was performed according to previously described protocols.

### Bioinformatics and statistical analyses

Publicly available RNA-seq data for ccRCC were obtained from TCGA-KIRC (https://portal.gdc.cancer.gov). The DESeq2 package in R (R Core Team) was employed to identify DEGs, applying cutoffs of an adjusted *P*-value less than 0.05 and an absolute log₂ fold change greater than 1.5. Overall survival in relation to target gene mRNA expression was analyzed using Kaplan–Meier curves, with survival duration measured from the initial pathological diagnosis until either death from any cause or the most recent follow-up. Patients were stratified into high- and low-expression groups according to the median expression level. GSEA and KEGG pathway analysis in R were used to perform functional enrichment. Statistical analyses were conducted using GraphPad Prism 9.0 and SPSS 26.0. Unless otherwise stated, quantitative data are presented as mean ± SD from at least three independent biological replicates. Appropriate statistical tests were applied as indicated in the figure legends, including Student’s t-test, Wilcoxon test, Mann–Whitney U test, Spearman correlation, one-way ANOVA, Kruskal–Wallis test, and log-rank test. For ANOVA analyses, suitable post-hoc multiple comparison tests were applied where appropriate. For t-tests, a minimum sample size of *n* > =3 was considered. Experiments were performed in triplicate or more unless otherwise indicated.

## Results

### Upregulation of collagen I and its receptor OSCAR in ccRCC is associated with adverse clinical outcome and tumor progression

**Fig. 1:**
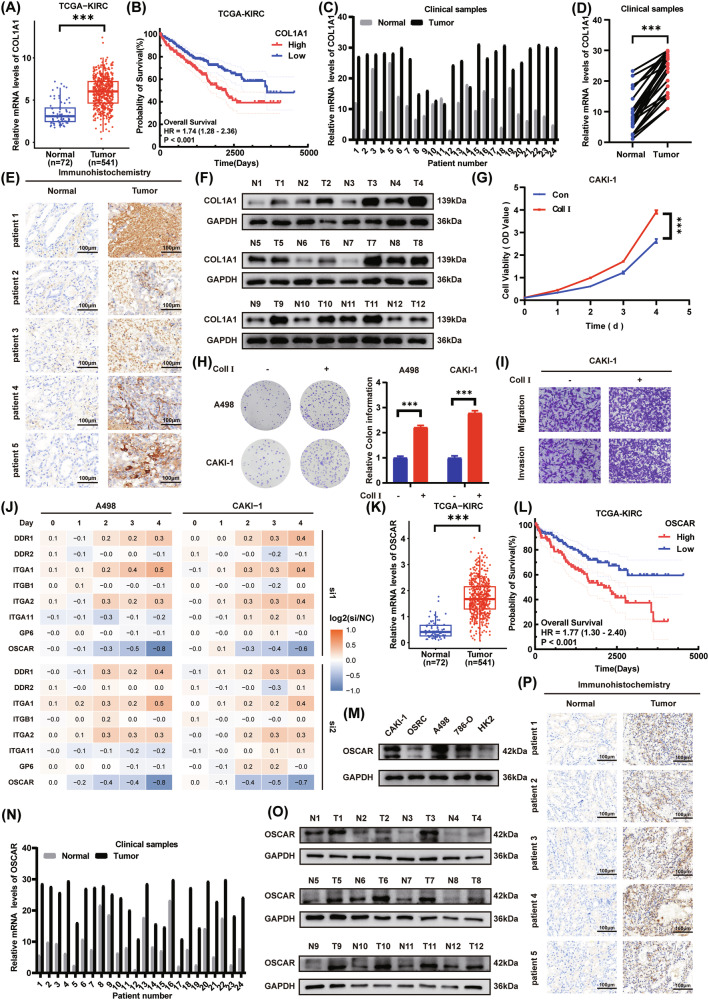
Upregulation of collagen I and its receptor OSCAR in ccRCC is associated with adverse clinical outcome and tumor progression. **A** Analysis of TCGA-KIRC dataset comparing COL1A1 mRNA levels between ccRCC tissues and adjacent normal tissues (Student’s t-test). **B** Kaplan–Meier survival analysis was performed to evaluate the relationship between COL1A1 expression and overall survival in ccRCC patients. Patients were divided into high- and low-expression cohorts based on the median COL1A1 level, and differences in survival between groups were assessed using the log-rank test. **C**, **D** Relative COL1A1 mRNA expression in paired tumor and adjacent normal tissues from 24 ccRCC patients (Student’s t-test). **E** Representative immunohistochemistry (IHC) images showing COL1A1 expression in tumor versus normal tissues from five patients. **F** Western blot analysis of COL1A1 protein in paired tumor and adjacent normal tissues from 12 patients. **G** Proliferation of CAKI-1 cells treated with collagen I or control was monitored over time using the CCK-8 assay (*n* = 3, student’s t-test). **H** Colony formation assay comparing collagen I-treated and control cells (*n* = 3, one-way ANOVA followed by Dunnett’s post-hoc test). **I** Transwell migration assay of CAKI-1 cells exposed to collagen I versus control (*n* = 3). **J** Heatmap showing the proliferation of receptor-knockout ccRCC cells and control cells upon type I collagen stimulation assessed by CCK-8 assay (*n* = 3). **K** TCGA-KIRC dataset comparison of OSCAR mRNA expression between tumor and normal tissues (Student’s t-test). **L** Kaplan–Meier survival analysis of OSCAR expression and overall survival in ccRCC patients, divided into high and low expression groups based on the median value (log-rank test). **M** OSCAR protein expression in ccRCC versus normal renal epithelial cell lines. **N** OSCAR mRNA levels in paired tumor and normal tissues from 24 ccRCC patients. **O** OSCAR protein levels in paired tumor and adjacent tissues from 12 ccRCC patients. **P** Representative IHC images depicting OSCAR expression in both cancerous and matched non-cancerous tissues from five patients. All experiments were performed in triplicate unless otherwise stated. Statistical significance: *P < 0.05, **P < 0.01, ***P < 0.001.

Collagen type I is a heterotrimer consisting of two α1(I) chains encoded by COL1A1 and one α2(I) chain encoded by COL1A2, forming a triple-helical structure [27]. To assess collagen I expression in ccRCC, collagen, type I, alpha 1(COL1A1) levels were initially examined in the TCGA-KIRC dataset, revealing a marked upregulation in tumor tissues (Fig. 1A and Fig. S1A). Analysis using the Kaplan-Meier method revealed that high COL1A1 levels were significantly linked to worse overall survival in patients with ccRCC (Fig. 1B). This finding was further validated by qPCR, which showed that COL1A1 expression was markedly elevated in clinical ccRCC specimens relative to matched normal tissues (Fig. 1C, D). Immunohistochemical (IHC) staining and Western blotting also confirmed that COL1A1 protein levels were upregulated in tumor tissues versus matched normal controls (Fig. 1E, F).Overall, COL1A1 was found to be overexpressed in ccRCC and correlated with poor prognosis. To better mimic the effects of collagen I in the ECM on tumor cells, exogenously added collagen I was used to culture A498 and CAKI-1 cells. CCK-8 and colony formation assays showed that collagen I substantially increased the growth of ccRCC cells (Fig. 1G, H and Fig. S1B–D). Furthermore, transwell migration and invasion assays showed that collagen I markedly promoted the motility and invasiveness of ccRCC cells (Fig. 1I and Fig. S1E).

Collagen I influences various tumor cell functions by interacting with specific cell surface receptors and triggering downstream signaling cascades [28]. To systematically evaluate potential receptors mediating the pro-tumorigenic effects of collagen I in ccRCC, a series of known collagen receptors, including DDR1, DDR2, GP6, ITGA1, ITGA2, ITGA11, ITGB1, and OSCAR, were individually targeted for knockdown in A498 and CAKI-1 ccRCC cells (Fig. S2A, B) [10, 29]. CCK-8 viability assays revealed that among all tested receptors, only knockdown of OSCAR significantly impaired the proliferative response of ccRCC cells to collagen I stimulation, suggesting that OSCAR may be required for collagen I-induced cell proliferation (Fig. 1J). Based on this finding, OSCAR was also knocked down in ccRCC cells cultured in the absence of collagen I, which did not result in significant change in proliferation, indicating that OSCAR itself is not essential for basal proliferative activity (Fig. S2C, D). Furthermore, survival analysis based on the TCGA-KIRC database further reveals that among collagen I receptors, only high OSCAR expression demonstrates a significant association with decreased overall survival in ccRCC (Fig. 1K and Fig. S3A–G).Endogenous co-immunoprecipitation (co-IP) experiments demonstrate robust binding between OSCAR and collagen I (Fig. S3H, I). Given the possibility of compensatory receptor usage, we next examined whether OSCAR knockdown affects the transcription of other known collagen I receptors. qPCR analysis revealed no significant changes in the mRNA levels of collagen receptors following OSCAR depletion in ccRCC cells (Fig. S3J, K), suggesting an absence of compensatory regulation at the transcriptional level under the experimental conditions tested. To clarify the role of OSCAR in ccRCC, its expression levels were first evaluated. TCGA-KIRC dataset analysis indicated that OSCAR levels are considerably higher in ccRCC tissues compared with normal controls (Fig. 1K and Fig. S4A). Analysis using the Kaplan-Meier method revealed that high OSCAR levels were significantly linked to worse overall survival in patients with ccRCC (Fig. 1L). Correlation analysis with clinical features indicated that OSCAR expression was positively associated with pathological grade and clinical stage (Fig. S4B–F). This observation was corroborated by qPCR and Western blot assays in clinical specimens and cell lines, revealing significant upregulation of OSCAR mRNA and protein in ccRCC tissues relative to matched normal tissues. In addition, compared with renal tubular epithelial cell (HK2), OSCAR expression was markedly higher in ccRCC cell lines, as reflected at both mRNA and protein levels (Fig. 1M–O and Fig. S4G, H). IHC staining further confirmed that OSCAR protein expression was substantially increased within tumor tissues compared with matched normal tissues (Fig. 1P). Analysis using Kaplan-Meier curves indicated that elevated OSCAR expression was significantly associated with poorer disease-specific survival (DSS) in ccRCC patients (Fig. S4I), while ROC curve analysis demonstrated that OSCAR has high accuracy in differentiating ccRCC from normal tissues, with a relatively high AUC (Fig. S4J). Multivariate Cox regression revealed OSCAR to be an independent unfavorable prognostic factor for ccRCC (Table S1). Altogether, these observations point to OSCAR as a central mediator of the cancer-driving effects of collagen I in ccRCC.

### Collagen I promotes ccRCC progression through OSCAR

**Fig. 2:**
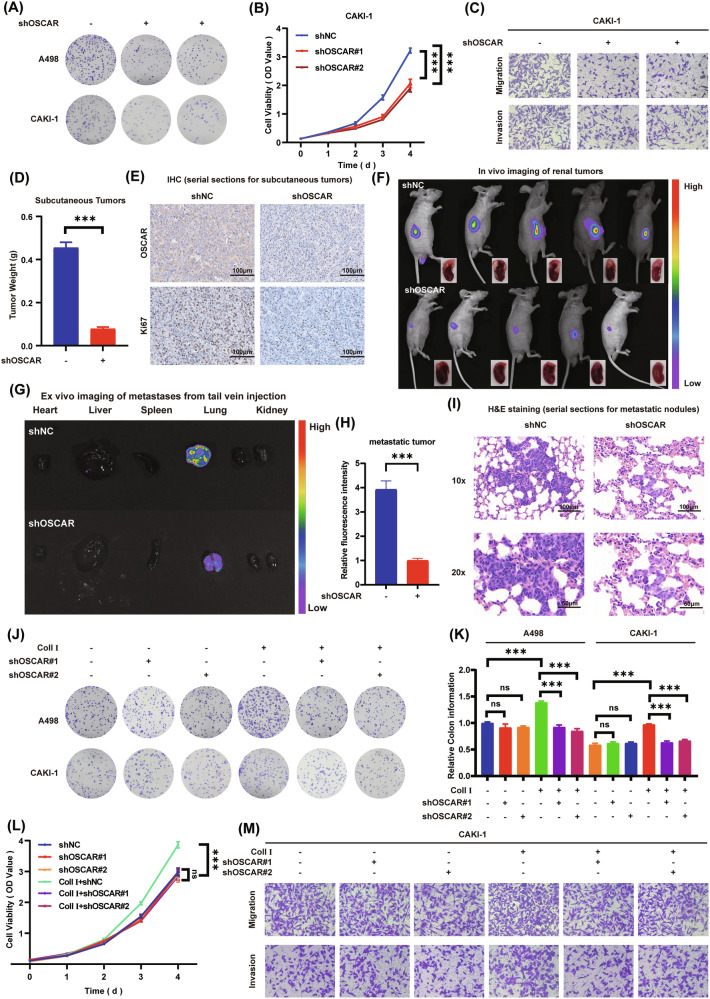
Collagen I promotes ccRCC progression through OSCAR. **A** Colony formation assay comparing OSCAR-knockdown ccRCC cells with control cells following collagen I stimulation (*n* = 3). **B** Proliferation curves of OSCAR-knockdown and control CAKI-1 cells under collagen I stimulation, assessed by CCK-8 assay (*n* = 3, two-way ANOVA with appropriate post-hoc tests). **C** Transwell migration assay of OSCAR-silenced and control CAKI-1 cells treated with collagen I (*n* = 3). **D** Tumor weights from nude mice bearing subcutaneous OSCAR-knockdown or control CAKI-1 xenografts at study endpoint (*n* = 5, Student’s t-test). **E** Representative immunohistochemistry (IHC) images showing Ki67 and OSCAR expression in subcutaneous tumors from both groups (*n* = 5). **F** In vivo bioluminescence imaging of orthotopic renal tumors in OSCAR-knockdown and control mice, four weeks post-injection (*n* = 5). **G**, **H** Bioluminescent imaging of metastatic organs in OSCAR-knockdown and control mice following tail vein injection of A498 cells (*n* = 5, Student’s t-test). **I** Representative H&E staining of lung tissues from metastatic models in both groups. **J**, **K** Colony formation assays of ccRCC cells under indicated treatment conditions (*n* = 3, one-way ANOVA followed by Dunnett’s post-hoc test). **L** Proliferation curves of ccRCC cells under the same treatment conditions, evaluated by CCK-8 assay (*n* = 3, two-way ANOVA with appropriate post-hoc tests). **M** Transwell migration assay of CAKI-1 cells under indicated conditions (*n* = 3). Data are shown as mean ± SD from at least three independent experiments. Statistical significance: *P < 0.05, **P < 0.01, ***P < 0.001.

To examine whether collagen I influences OSCAR expression, qPCR was conducted in combination with Western blotting, showing that collagen I addition had no effect on OSCAR mRNA or protein levels (Fig. S5A, B).To further explore the role of OSCAR, renal carcinoma cell lines A498 and CAKI-1 with enhanced expression and targeted knockdown were generated (Fig. S5C–F). Under collagen I stimulation, functional assays were conducted. Clonogenic assays showed that knockdown of OSCAR considerably impaired colony formation, while OSCAR overexpression promoted the proliferative potential of colonies (Fig. 2A Fig. S5G, H). Consistently, CCK-8 assays demonstrated that knockdown OSCAR markedly suppressed cell proliferation, while OSCAR overexpression increased cell viability (Fig. 2B and Fig. S5I, J). Transwell assays demonstrated that OSCAR knockdown reduced ccRCC cell migration and invasion, whereas elevated OSCAR levels markedly promoted the motility and invasive behavior of A498 and CAKI-1 cells (Fig. 2C and Fig. S5K–P).Subsequently, CAKI-1 cells with OSCAR knockdown or control vectors were subcutaneously injected into 4-week-old BALB/c nude mice.The results showed that tumors in the OSCAR knockdown group exhibited significantly reduced volume and weight compared to controls, and Ki-67 staining indicated decreased proliferative activity (Fig. 2D, E and Fig. S6A, B). An orthotopic renal tumor model was then established using CAKI-1 cells with or without OSCAR knockdown. In vivo bioluminescence imaging, kidney weight measurement, and hematoxylin-eosin (H&E) staining collectively demonstrated that OSCAR knockdown markedly suppressed orthotopic tumor growth relative to controls (Fig. 2F and Fig. S6C–E). Furthermore, a tail vein metastasis model was constructed using A498 cells with stable OSCAR knockdown or control, and both in vivo imaging and H&E staining revealed that metastatic capacity of ccRCC cells was significantly inhibited by OSCAR knockdown (Fig. 2G–I).

After confirming through both cellular and animal experiments demonstrating that OSCAR markedly enhances tumor growth and tumor dissemination in the presence of collagen I, we then aimed to investigate whether the pro-tumorigenic effects of collagen I are mediated through OSCAR. Functional experiments were conducted using ccRCC cells with or without OSCAR knockdown, in the presence or absence of exogenous collagen I. Colony formation assays revealed that knockdown of OSCAR effectively blocked the collagen I-induced enhancement of clonogenic capacity, whereas OSCAR knockdown had no significant effect in the absence of collagen I (Fig. 2J, K). Consistent trends were observed in both CCK-8 cell viability and transwell migration and invasion assays (Fig. 2L–M and Fig. S7A–D). Collectively, these findings demonstrate that collagen I facilitates the proliferation and metastatic activity of ccRCC cells mainly via OSCAR.

### Collagen I promotes ccRCC progression through OSCAR-mediated inhibition of the Hippo pathway

**Fig. 3:**
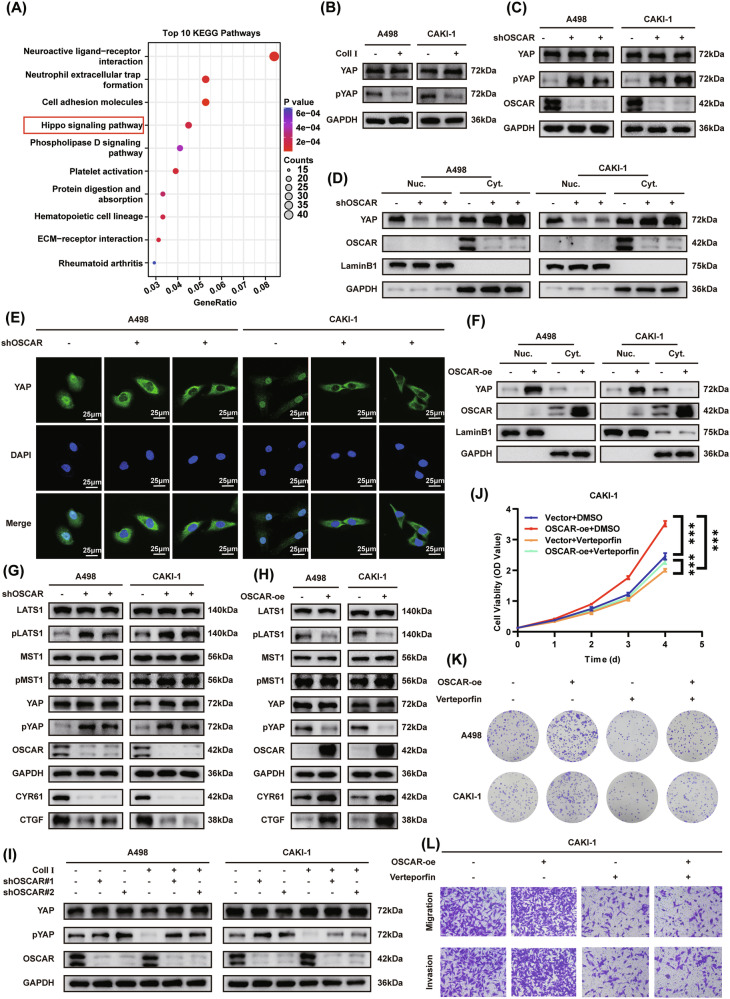
Collagen I promotes ccRCC progression through OSCAR-mediated inhibition of the Hippo pathway. **A** Enrichment analysis of DEGs between shNC and shOSCAR groups using KEGG, highlighting the top ten signaling pathways. **B** Collagen I-treated and control cells were analyzed by Western blot to compare levels of phosphorylated and total YAP. **C** Western blot of total YAP and p-YAP in OSCAR-knockdown and control cells after collagen I treatment. **D** Western blot assessing YAP subcellular distribution in OSCAR-deficient cells following collagen I exposure. **E** Immunofluorescence analysis confirming YAP localization changes in OSCAR-knockdown cells treated with collagen I. **F** Western blot showing YAP localization in OSCAR-overexpressing cells after collagen I stimulation. **G**, **H** Western blotting was performed to assess the phosphorylation status and total protein levels of LATS1, MST1, and YAP, along with the expression of downstream targets CYR61 and CTGF in OSCAR-knockdown or OSCAR-overexpressing cells following collagen I treatment. **I** Western blot evaluating p-YAP and total YAP in cells subjected to indicated treatments. **J** CCK-8 proliferation assay of ccRCC cells under different treatments (*n* = 3, two-way ANOVA with appropriate post-hoc tests). **K** Colony formation assay under different conditions (*n* = 3). **L** Transwell migration assay of CAKI-1 cells with indicated treatments (*n* = 3). Results represent at least three independent experiments. Data are shown as mean ± SD. Statistical significance: *P < 0.05, **P < 0.01, ***P < 0.001.

RNA-seq analysis of CAKI-1 cells under collagen I stimulation identified extensive transcriptional alterations upon OSCAR knockdown, including 631 upregulated and 553 downregulated genes (*P* < 0.05, |log₂FC | > 1; Fig. S8A). Pathway enrichment analysis highlighted the Hippo signaling pathway as a top-ranked pathway, implicating it as a key downstream effector of OSCAR signaling (Fig. 3A). Given previous reports showing that collagen I can modulate Hippo pathway activity, the expression of key Hippo-related genes was examined by qPCR [10, 29, 30]. The classical YAP target genes Connective Tissue Growth Factor (CTGF) and Cysteine-Rich Angiogenic Inducer 61 (CYR61) were markedly downregulated upon OSCAR knockdown (Fig. S8B, C), while their expression was markedly upregulated in OSCAR-overexpressing cells (Fig. S8D, E). Furthermore, treatment with collagen I induced upregulation of CTGF and CYR61 compared to untreated controls (Figure S8F, G). Notably, the mRNA level of YAP itself remained unchanged across all treatment groups.To clarify the mechanism underlying the altered expression of YAP target genes, Western blotting was performed to evaluate alterations in YAP activity.Collagen I treatment had no impact on total YAP protein levels, whereas it markededly reduced its phosphorylated form (Fig. 3B). Likewise, OSCAR knockdown or overexpression had no effect on total YAP protein levels.; however, overexpression of OSCAR significantly decreased phosphorylated YAP (p-YAP), while OSCAR knockdown led to an increase in p-YAP, indicating that OSCAR may enhance YAP activity by reducing its phosphorylation (Fig. 3C;and Fig. S9A). Since YAP activity is highly dependent on its subcellular localization, cytoplasmic-nuclear fractionation and immunofluorescence staining were performed. Both Western blot and immunofluorescence results demonstrated that OSCAR knockdown reduced nuclear translocation of YAP, whereas OSCAR overexpression promoted its nuclear accumulation (Fig. 3D–F and Fig. S9B).To pinpoint the regulatory node of OSCAR within the Hippo pathway, both the protein expression and phosphorylation status of central Hippo pathway components were examined. OSCAR knockdown had no effect on either total or phosphorylated MST1/2 levels, but markedly increased the phosphorylation levels of LATS1/2 and YAP, accompanied by a significant reduction in CTGF and CYR61 expression, indicating Hippo pathway activation (Fig. 3G). Conversely, OSCAR overexpression suppressed the phosphorylation of both LATS1/2 and YAP, and enhanced the levels of downstream YAP target genes (Fig. 3H). Collectively, these results indicate that OSCAR regulates the Hippo pathway primarily by modulating LATS1/2 activity, thereby influencing YAP phosphorylation and transcriptional activity.

To assess the pivotal function of the Hippo pathway in the effects driven by the collagen I–OSCAR axis, the YAP-TEAD inhibitor Verteporfin was administered to OSCAR-overexpressing ccRCC cells. CCK-8 and colony formation assays confirmed that YAP-TEAD inhibition effectively abrogated the proliferative and clonogenic advantages conferred by OSCAR overexpression (Fig. 3J, K and Fig. S9C, D). Furthermore, transwell assays demonstrated that YAP inhibition significantly reversed the enhanced migratory and invasive capabilities induced by OSCAR overexpression (Fig. 3L and Fig. S9E–G). Collectively, the data indicate that the tumor-promoting effects of OSCAR depend on its regulation of YAP activity.

### Collagen I–OSCAR axis promotes lipid reprogramming in ccRCC by inhibiting the Hippo pathway

**Fig. 4:**
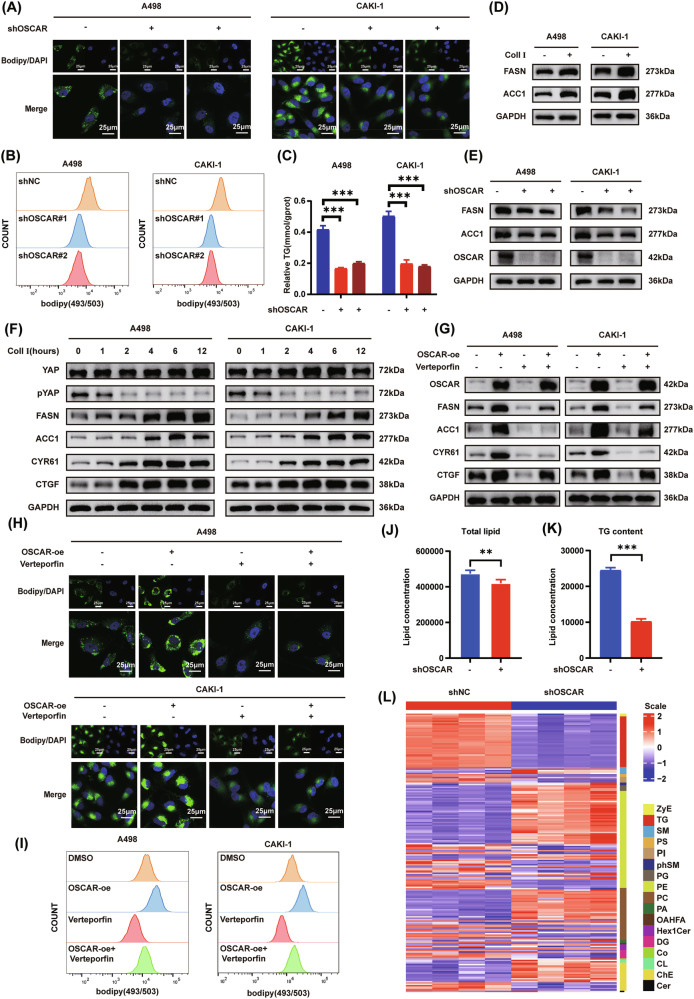
Collagen I–OSCAR axis promotes lipid reprogramming in ccRCC by inhibiting the Hippo pathway. **A**, **B** Bodipy (498/503) staining and confocal/flow cytometry were used to assess lipid droplet accumulation in collagen I-treated OSCAR-knockdown and control ccRCC cells (*n* = 3). **C** Triglyceride (TG) content (mmol/g prot) was quantified using a TG assay kit under the same conditions (*n* = 3, one-way ANOVA followed by Dunnett’s post-hoc test). **D**, **E** Western blot analysis of FASN and ACC1 in collagen I-treated versus control cells, and in OSCAR-knockdown cells following collagen I treatment. **F** Time-dependent Western blot of p-YAP, total YAP, FASN, ACC1, CYR61, and CTGF after collagen I stimulation. **G** OSCAR, FASN, and ACC1 protein expression under different treatment conditions following collagen I exposure. **H**, **I** Confocal imaging and flow cytometry analysis of Bodipy-stained lipid droplets under various treatment conditions involving collagen I (*n* = 3). **J**, **K** Lipidomic quantification of total lipids and triglycerides in collagen I-treated OSCAR-knockdown and control cells (*n* = 4, t-test). **L** Lipidomics profiling of changes in lipid species induced by collagen I under OSCAR-knockdown and control conditions. Data represent at least three independent experiments. Statistical significance: *P < 0.05, **P < 0.01, ***P < 0.001.

ccRCC is characterized by intracellular lipid droplet buildup, which is considered a hallmark pathological feature of the disease [31, 32]. During cell culture experiments, we observed that collagen I treatment resulted in a substantial increase in lipid droplet content in ccRCC cells (Fig. S10A). Additionally, GSEA of the TCGA-KIRC dataset indicated a strong association between OSCAR expression and lipid metabolism in ccRCC (Fig. S10B). To experimentally validate this association, intracellular lipid accumulation was assessed using the BODIPY 493/503 lipid droplet staining kit via confocal microscopy and flow cytometry. The results showed that collagen I stimulation significantly enhanced lipid droplet formation in ccRCC cells, indicating that collagen signaling may upregulate lipid synthesis capacity (Fig. S10C–E). Moreover, under collagen I stimulation, OSCAR overexpression further promoted lipid droplet accumulation, whereas OSCAR knockdown markedly reduced lipid storage (Fig. 4A, B and Fig. S10F–J).Consistent with these findings, triglyceride (TG) quantification assays demonstrated that collagen I increased intracellular TG content, which was further elevated by OSCAR overexpression and suppressed by OSCAR knockdown (Fig. 4C and Fig. S10K, L). In line with the in vitro results, OSCAR knockdown in a subcutaneous tumor model also significantly decreased TG levels in tumor tissues, further supporting its functional role in vivo (Fig. S10M). Together, these findings indicate that OSCAR serves as a critical regulator of lipid accumulation in ccRCC. The impact of the collagen I–OSCAR axis on lipid metabolism in ccRCC was investigated by examining the expression of genes related to lipid metabolic processes. qPCR analysis revealed that treatment with collagen I or overexpression of OSCAR significantly increased the mRNA levels of key fatty acid (FA) synthases FA synthase (FASN) and acetyl-CoA carboxylase alpha (ACC1), whereas OSCAR knockdown reduced their expression (S11A–D). Western blot analysis validated these observations, revealing parallel changes on the protein expression level (Fig. 4D, E;and Fig. S11E), indicating that collagen I–OSCAR primarily modulates ccRCC lipid metabolism by regulating lipid biosynthesis. Previous studies have indicated that the Hippo pathway is associated with lipid metabolism regulation [23, 33, 34]. To investigate the contribution of the Hippo pathway to collagen I–OSCAR–induced lipid accumulation, we examined dynamic expression patterns of Hippo signaling components and lipid metabolism–associated proteins after collagen I treatment.Experimental results suggested that the expression of key lipogenic enzymes FASN and ACC1 and YAP downstream effectors CTGF and CYR61 lagged behind phosphorylated YAP changes, supporting the idea that collagen I–mediated Hippo pathway control precedes lipid metabolism regulation (Fig. 4F). The collagen I–mediated upregulation of CTGF, FASN, and ACC1 in OSCAR-overexpressing cells was significantly mitigated by treatment with the YAP–TEAD inhibitor Verteporfin (Fig. 4G and Fig. S11F–G). BODIPY 493/503 lipid droplet staining and TG quantification demonstrated that Verteporfin inhibited OSCAR overexpression–induced lipid droplet formation and TG accumulation, indicating that collagen I–OSCAR regulates ccRCC lipid metabolism through modulation of the Hippo pathway (Fig. 4H and Fig. S12A–C). Previous studies have established that YAP regulates cellular lipid synthesis by modulating the key lipogenic transcription factor sterol regulatory element binding transcription factor 1 (SREBP1) [24, 35]. To further delineate the molecular mechanism linking collagen I–OSCAR signaling to lipid metabolic reprogramming, we investigated the interplay between YAP and SREBP1 in controlling the expression of lipogenic genes. In CAKI-1 cells, collagen I treatment markedly increased the levels of mature SREBP1 and its downstream targets FASN and ACC1. In contrast, OSCAR knockdown or pharmacological inhibition of YAP by verteporfin substantially attenuated these effects (Fig. S12D).Moreover, silencing of SREBP1 significantly reduced the expression of FASN and ACC1 (Fig. S12E), indicating that SREBP1 is required for collagen I–induced activation of lipogenic gene expression. Consistently, triglyceride quantification assays demonstrated that SREBP1 knockdown largely abolished collagen I–induced TG accumulation in CAKI-1 cells (Fig. S12F).

To better understand the function of OSCAR in lipid metabolism regulation in ccRCC, lipidomic profiling was conducted using CAKI-1 cells with OSCAR knockdown and control cells.Analysis showed that OSCAR knockdown induced an overall lowering of total lipid content, including a significant decrease in TG levels.In contrast, glycerophospholipids, particularly phosphatidylcholine (PC), were found to accumulate upon OSCAR depletion (Fig. 4J, K and Fig. S12G).A heatmap of differentially expressed lipid species (VIP > 1, *p* < 0.05) revealed that OSCAR knockdown substantially altered the expression landscape of numerous lipid molecules in ccRCC cells (Fig. 4L). Notably, nearly all triglyceride subtypes, varying in FA chain length and saturation, were affected (Fig. S12H).These results indicate that OSCAR is involved not only in lipid synthesis but also in the regulation of specific lipid subclass distribution and membrane lipid homeostasis. Consistent with these global alterations in lipid composition, further stratification of FA species by chain length revealed a selective remodeling pattern upon OSCAR knockdown. Specifically, the abundance of C14–16 and ≥C22 FA species was significantly reduced, whereas the C18–20 fraction remained largely unchanged in CAKI-1 cells (Fig. S12I). This selective shift in FA chain-length distribution supports a role for OSCAR in shaping lipid composition and metabolic balance, rather than uniformly regulating de novo FA synthesis.

To determine whether lipid availability contributes to OSCAR-dependent proliferation under collagen I stimulation, A498 and CAKI-1 cells were cultured in the presence of collagen I and subjected to OSCAR knockdown with or without oleic acid (OA) supplementation. Under collagen I stimulation, OSCAR depletion markedly suppressed cell proliferation in both cell lines, whereas OA treatment significantly restored the proliferative capacity of OSCAR-deficient cells (Fig. S12J, K). Notably, OA supplementation did not fully rescue proliferation to the level observed in control cells, indicating that lipid supplementation partially compensates for the loss of OSCAR signaling under collagen I stimulation.

### SAV1 is identified as a functional target of the collagen I–OSCAR axis in ccRCC

**Fig. 5:**
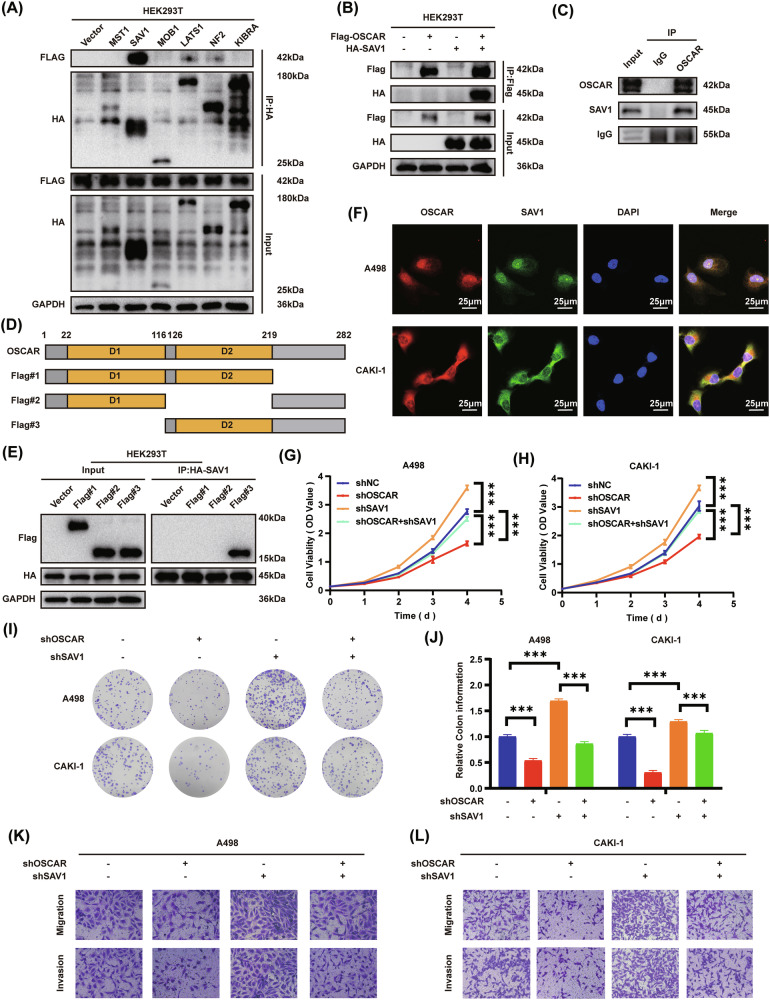
SAV1 is identified as a functional target of the Collagen I–OSCAR axis in ccRCC. **A** Co-IP and Western blot analysis of HEK293T cells pretreated with collagen I and co-transfected with Flag-OSCAR and HA-tagged Hippo pathway components. **B** Specific interaction between OSCAR and SAV1 verified by Co-IP in HEK293T cells pretreated with collagen I and co-transfected with Flag-OSCAR and HA-SAV1 plasmids. **C** Co-IP using HEK293T cells treated with collagen I without plasmid transfection. **D** Schematic diagram of full-length OSCAR (1–282 aa) and three truncation mutants. **E** HEK293T cells were first exposed to collagen I and then transfected with the specified truncated OSCAR constructs. Subsequent co-immunoprecipitation and Western blot analyses were performed to determine which OSCAR domain mediates interaction with SAV1. **F** Immunofluorescence analysis showing subcellular colocalization of OSCAR and SAV1 in collagen I-treated ccRCC cells. **G**, **H** CCK-8 assay depicting proliferation curves of selected ccRCC cell lines under different conditions (*n* = 3, two-way ANOVA with appropriate post-hoc tests). **I**, **J** Colony formation assay of selected ccRCC cell lines under different conditions (*n* = 3, one-way ANOVA followed by Dunnett’s post-hoc test). **K**, **L** Transwell migration and invasion assays assessing the motility of selected ccRCC cell lines under various treatments (*n* = 3). Data represent at least three independent experiments. Statistical significance: *P < 0.05, **P < 0.01, ***P < 0.001.

Most membrane-bound receptors, such as OSCAR, convey signals by forming complexes with specific intracellular proteins [36]. The Hippo signaling pathway, a representative multi-protein cascade, depends on dynamic interactions among its core scaffold proteins for proper activation [37]. Building on our previous findings that OSCAR modulates Hippo pathway regulation via the phosphorylation of LATS1/2, we further sought to identify its potential interacting partners. To further investigate potential OSCAR-interacting proteins, HA-tagged constructs of core Hippo pathway components known to regulate LATS1/2 activity were co-expressed with FLAG-tagged OSCAR in HEK293T cells [38], followed by co-IP under collagen I stimulation. The results revealed a strong physical interaction between OSCAR and SAV1, while weaker binding was observed with NF2 and LATS1, and no detectable interaction was observed with MST1, Kibra, or MOB1 (Fig. 5A).The direct interaction between FLAG-tagged OSCAR and HA-tagged SAV1 was further validated by bidirectional exogenous co-IP and endogenous co-IP assays (Fig. 5B, C). To determine which domains of OSCAR were necessary for this interaction, three truncation mutants of FLAG-tagged OSCAR were designed based on its predicted structure and transfected into HEK293T cells (Fig. 5D). The results showed that strong binding to SAV1 required the simultaneous presence of both the D2 extracellular domain and the cytoplasmic tail of OSCAR, indicating that both regions are indispensable for the interaction (Fig. 5E). Immunofluorescence microscopy further confirmed the colocalization of OSCAR and SAV1 in ccRCC cell lines (Fig. 5F). Collectively, these results indicate that the collagen I–OSCAR axis may influence ccRCC progression through its direct interaction with SAV1. To assess the biological relevance of SAV1 in mediating OSCAR-dependent ccRCC progression, rescue experiments were conducted by knockdown SAV1 in OSCAR-deficient ccRCC cells. SAV1 knockdown cell lines were established (Fig. S13A, B). CCK-8 and colony formation assays demonstrated that knockdown SAV1 effectively rescued the impaired proliferation induced by OSCAR knockdown (Fig. 5G–J). Similarly, transwell assays confirmed that the decreased migration and invasion capacity caused by OSCAR knockdown was reversed upon SAV1 depletion (Fig. 5K, L and Fig. S13C, D). In summary, the findings show that SAV1 mediates the effect of the collagen I–OSCAR axis on ccRCC cell behavior.

### Collagen I–OSCAR–SAV1 axis suppresses the Hippo pathway and drives ccRCC progression and lipid reprogramming

**Fig. 6:**
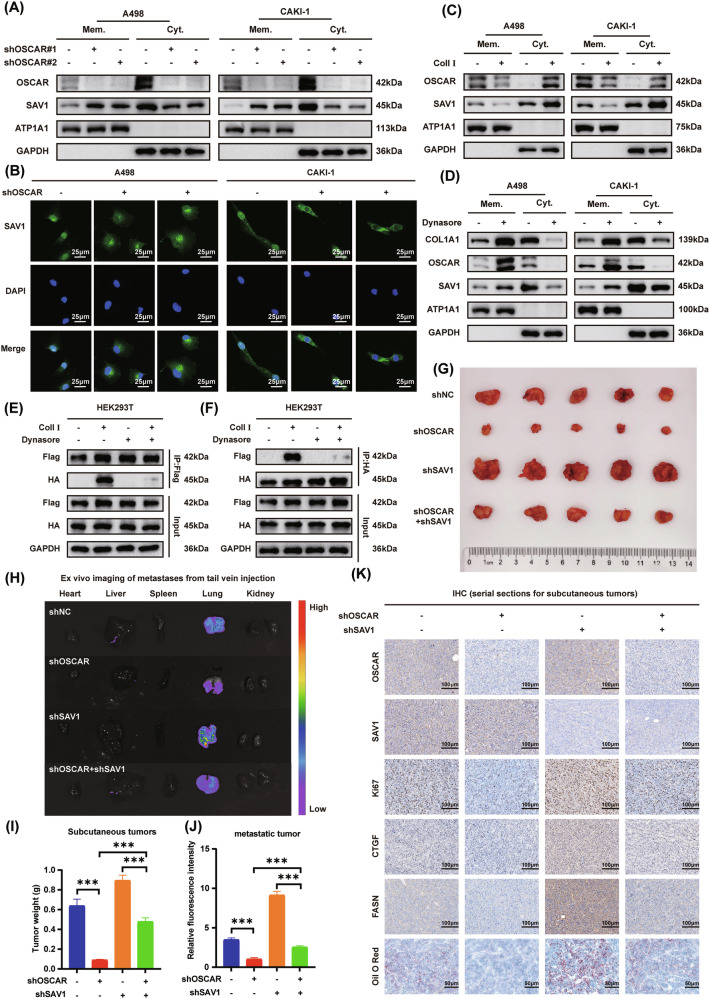
Collagen I–OSCAR–SAV1 axis suppresses the Hippo pathway and drives ccRCC progression and lipid reprogramming. **A** Western blot analysis was performed to compare the subcellular distribution of OSCAR and SAV1 between OSCAR-knockdown and control cells following collagen I treatment. **B** Immunofluorescence analysis demonstrating SAV1 distribution after OSCAR knockdown under collagen I stimulation. **C** Western blot comparing OSCAR and SAV1 localization between collagen-treated and untreated cells. **D** Effect of the endocytosis inhibitor Dynasore on the subcellular distribution of COL1A1, OSCAR, and SAV1. **E**, **F** Co-IP and Western blot analysis of HEK293T cells co-transfected with Flag-OSCAR and HA-SAV1 under indicated treatments. **G** Representative photographs of subcutaneous tumors collected from each treatment group (*n* = 5). **H** Bioluminescence imaging of major organs in metastatic models 8 weeks after intravenous A498 cell injection (*n* = 5). **I** Tumor weights from subcutaneous xenografts in each group (*n* = 5, one-way ANOVA followed by Dunnett’s post-hoc test). **J** Quantification of lung bioluminescence signal intensity in the metastatic model (*n* = 5, one-way ANOVA followed by Dunnett’s post-hoc test). **K** Representative IHC staining of OSCAR, SAV1, Ki67, CTGF, and FASN, and Oil Red O staining in subcutaneous tumors (*n* = 5). Data are presented as mean ± SD and represent at least three independent experiments. *P < 0.05, **P < 0.01, ***P < 0.001.

To elucidate the mechanism by which the collagen I–OSCAR axis regulates ccRCC progression through interaction with SAV1, we conducted a series of mechanistic investigations. qPCR and Western blot analyses showed that neither knockdown nor overexpression of OSCAR affected the mRNA or total protein levels of SAV1, suggesting that its regulation may occur at the level of localization or function (Fig. S14A–D). Previous studies have reported that SAV1 promotes LATS1/2 activation by translocating from the cytoplasm to the plasma membrane [39]. Therefore, we performed membrane–cytosol fractionation experiments and found that OSCAR knockdown enhanced SAV1 localization at the membrane (Fig. 6A). This was further confirmed by immunofluorescence staining, which also showed increased membrane-associated SAV1 upon OSCAR knockdown (Fig. 6B).Our prior domain-mapping experiments demonstrated that interaction between OSCAR and SAV1 requires both the D2 extracellular domain and intracellular tail of OSCAR. However, the D1/D2 domains of OSCAR are located extracellularly, while SAV1 is a cytoplasmic protein, and thus theoretically, SAV1 should not interact directly with the D2 domain of OSCAR. Given that the D2 domain of OSCAR has been reported to bind collagen I, we hypothesized that the interaction between OSCAR and SAV1 may be facilitated by collagen binding [40]. To test this, we examined the subcellular localization of OSCAR and SAV1 under collagen I stimulation. Without collagen I, OSCAR primarily localized to the membrane; however, following collagen I treatment, both OSCAR and SAV1 showed reduced membrane localization and increased cytoplasmic presence (Fig. 6C), indicating typical receptor endocytosis behavior [41]. Subsequently, ccRCC cells were treated with the endocytosis inhibitor dynasore in the presence of collagen I. Inhibition of endocytosis prevented the internalization of OSCAR and reduced cytoplasmic collagen accumulation, while concurrently increasing SAV1 membrane localization (Fig. 6D). Finally, co-IP assays revealed that OSCAR and SAV1 exhibited significant binding only under conditions of collagen I stimulation and without dynasore treatment. No interaction was observed without collagen or in the presence of dynasore (Fig. 6E, F). These findings collectively demonstrate that following engagement with collagen I through its D2 domain, OSCAR undergoes receptor-mediated endocytosis and translocates to the cytoplasm, where it interacts with SAV1. This interaction prevents SAV1 membrane localization, thereby suppressing activation of the Hippo pathway.

Next, the in vivo function of the collagen I–OSCAR–SAV1 axis was investigated using animal models. Subcutaneous tumor assays demonstrated that SAV1 knockdown reversed the growth inhibition induced by OSCAR depletion, while bioluminescence imaging of metastatic tumors showed that SAV1 knockdown rescued the suppression of metastasis caused by OSCAR knockdown (Fig. 6G–J and Fig. S14E). IHC and Oil Red O staining of subcutaneous tumors revealed that SAV1 knockdown restored the reduced expression of Ki67 and CTGF in OSCAR-deficient tumors, as well as rescued the decrease in FASN expression and lipid droplet accumulation induced by OSCAR knockdown (Fig. 6K). Furthermore, H&E analysis of lungs from the metastasis model indicated that SAV1 depletion increased both the number and size of pulmonary metastatic nodules in OSCAR-knockdown mice (Fig. S14F). To determine whether OSCAR knockdown suppresses tumor growth through Hippo pathway reactivation in vivo, we examined YAP phosphorylation and SAV1 localization in subcutaneous xenograft tumors. Western blot analysis showed that OSCAR depletion markedly increased YAP phosphorylation without affecting total YAP levels, whereas concurrent knockdown of SAV1 largely reversed YAP phosphorylation induced by OSCAR depletion (Fig. S14G).Consistently, subcellular fractionation of tumor tissues revealed that OSCAR knockdown promoted the membrane localization of SAV1. This effect was abolished upon SAV1 knockdown, confirming the requirement of SAV1 in mediating OSCAR-dependent Hippo reactivation in vivo (Fig. S14H). Collectively, these findings indicate that the collagen I–OSCAR–SAV1 axis promotes ccRCC progression and lipid accumulation through modulation of the Hippo pathway.

### CCP-LNP targeting the collagen I–OSCAR axis suppresses ccRCC progression

**Fig. 7:**
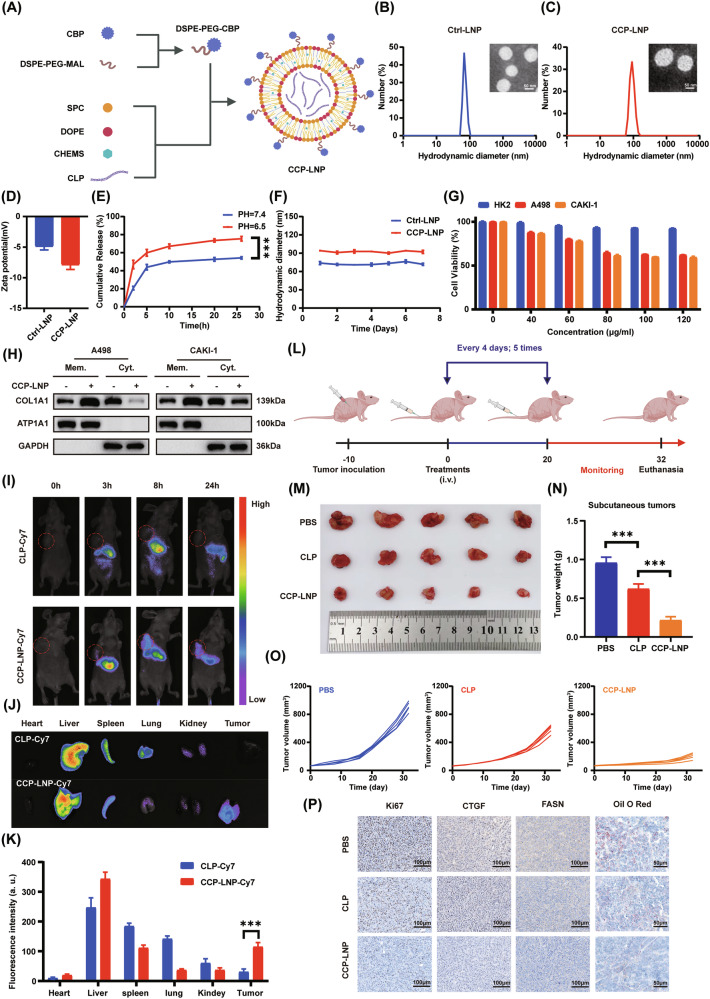
CCP-LNP targeting the Collagen I–OSCAR axis suppresses ccRCC progression. **A** Schematic representation of CCP-LNP composition. **B**, **C** DLS and TEM analyses showing particle size and morphology of Ctrl-LNP and CCP-LNP with representative images. **D** Zeta potential of Ctrl-LNP and CCP-LNP. **E** In vitro release profile of CLP-Cy7 from CCP-LNP in PBS at different pH conditions. **F** Time-dependent changes in particle size of Ctrl-LNP and CCP-LNP in 50% fetal bovine serum (FBS). **G** CCK-8 assay evaluating cytotoxicity of CCP-LNP at various concentrations (*n* = 3). **H** Western blot analysis of COL1A1 subcellular localization in collagen I-treated cells with or without CCP-LNP. **I–K** In vivo imaging of organ distribution and time-dependent biodistribution after tail vein injection of CLP-Cy7 or CCP-LNP-Cy7 in nude mice (*n* = 3; t-test). **L** Schematic of CCP-LNP treatment protocol in a subcutaneous tumor model. **M**, **N** Representative images of excised tumors and corresponding weights in each treatment group (*n* = 5; one-way ANOVA followed by Dunnett’s post-hoc test). **O** Tumor growth curves measured every four days until day 32 (*n* = 5). **P** Representative IHC staining for Ki67, CTGF, and FASN, and Oil Red O staining of subcutaneous tumors (*n* = 5). All data are shown as mean ± SD from at least three independent experiments. *P < 0.05; **P < 0.01; ***P < 0.001.

Given that the collagen I–OSCAR–SAV1 axis promotes ccRCC proliferation, pharmacological blockade of this pathway may offer a promising therapeutic approach. Previous studies reported that the CLP peptide competitively binds to OSCAR and inhibits its interaction with collagen I [40]. Based on this, we hypothesized that CLP peptide could serve as a candidate inhibitor to block the collagen I–OSCAR interaction and thereby exert antitumor effects in ccRCC. To achieve targeted delivery and enhance peptide stability, we engineered a lipid nanoparticle delivery system named CCP-LNP (Fig. 7A). The surface-conjugated collagen-binding peptide (CBP) and the enhanced permeability and retention (EPR) effect enable CCP-LNP to accumulate within the collagen-rich TME. [42–44]. Moreover, the pH-responsive properties provided by DOPE and CHEMS enable regulated release of the encapsulated CLP peptide in response to the acidic TME [45]. The successful fabrication of CCP-LNP was verified through transmission electron microscopy (TEM), dynamic light scattering (DLS), and zeta potential measurements, revealing an average particle size of approximately 90 nm and a slightly negative surface charge. (Fig. 7B–D). Drug release kinetics of the liposomes were evaluated in PBS under varying pH conditions with constant agitation at 37 °C. Analysis revealed that the release efficiency of CLP exhibited markedly greater release at pH 6.5 compared with pH 7.4, indicating enhanced release under the mildly acidic conditions mimicking the TME. (Fig. 7E). Moreover, incubation in 50% FBS solution for 7 days showed minimal changes in particle size, suggesting good biological stability of CCP-LNP (Fig. 7F). Functionally, CCK-8 assays showed that CCP-LNP markedly suppressed the proliferation of ccRCC cells induced by collagen I, while exerting minimal effects on normal HK2 cells. Notably, increasing the concentration beyond 80 μg/mL did not further enhance its inhibitory effect, and thus 80 μg/mL was selected for subsequent in vitro experiments (Fig. 7G). Western blot analysis further confirmed that CCP-LNP restored the activity of the Hippo pathway suppressed by collagen I, as evidenced by elevated YAP phosphorylation, and concurrently reduced collagen I-mediated endocytosis (Fig. 7H and Fig. S15A).

To assess the tumor-targeting capability of CCP-LNP, Cy7-labeled CLP peptide and CCP-LNP nanoparticles were administered via intravenous injection in nude mice, and subsequent in vivo fluorescence imaging showed that CCP-LNP-Cy7 accumulated in tumors to a much greater extent than free CLP-Cy7. (Fig. 7I–K). To further evaluate the in vivo biodistribution and stability of CCP-LNP beyond the initial accumulation phase, longitudinal fluorescence imaging was performed at 6, 24, 48, and 72 h post-injection. Tumor-associated fluorescence signals were detectable as early as 6 h, peaked at 24 h, and remained readily observable at 48 and 72 h, indicating sustained tumor retention and favorable in vivo stability of CCP-LNP beyond 24 h (Fig. S15B).To evaluate its therapeutic efficacy, a subcutaneous tumor model was established (Fig. 7L), in which CCP-LNP treatment significantly suppressed tumor growth, as indicated by tumor weight and growth curves (Fig. 7M, N), without observable weight loss during treatment (Fig. S15C).To assess the potential adverse effects of CCP-LNP treatment on normal tissues, various blood parameters were analyzed post-treatment, and no significant differences were observed compared to the control group. Histopathological examination by H&E staining revealed no obvious pathological alterations in major organs. Importantly, no detectable histological abnormalities were observed in collagen-rich tissues, including skin and knee joints, following CCP-LNP administration (Fig. S15D, E). IHC as well as Oil Red O analysis of tumor tissues indicated that CCP-LNP treatment led to marked reductions in Ki67, CTGF, FASN expression and lipid droplet accumulation (Fig. 7P).These results demonstrate that CCP-LNP can markedly inhibit tumor progression, proposing a novel strategy for the development of innovative therapies against ccRCC. To exclude potential non-specific antitumor effects of the nanoparticle carrier, tumor-bearing mice were treated with Ctrl-LNP. Neither gross tumor morphology nor tumor weight differed significantly between PBS- and Ctrl-LNP-treated groups (Fig. S15F, G), indicating that the liposomal carrier itself does not exert detectable antitumor activity in vivo.

## Discussion

**Fig. 8:**
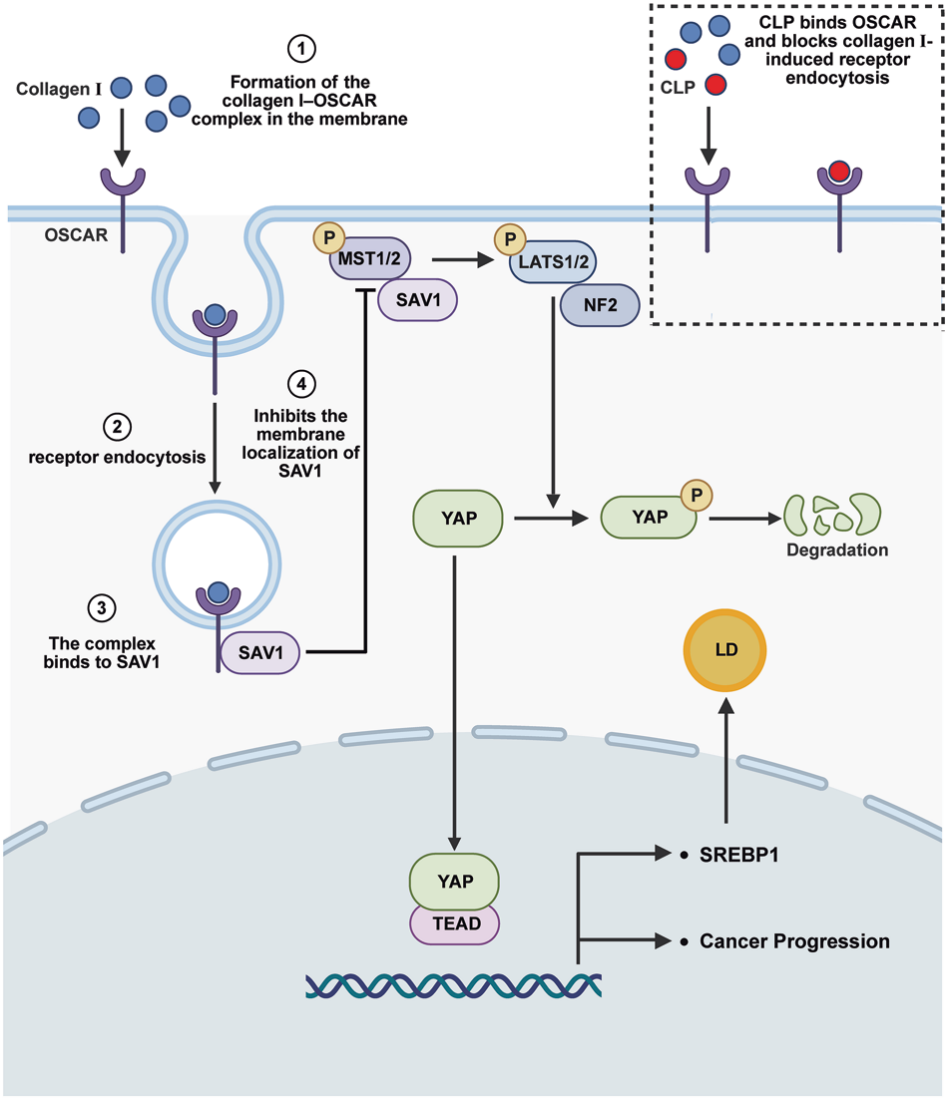
Upon collagen I binding, OSCAR undergoes endocytosis and sequesters SAV1, preventing its association with MST1/2 at the plasma membrane. This disrupts Hippo signaling, leading to reduced phosphorylation of LATS1/2 and YAP. Consequently, dephosphorylated YAP translocates into the nucleus, activating transcription of CYR61, CTGF, FASN, and ACC1, which drive ccRCC progression and lipid metabolic reprogramming. CLP acts as a competitive ligand for OSCAR, blocking its internalization and interaction with SAV1.

Changes in elements of the TME are strongly correlated with the onset and metastatic progression of various cancers [46]. Collagen I, the major fibrous component of the ECM, provides essential structural support for tissues while simultaneously facilitating the activation of several oncogenic signaling pathways in solid tumors, including breast, liver, and pancreatic cancers [8, 10, 47]. In this study, we demonstrate that collagen I does not primarily exert its effects in ccRCC through classical receptors, such as DDR1 or integrins, but instead promotes tumor progression via OSCAR. Furthermore, we show that the interaction between collagen I and OSCAR disrupts the membrane localization of SAV1, leading to decreased phosphorylation of LATS1/2, enhanced transcriptional activity of YAP, and consequently increased tumor cell proliferation and lipid accumulation. Notably, the engineered CCP-LNP that we developed competitively binds to OSCAR, effectively blocking the interaction between collagen I and OSCAR, restoring Hippo pathway activity, and markedly suppressing ccRCC progression in cultured cells and animal models (Fig. 8).

OSCAR was named for its expression in both pre-osteoclasts and mature osteoclasts in mice [14]. Subsequent research has shown that OSCAR expression extends beyond bone marrow–derived cells, being also present in non-hematopoietic cell types such as chondrocytes. In inducible osteoarthritis (OA) models, genetic deletion of OSCAR significantly reduced multiple pathological features of OA, supporting its functional involvement in cartilage degradation [17]. However, the biological roles of OSCAR beyond bone homeostasis remain largely unexplored.In this study, the function of OSCAR is extended to the field of oncology. Specifically, OSCAR mediates the influence on the Hippo signaling pathway by collagen I in ccRCC, further highlighting its biological significance. Our study does not deny the potential roles of other collagen I receptors in ccRCC. Rather, among the receptors examined and under the experimental conditions defined in this study, OSCAR represents the primary receptor mediating collagen I-associated biological phenotypes. Moreover, the biological effects of collagen I on tumor cells may be context-dependent. For example, collagen I synthesized by tumor cells themselves may influence tumor behavior through mechanisms distinct from those mediated by microenvironment-derived collagen I [9]. Given the established expression of OSCAR in human immune cells, including macrophages, neutrophils, and dendritic cells, and consistent with previous immune-stromal profiling in ccRCC, it is conceivable that OSCAR may exert context-dependent functions in the TME [20]. While the present study focuses on the tumor-intrinsic role of OSCAR in mediating collagen I-Hippo signaling and metabolic reprogramming, further investigations will be required to delineate its specific functions in immune cell subsets within the ccRCC microenvironment.

Previous investigations have revealed that collagen I can modulate the Hippo signaling pathway via multiple mechanisms [10, 29]. SAV1, a core scaffold protein in the Hippo pathway, is known to inhibit tumor progression in several cancer types [26, 48]. Recent studies reveal that SAV1 can recruit MST1/2 to the plasma membrane, thereby activating the pathway through parallel routes [39]. Therefore, controlling the membrane localization of SAV1 is regarded as a key step in initiating Hippo pathway activation [49]. We identify a novel mechanism linking collagen I to Hippo signaling. Collagen I binds to the membrane receptor OSCAR, disrupting SAV1 localization and activating downstream transcriptional programs. This pathway drives tumor progression and lipid reprogramming in ccRCC.

The buildup of LDs is a characteristic histological hallmark of ccRCC and is crucial for its onset and progression [50]. Aberrant lipid storage has been closely associated with high tumor aggressiveness, poor prognosis, and therapeutic resistance [51, 52]. Several metabolic processes, such as de novo FA synthesis, FA uptake, and reduced fatty acid oxidation (FAO), have been linked to the promotion of intracellular lipid buildup in tumor cells [53–55]. In obese individuals, collagen is heavily deposited in adipose tissue and has been found to influence adipocyte migration, proliferation, and differentiation via signaling pathways including Hippo–YAP and NF-κB [56–58]. Nevertheless, how collagen contributes mechanistically to tumor lipid metabolism remains largely unexplored. In our study, we observed that the addition of collagen I markedly increased LDs accumulation. This finding prompted us to further investigate the role of the collagen I–OSCAR axis in regulating Hippo signaling, particularly its connection with lipid metabolism. Our results preliminarily indicate that the effect of collagen I–OSCAR on lipid accumulation is mediated through Hippo signaling. Importantly, our data support an indirect mechanism whereby YAP activation drives lipogenic reprogramming through transcriptional regulators such as SREBP1, rather than through direct transcriptional control of canonical lipogenic genes. This discovery not only delineates a specific pathway by which collagen I modulates FA metabolism in tumors but also deepens our understanding of the interplay between the Hippo pathway and lipid metabolic reprogramming. Nevertheless, this study has certain limitations. Previous reports suggest that lipid metabolic reprogramming can reciprocally influence Hippo and other tumor-related pathways [33]. In our work, we only partially validated the impact of Hippo signaling on lipid accumulation through functional rescue experiments. The precise molecular mechanisms, potential targets, and the existence of possible feedback regulation remain to be further elucidated.

Currently, the primary therapeutic approaches for ccRCC include molecular agents targeting the mammalian target of rapamycin (mTOR), Hypoxia-Inducible Factor 2 Alpha (HIF-2α), and vascular endothelial growth factor (VEGF) pathways, as well as immune checkpoint inhibitors (ICIs) [59, 60]. While these approaches have extended patient survival, resistance to therapy is common and eventually limits their effectiveness [59, 61]. This therapeutic challenge has driven the search for new treatment options, among which YAP inhibition has gained attention. Several YAP-targeting compounds have already reached clinical application, and in some cancers, they have shown more favorable prognostic outcomes than conventional kinase inhibitors such as dasatinib [62]. In this study, we developed CCP-LNP based on a LNP delivery system, which specifically blocks the interaction between collagen I and OSCAR, effectively restoring the tumor-suppressive activity of the Hippo pathway. This formulation demonstrated pronounced inhibitory effects on tumor growth in both cell culture and animal models, while exhibiting low biological toxicity and favorable safety profiles. In subsequent studies, we plan to combine CCP-LNP with mTOR inhibitors, HIF-2α inhibitors, EGF inhibitors, or ICIs to evaluate their combined therapeutic efficacy and potential drug-related adverse effects.

In conclusion, Our findings demonstrate that collagen I–OSCAR signaling promotes the progression of ccRCC by restricting SAV1 to the cytoplasm, thereby activating YAP as an oncogenic driver. In ccRCC tissues, elevated expression of collagen I and OSCAR is associated with reduced survival outcomes. Based on these observations, we developed CCP-LNP, which effectively disrupts the collagen I–OSCAR interaction and significantly suppresses ccRCC progression, offering a novel therapeutic strategy for this malignancy.

## Supplementary information

Supplemental file

Original uncropped images of all blots

## Data Availability

Data generated in the present study are available from the corresponding author upon request. The sequencing and lipidomics have been deposited in Science Data Bank. (https://www.scidb.cn/, 10.57760/sciencedb.34204).

## References

[1] Jonasch E, Walker CL, Rathmell WK. Clear cell renal cell carcinoma ontogeny and mechanisms of lethality. Nat Rev Nephrol. 2021;17:245–61. 33144689 10.1038/s41581-020-00359-2 PMC8172121

[2] Sung H, Ferlay J, Siegel RL, Laversanne M, Soerjomataram I, Jemal A, et al. Global Cancer Statistics 2020: GLOBOCAN estimates of incidence and mortality worldwide for 36 cancers in 185 countries. CA Cancer J Clin. 2021;71:209–49. 33538338 10.3322/caac.21660

[3] Kuczek DE, Larsen AMH, Thorseth ML, Carretta M, Kalvisa A, Siersbæk MS, et al. Collagen density regulates the activity of tumor-infiltrating T cells. J Immunother Cancer. 2019;7:68. 30867051 10.1186/s40425-019-0556-6 PMC6417085

[4] Nissen NI, Karsdal M, Willumsen N. Collagens and cancer associated fibroblasts in the reactive stroma and its relation to cancer biology. J Exp Clin Cancer Res. 2019;38:115. 30841909 10.1186/s13046-019-1110-6 PMC6404286

[5] Peng DH, Rodriguez BL, Diao L, Chen L, Wang J, Byers LA, et al. Collagen promotes anti-PD-1/PD-L1 resistance in cancer through LAIR1-dependent CD8(+) T cell exhaustion. Nat Commun. 2020;11:4520. 32908154 10.1038/s41467-020-18298-8 PMC7481212

[6] Mollenhauer J, Roether I, Kern HF. Distribution of extracellular matrix proteins in pancreatic ductal adenocarcinoma and its influence on tumor cell proliferation in vitro. Pancreas. 1987;2:14–24. 3554225 10.1097/00006676-198701000-00003

[7] Tian C, Clauser KR, Öhlund D, Rickelt S, Huang Y, Gupta M, et al. Proteomic analyses of ECM during pancreatic ductal adenocarcinoma progression reveal different contributions by tumor and stromal cells. Proc Natl Acad Sci USA. 2019;116:19609–18. 31484774 10.1073/pnas.1908626116 PMC6765243

[8] Chen Y, Kim J, Yang S, Wang H, Wu CJ, Sugimoto H, et al. Type I collagen deletion in αSMA(+) myofibroblasts augments immune suppression and accelerates progression of pancreatic cancer. Cancer Cell. 2021;39:548–565. 33667385 10.1016/j.ccell.2021.02.007 PMC8423173

[9] Chen Y, Yang S, Tavormina J, Tampe D, Zeisberg M, Wang H, et al. Oncogenic collagen I homotrimers from cancer cells bind to α3β1 integrin and impact tumor microbiome and immunity to promote pancreatic cancer. Cancer Cell. 2022;40:818–34. 35868307 10.1016/j.ccell.2022.06.011 PMC9831277

[10] Xiong YX, Zhang XC, Zhu JH, Zhang YX, Pan YL, Wu Y, et al. Collagen I-DDR1 signaling promotes hepatocellular carcinoma cell stemness via Hippo signaling repression. Cell Death Differ. 2023;30:1648–65. 37117273 10.1038/s41418-023-01166-5 PMC10307904

[11] Zhao H, Wang E. COL1A1 drives tumor progression in kidney renal clear cell carcinoma by regulating EMT through the PI3K/AKT pathway. Cancer Cell Int. 2025;25:314. 40851082 10.1186/s12935-025-03956-y PMC12376327

[12] Ji X, Yun Y, Zhu Z, Wu T, Ruan M, Fan Y, et al. COL1A1 promotes clear cell renal cell carcinoma progression through the WNT/β-catenin signaling pathway. Cell Signal. 2026;138:112259. 41270811 10.1016/j.cellsig.2025.112259

[13] Cen J, Liang Y, Feng Z, Chen X, Chen J, Wang Y, et al. HSA_CIRC_0057105 modulates a balance of epithelial-mesenchymal transition and ferroptosis vulnerability in renal cell carcinoma. Clin Transl Med. 2023;13:e1339. 37496319 10.1002/ctm2.1339 PMC10372385

[14] Kim N, Takami M, Rho J, Josien R, Choi Y. A novel member of the leukocyte receptor complex regulates osteoclast differentiation. J Exp Med. 2002;195:201–9. 11805147 10.1084/jem.20011681 PMC2193610

[15] Maruotti N, Grano M, Colucci S, d’Onofrio F, Cantatore FP. Osteoclastogenesis and arthritis. Clin Exp Med. 2011;11:137–45. 21069419 10.1007/s10238-010-0117-2

[16] Barrow AD, Raynal N, Andersen TL, Slatter DA, Bihan D, Pugh N, et al. OSCAR is a collagen receptor that costimulates osteoclastogenesis in DAP12-deficient humans and mice. J Clin Invest. 2011;121:3505–16. 21841309 10.1172/JCI45913 PMC3163954

[17] Park DR, Kim J, Kim GM, Lee H, Kim M, Hwang D, et al. Osteoclast-associated receptor blockade prevents articular cartilage destruction via chondrocyte apoptosis regulation. Nat Commun. 2020;11:4343. 32859940 10.1038/s41467-020-18208-y PMC7455568

[18] Zou W, Teitelbaum SL. Absence of Dap12 and the αvβ3 integrin causes severe osteopetrosis. J Cell Biol. 2015;208:125–36. 25547154 10.1083/jcb.201410123 PMC4284236

[19] Liao X, Bu Y, Zhang Y, Xu B, Liang J, Jia Q, et al. OSCAR facilitates malignancy with enhanced metastasis correlating to inhibitory immune microenvironment in multiple cancer types. J Cancer. 2021;12:3769–80. 34093786 10.7150/jca.51964 PMC8176254

[20] Lyu F, Zhong Y, He Q, Xiao W, Zhang X. Identification and validation of prognostic biomarkers in ccRCC: immune-stromal score and survival prediction. BMC Cancer. 2025;25:148. 39871215 10.1186/s12885-025-13534-0 PMC11771106

[21] Dey A, Varelas X, Guan KL. Targeting the Hippo pathway in cancer, fibrosis, wound healing and regenerative medicine. Nat Rev Drug Discov. 2020;19:480–94. 32555376 10.1038/s41573-020-0070-z PMC7880238

[22] Yu FX, Guan KL. The Hippo pathway: regulators and regulations. Genes Dev. 2013;27:355–71. 23431053 10.1101/gad.210773.112 PMC3589553

[23] Miao D, Wang Q, Shi J, Lv Q, Tan D, Zhao C, et al. N6-methyladenosine-modified DBT alleviates lipid accumulation and inhibits tumor progression in clear cell renal cell carcinoma through the ANXA2/YAP axis-regulated Hippo pathway. Cancer Commun. 2023;43:480–502. 10.1002/cac2.12413 PMC10091108 36860124

[24] Ren Y, Chen J, Zhan X, Sheng S, Zhong Y, Gu M, et al. Liquid-liquid phase separation of GPS2-LATS1 promotes colorectal cancer progression by reprogramming lipid metabolism. Oncogene. 2025;44:3741–54. 40715488 10.1038/s41388-025-03498-7

[25] Matsuura K, Nakada C, Mashio M, Narimatsu T, Yoshimoto T, Tanigawa M, et al. Downregulation of SAV1 plays a role in pathogenesis of high-grade clear cell renal cell carcinoma. BMC Cancer. 2011;11:523. 22185343 10.1186/1471-2407-11-523 PMC3292516

[26] de Amorim ÍSS, de Sousa Rodrigues MM, Mencalha AL. The tumor suppressor role of salvador family WW domain-containing protein 1 (SAV1): one of the key pieces of the tumor puzzle. J Cancer Res Clin Oncol. 2021;147:1287–97. 33580421 10.1007/s00432-021-03552-3 PMC11801967

[27] Ricard-Blum S. The collagen family. Cold Spring Harb Perspect Biol. 2011;3:a004978. 21421911 10.1101/cshperspect.a004978 PMC3003457

[28] Su H, Karin M. Multifaceted collagen-DDR1 signaling in cancer. Trends Cell Biol. 2024;34:406–15. 37709651 10.1016/j.tcb.2023.08.003 PMC10927612

[29] Liu J, Wang J, Liu Y, Xie SA, Zhang J, Zhao C, et al. Liquid-liquid phase separation of DDR1 counteracts the hippo pathway to orchestrate arterial stiffening. Circ Res. 2023;132:87–105. 36475898 10.1161/CIRCRESAHA.122.322113

[30] Cox TR. The matrix in cancer. Nat Rev Cancer. 2021;21:217–38. 33589810 10.1038/s41568-020-00329-7

[31] Walther TC, Farese RV Jr. Lipid droplets and cellular lipid metabolism. Annu Rev Biochem. 2012;81:687–714. 22524315 10.1146/annurev-biochem-061009-102430 PMC3767414

[32] Shi J, Lv Q, Miao D, Xiong Z, Wei Z, Wu S, et al. HIF2α promotes cancer metastasis through TCF7L2-dependent fatty acid synthesis in ccRCC. Research. 2024;7:0322. 38390305 10.34133/research.0322 PMC10882601

[33] Koo JH, Guan KL. Interplay between YAP/TAZ and metabolism. Cell Metab. 2018;28:196–206. 30089241 10.1016/j.cmet.2018.07.010

[34] Ibar C, Irvine KD. Integration of hippo-YAP signaling with metabolism. Dev Cell. 2020;54:256–67. 32693058 10.1016/j.devcel.2020.06.025 PMC7373816

[35] Vaidyanathan S, Salmi TM, Sathiqu RM, McConville MJ, Cox AG, Brown KK. YAP regulates an SGK1/mTORC1/SREBP-dependent lipogenic program to support proliferation and tissue growth. Dev Cell. 2022;57:719–31. 35216681 10.1016/j.devcel.2022.02.004

[36] Wootten D, Christopoulos A, Marti-Solano M, Babu MM, Sexton PM. Mechanisms of signalling and biased agonism in G protein-coupled receptors. Nat Rev Mol Cell Biol. 2018;19:638–53. 30104700 10.1038/s41580-018-0049-3

[37] Fu M, Hu Y, Lan T, Guan KL, Luo T, Luo M. The Hippo signalling pathway and its implications in human health and diseases. Signal Transduct Target Ther. 2022;7:376. 36347846 10.1038/s41392-022-01191-9 PMC9643504

[38] Misra JR, Irvine KD. The hippo signaling network and its biological functions. Annu Rev Genet. 2018;52:65–87. 30183404 10.1146/annurev-genet-120417-031621 PMC6322405

[39] Yin F, Yu J, Zheng Y, Chen Q, Zhang N, Pan D. Spatial organization of Hippo signaling at the plasma membrane mediated by the tumor suppressor Merlin/NF2. Cell. 2013;154:1342–55. 24012335 10.1016/j.cell.2013.08.025 PMC3835333

[40] Haywood J, Qi J, Chen CC, Lu G, Liu Y, Yan J, et al. Structural basis of collagen recognition by human osteoclast-associated receptor and design of osteoclastogenesis inhibitors. Proc Natl Acad Sci USA. 2016;113:1038–43. 26744311 10.1073/pnas.1522572113 PMC4743793

[41] von Zastrow M, Sorkin A. Mechanisms for regulating and organizing receptor signaling by endocytosis. Annu Rev Biochem. 2021;90:709–37. 33606955 10.1146/annurev-biochem-081820-092427 PMC8608402

[42] Li Z, Zhang Y, Li M, Li J. Two-Step targeting-tunable semiconducting nanoswitches amplify mitochondrion damage and PD-L1 blockade for orthotopic pancreatic cancer therapy. Adv Funct Mater. 2025;35:2413233.

[43] Fang J, Islam W, Maeda H. Exploiting the dynamics of the EPR effect and strategies to improve the therapeutic effects of nanomedicines by using EPR effect enhancers. Adv Drug Deliv Rev. 2020;157:142–60. 32553783 10.1016/j.addr.2020.06.005

[44] Ma Z, Zeng P, Zhai T, Zhao Y, Liang H. In situ mitochondrial biomineralization for drug-free cancer therapy. Adv Mater. 2024;36:e2310218. 38315577 10.1002/adma.202310218

[45] Xia Y, Fang M, Dong J, Xu C, Liao Z, Ning P, et al. pH sensitive liposomes delivering tariquidar and doxorubicin to overcome multidrug resistance of resistant ovarian cancer cells. Colloids Surf B Biointerfaces. 2018;170:514–20. 29960952 10.1016/j.colsurfb.2018.06.055

[46] Huang J, Zhang L, Wan D, Zhou L, Zheng S, Lin S, et al. Extracellular matrix and its therapeutic potential for cancer treatment. Signal Transduct Target Ther. 2021;6:153. 33888679 10.1038/s41392-021-00544-0 PMC8062524

[47] Maller O, Drain AP, Barrett AS, Borgquist S, Ruffell B, Zakharevich I, et al. Tumour-associated macrophages drive stromal cell-dependent collagen crosslinking and stiffening to promote breast cancer aggression. Nat Mater. 2021;20:548–59. 33257795 10.1038/s41563-020-00849-5 PMC8005404

[48] Donninger H, Allen N, Henson A, Pogue J, Williams A, Gordon L, et al. Salvador protein is a tumor suppressor effector of RASSF1A with hippo pathway-independent functions. J Biol Chem. 2011;286:18483–91. 21489991 10.1074/jbc.M110.214874 PMC3099665

[49] Paul A, Annunziato S, Lu B, Sun T, Evrova O, Planas-Paz L, et al. Cell adhesion molecule KIRREL1 is a feedback regulator of Hippo signaling recruiting SAV1 to cell-cell contact sites. Nat Commun. 2022;13:930. 35177623 10.1038/s41467-022-28567-3 PMC8854406

[50] Tan SK, Hougen HY, Merchan JR, Gonzalgo ML, Welford SM. Fatty acid metabolism reprogramming in ccRCC: mechanisms and potential targets. Nat Rev Urol. 2023;20:48–60. 36192502 10.1038/s41585-022-00654-6 PMC10826284

[51] Du Y, Wang Q, Zhang X, Wang X, Qin C, Sheng Z, et al. Lysophosphatidylcholine acyltransferase 1 upregulation and concomitant phospholipid alterations in clear cell renal cell carcinoma. J Exp Clin Cancer Res. 2017;36:66. 28494778 10.1186/s13046-017-0525-1 PMC5427523

[52] Wettersten HI, Aboud OA, Lara PN Jr., Weiss RH. Metabolic reprogramming in clear cell renal cell carcinoma. Nat Rev Nephrol. 2017;13:410–9. 28480903 10.1038/nrneph.2017.59

[53] Ookhtens M, Kannan R, Lyon I, Baker N. Liver and adipose tissue contributions to newly formed fatty acids in an ascites tumor. Am J Physiol. 1984;247:R146–153. 6742224 10.1152/ajpregu.1984.247.1.R146

[54] Tan SK, Mahmud I, Fontanesi F, Puchowicz M, Neumann CKA, Griswold AJ, et al. Obesity-dependent adipokine chemerin suppresses fatty acid oxidation to confer ferroptosis resistance. Cancer Discov. 2021;11:2072–93. 33757970 10.1158/2159-8290.CD-20-1453 PMC8338847

[55] Shi J, Miao D, Lv Q, Wang K, Wang Q, Liang H, et al. The m6A modification-mediated OGDHL exerts a tumor suppressor role in ccRCC by downregulating FASN to inhibit lipid synthesis and ERK signaling. Cell Death Dis. 2023;14:560. 37626050 10.1038/s41419-023-06090-7 PMC10457380

[56] Al Hasan M, Martin PE, Shu X, Patterson S, Bartholomew C Type III collagen is required for adipogenesis and actin stress fibre formation in 3T3-L1 preadipocytes. Biomolecules. 2021;11:156. 10.3390/biom11020156 PMC7911635 33504048

[57] Liu X, Xu Q, Liu W, Yao G, Zhao Y, Xu F, et al. Enhanced migration of murine fibroblast-like 3T3-L1 preadipocytes on type I collagen-coated dish is reversed by silibinin treatment. Mol Cell Biochem. 2018;441:35–62. 28933025 10.1007/s11010-017-3173-z

[58] Xu Q, Liu X, Liu W, Hayashi T, Yamato M, Fujisaki H, et al. Type I collagen-induced YAP nuclear expression promotes primary cilia growth and contributes to cell migration in confluent mouse embryo fibroblast 3T3-L1 cells. Mol Cell Biochem. 2019;450:87–96. 29846859 10.1007/s11010-018-3375-z

[59] Young M, Jackson-Spence F, Beltran L, Day E, Suarez C, Bex A, et al. Renal cell carcinoma. Lancet. 2024;404:476–91. 39033764 10.1016/S0140-6736(24)00917-6

[60] Miao D, Margolis CA, Gao W, Voss MH, Li W, Martini DJ, et al. Genomic correlates of response to immune checkpoint therapies in clear cell renal cell carcinoma. Science. 2018;359:801–6. 29301960 10.1126/science.aan5951 PMC6035749

[61] Choueiri TK, Motzer RJ. Systemic therapy for metastatic renal-cell carcinoma. N Engl J Med. 2017;376:354–66. 28121507 10.1056/NEJMra1601333

[62] Elisi GM, Santucci M, D’Arca D, Lauriola A, Marverti G, Losi L, et al. Repurposing of drugs targeting YAP-TEAD functions. Cancers. 2018;10:329. 10.3390/cancers10090329 PMC6162436 30223434

