# Supplementary material for: OSCAR functions as a collagen I receptor to suppress hippo signaling and reprogram lipid metabolism in clear-cell renal cell carcinoma
Source: Cell Death Dis. 2026 Apr 8;17(1):499. doi: 10.1038/s41419-026-08713-1 (PMC13187165; doi:10.1038/s41419-026-08713-1)
Supplement: Supplementary file 1 — Supplemental file [file 41419_2026_8713_MOESM1_ESM.docx]

Supporting Information

**OSCAR Functions as a Collagen I Receptor to Suppress Hippo Signaling and Reprogram Lipid Metabolism in Clear-Cell Renal Cell Carcinoma**


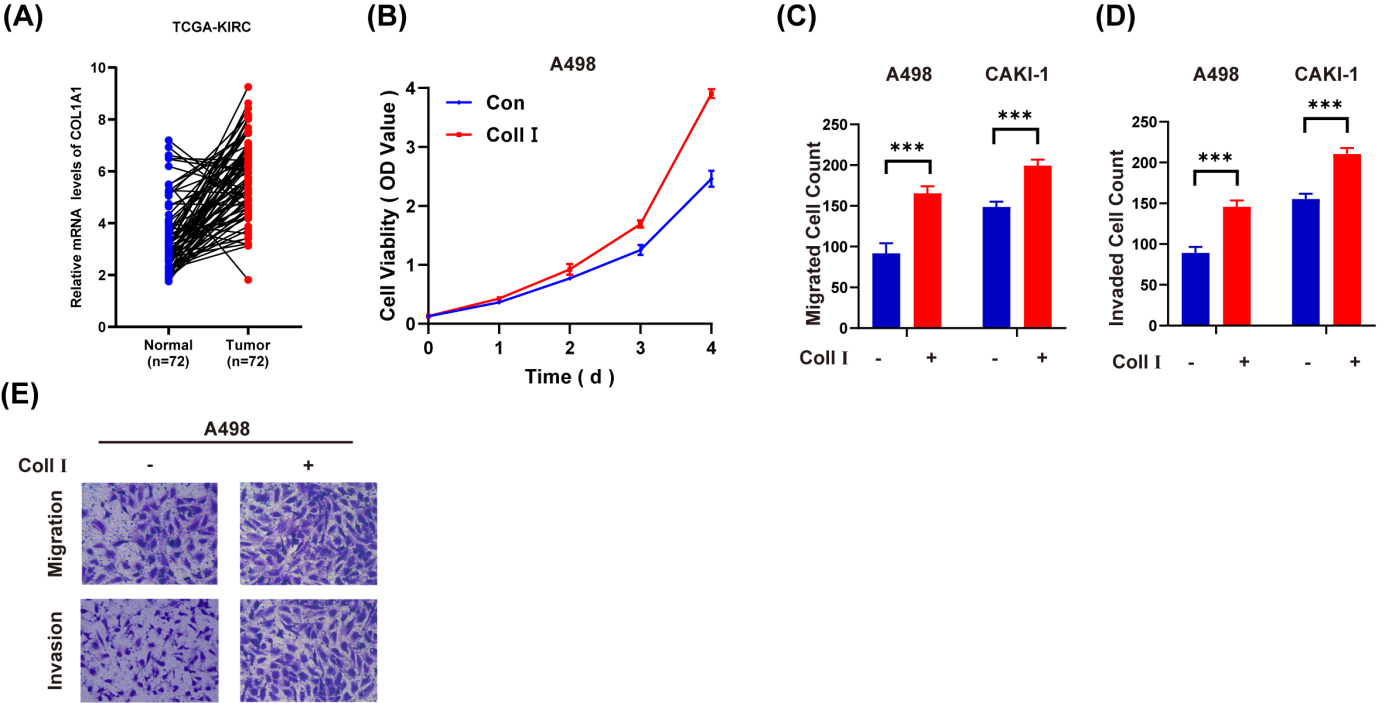
**FigureS1**

Collagen I is upregulated in ccRCC and promotes tumor progression.(A) Analysis of the TCGA (KIRC) database (t-test) showing the mRNA levels of COL1A1 in ccRCC tissues and paired adjacent normal tissues.(B) CCK-8 assay showing the proliferation curves of collagen I-treated cells and control cells (n = 3, t-test).(C–E) Transwell assays comparing the migration and invasion abilities of collagen I-treated cells and control cells (n = 3, t-test).Data represent at least three independent experiments (*P < 0.05, **P < 0.01, ***P < 0.001).


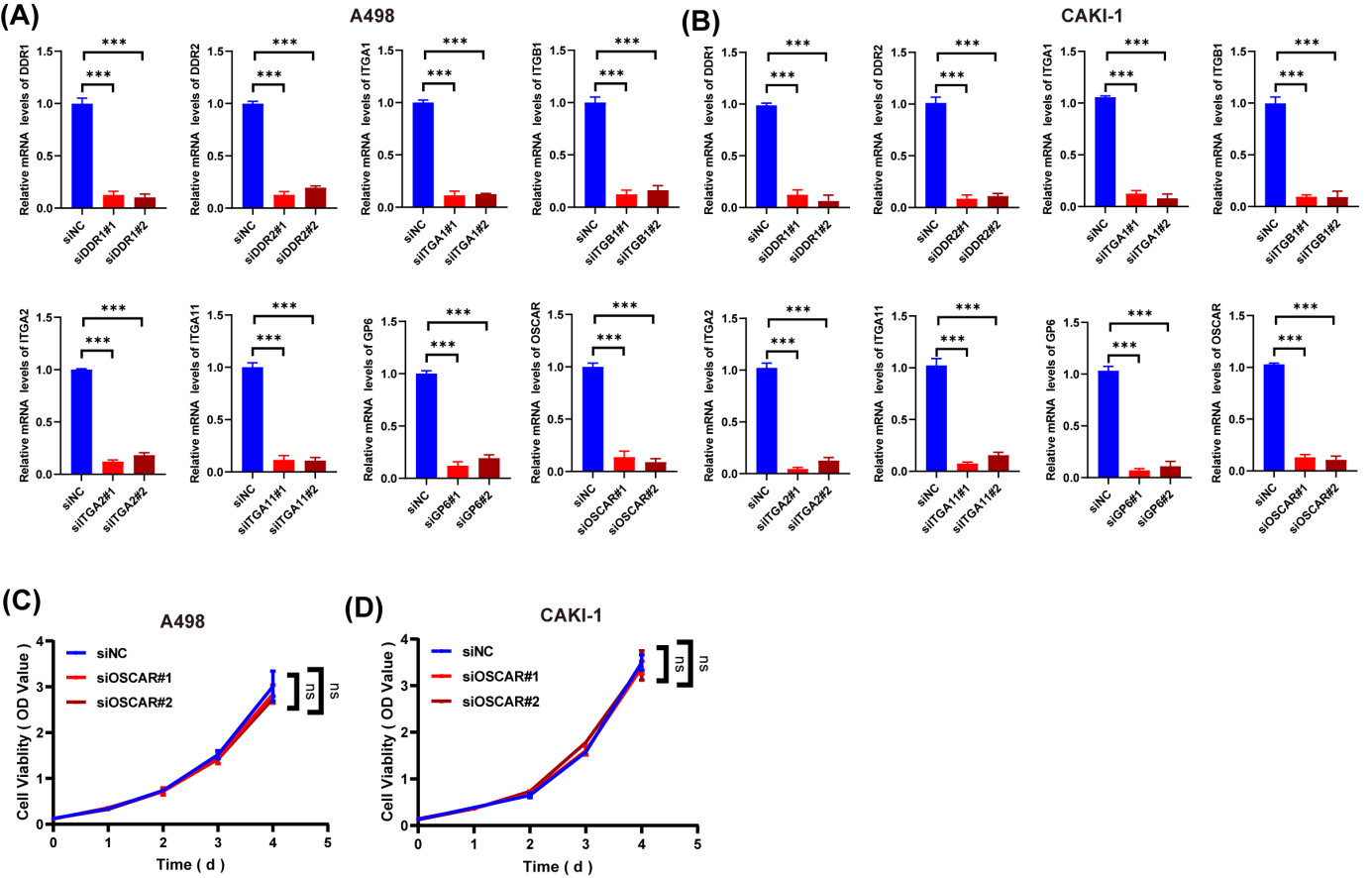


**FigureS2**

OSCAR was identified as a potential receptor for collagen I in ccRCC.(A–B) mRNA expression levels of the corresponding genes in cells with knockdown of DDR1, DDR2, ITGA1, ITGA2, ITGB1, ITGA11, or GP6 (n = 3, one-way ANOVA followed by Dunnett’s post-hoc test).(C–D) CCK-8 proliferation curves of OSCAR knockdown cells and control cells without collagen I treatment (n=3, two-way ANOVA with appropriate post-hoc tests).Data represent at least three independent experiments (*P < 0.05, **P < 0.01, ***P < 0.001).


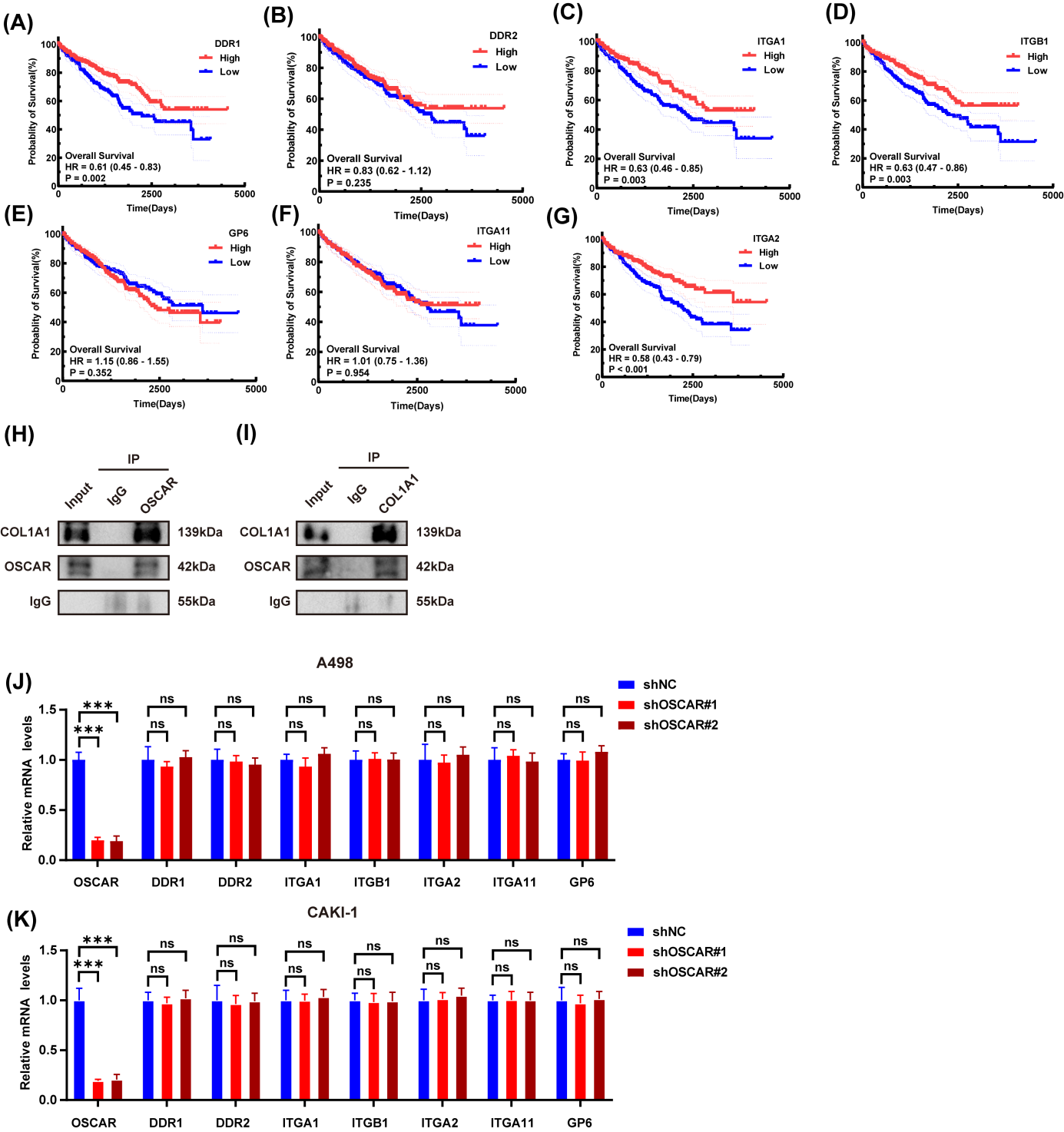


**FigureS3**

Among the known receptors of collagen I, only high OSCAR expression is significantly associated with poor prognosis in ccRCC patients.(A–G) Kaplan–Meier analysis showing the correlation between overall survival of ccRCC patients and the expression levels of DDR1, DDR2, ITGA1, ITGA2, ITGB1, ITGA11, and GP6. Patients were stratified into high- and low-expression subgroups based on the median expression level (50%) using the log-rank test.(H-I)HEK293T cells without plasmid transfection were treated with collagen I, and cell lysates were subjected to Co-IP and Western blotting as a negative control. (J-K) qPCR analysis of OSCAR and indicated collagen I receptor mRNA levels in A498 and CAKI-1 cells following OSCAR knockdown (n = 3, one-way ANOVA followed by Dunnett’s post-hoc test).

**
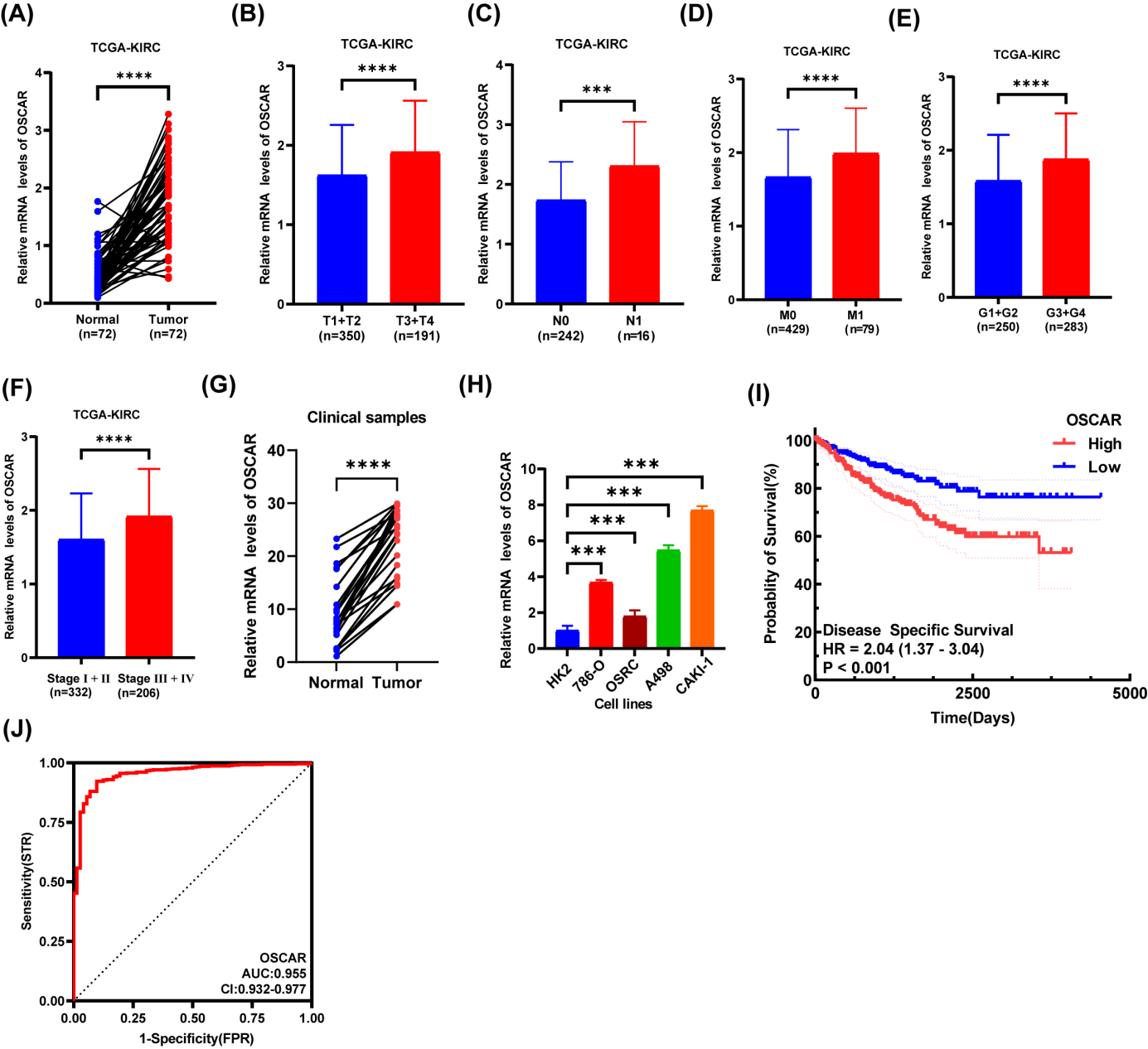
FigureS4**

OSCAR is highly expressed in ccRCC and indicates poor prognosis.(A) Data from the TCGA (KIRC) database (t-test) comparing **OSCAR** mRNA levels between ccRCC tissues and paired adjacent normal tissues.(B–F) Data from the TCGA (KIRC) database (t-test) showing **OSCAR** mRNA levels in ccRCC tissues of patients with different T stage, N stage, M stage, tumor grade, and clinical stage.(G) **OSCAR** mRNA levels in ccRCC tissues and paired adjacent normal tissues from 24 patients (t-test). (H )OSCAR mRNA expression in ccRCC versus normal renal epithelial cell lines.(I) Kaplan–Meier analysis showing the correlation between disease-specific survival (DSS) and **COL1A1** expression levels in ccRCC patients. Patients were stratified into high- and low-expression groups using the median expression level (50%) as the cutoff (log-rank test).(J) ROC analysis based on the TCGA dataset comparing **OSCAR** mRNA expression levels between ccRCC tumor tissues and normal kidney tissues (AUC = 0.955).(*P < 0.05, **P < 0.01, ***P < 0.001)


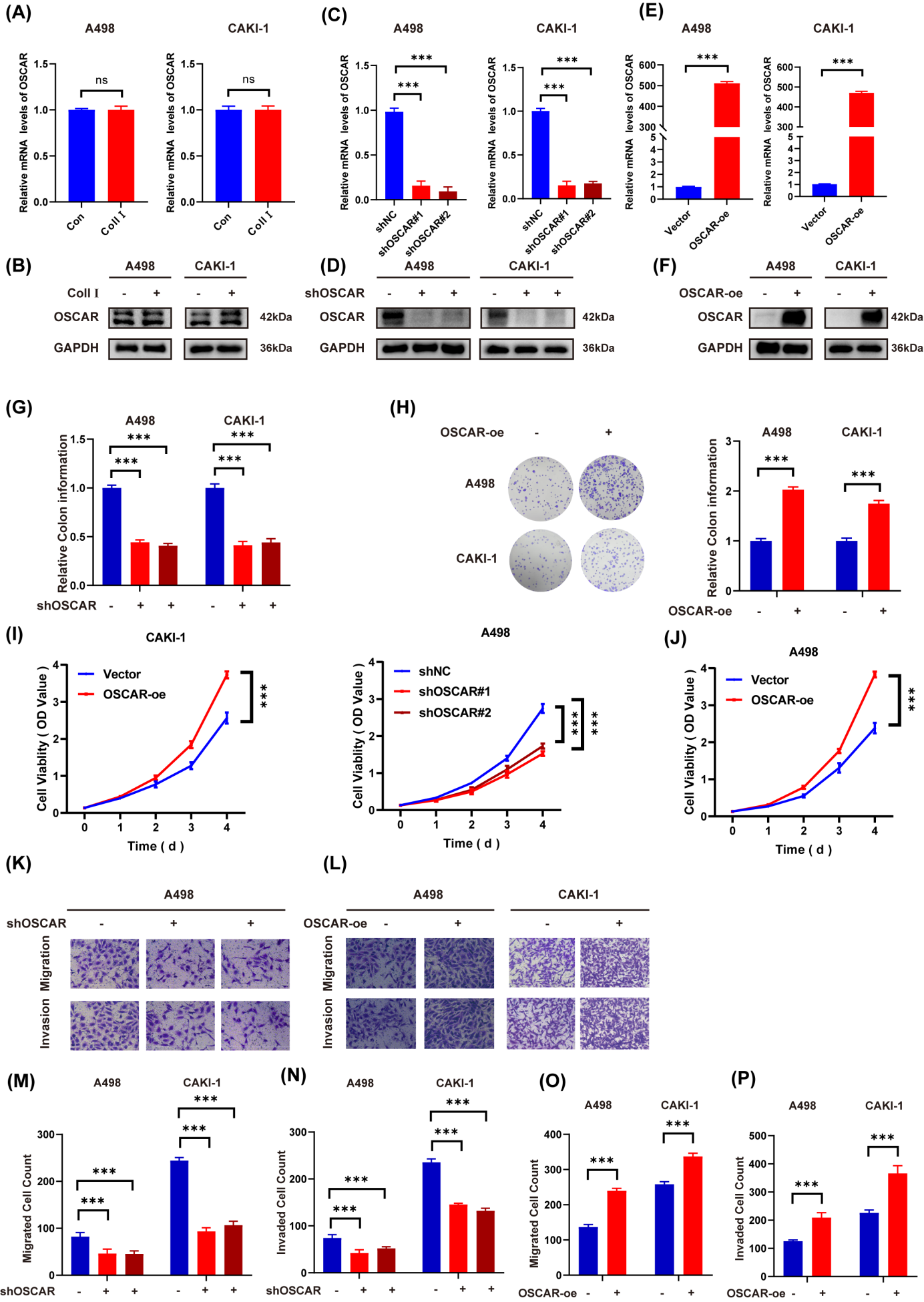
**FigureS5**

In ccRCC, OSCAR promotes tumor progression in vitro.(A) Comparison of OSCAR mRNA levels between collagen I-treated cells and control cells (n = 3, t-test).(B) Western blot analysis showing OSCAR protein levels in collagen I-treated cells and control cells.(C) Comparison of OSCAR mRNA levels between OSCAR-knockdown cells and control cells (n = 3, one-way ANOVA followed by Dunnett’s post-hoc test).(D) Western blot analysis showing OSCAR protein levels in OSCAR-knockdown cells and control cells.(E) Comparison of OSCAR mRNA levels between OSCAR-overexpressing cells and control cells (n = 3, t-test).(F) Western blot analysis showing OSCAR protein levels in OSCAR-overexpressing cells and control cells.(G) Colony formation assay of collagen I-treated OSCAR-knockdown ccRCC cells and control cells (n = 3, one-way ANOVA followed by Dunnett’s post-hoc test). (H) Colony formation assay of collagen I-treated OSCAR-overexpressing ccRCC cells and control cells (n = 3, t-test).(I–J) CCK-8 proliferation curves of OSCAR-knockdown or OSCAR-overexpressing cells and control cells after collagen I treatment (n=3, t-test and two-way ANOVA with appropriate post-hoc tests).(K–P) Transwell migration and invasion assays of OSCAR-overexpressing or OSCAR-knockdown cells and control cells after collagen I treatment (n = 3, t-test and one-way ANOVA followed by Dunnett’s post-hoc test).All results represent at least three independent experiments (*P < 0.05, **P < 0.01, ***P < 0.001).


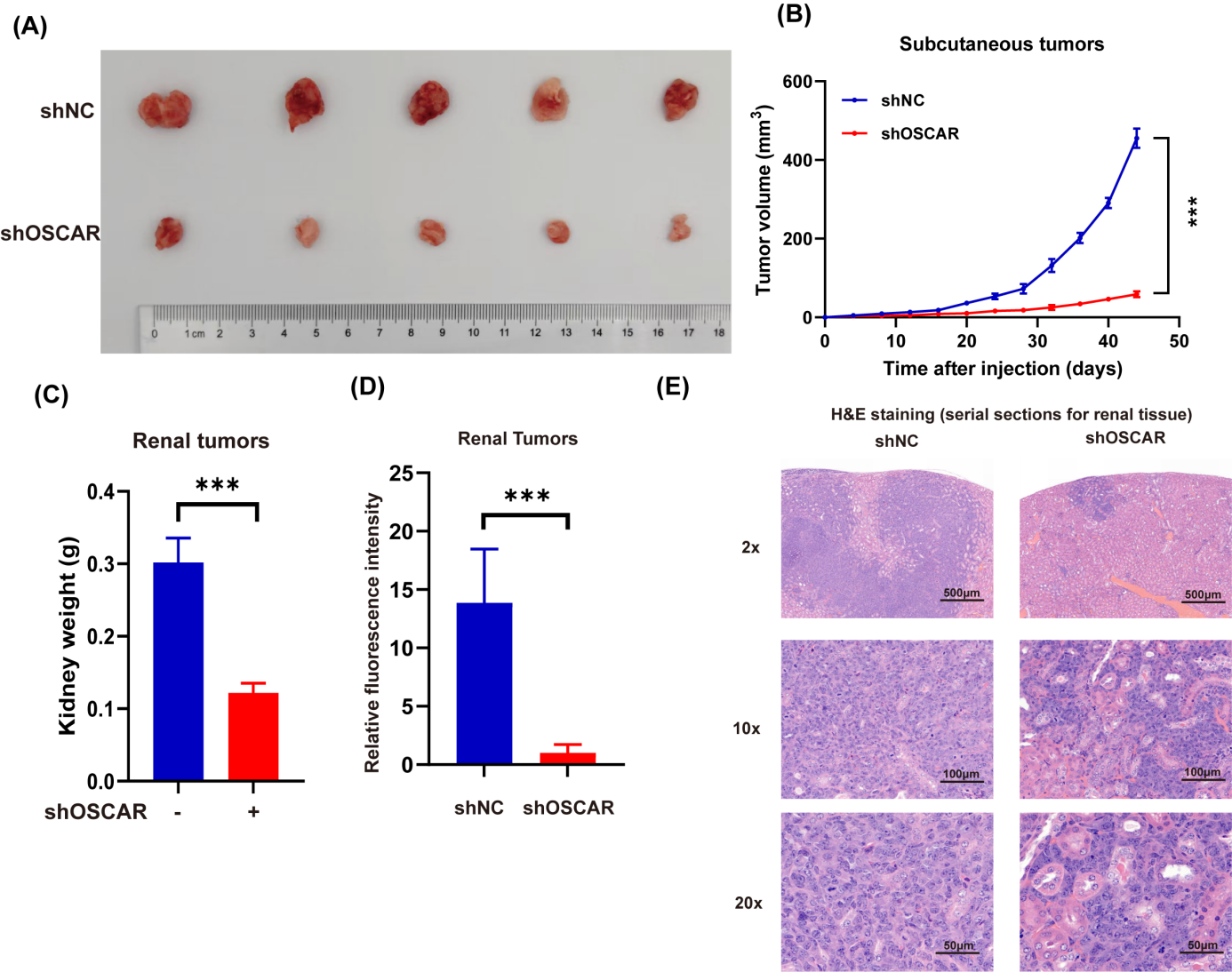
**FigureS6**

In ccRCC, OSCAR promotes tumor progression in vivo. (A) Stable OSCAR-knockdown CAKI-1 cells were subcutaneously injected into nude mice (n = 5), and representative images of the excised tumors were captured after euthanasia. (B) Tumor volumes were measured every 4 days until day 44 (n = 5, t-test). (C) Stable OSCAR-knockdown CAKI-1 cells were orthotopically injected into the kidneys of nude mice, and kidney weights were measured upon euthanasia (n = 5, t-test). (D) Bioluminescence imaging was used to assess the luminescence intensity of orthotopic kidney tumors (n = 5, t-test). (E) H&E staining of kidney tissues from orthotopic tumor models in OSCAR-knockdown and control groups. Data are representative of at least three independent experiments (*P < 0.05, **P < 0.01, ***P < 0.001).


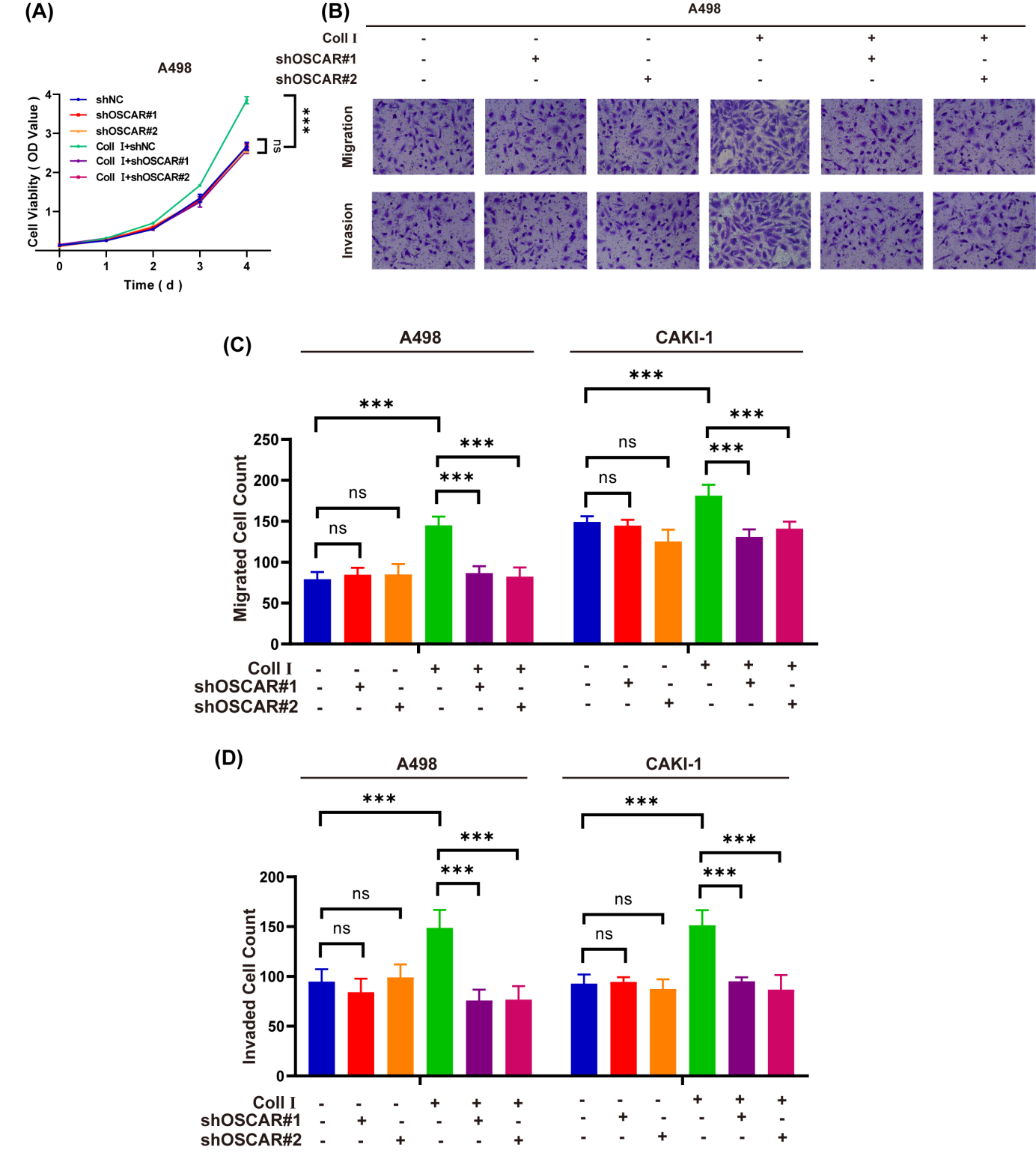
**FigureS7**

In ccRCC, collagen I primarily promotes tumor progression through the receptor OSCAR. (A) CCK-8 assay showing cell proliferation curves for the indicated A498 cell treatments (n=3, two-way ANOVA with appropriate post-hoc tests). (B–D) Transwell assays of the indicated ccRCC cell treatments (n = 3, one-way ANOVA followed by Dunnett’s post-hoc test). Data are representative of at least three independent experiments (*P < 0.05, **P < 0.01, ***P < 0.001).


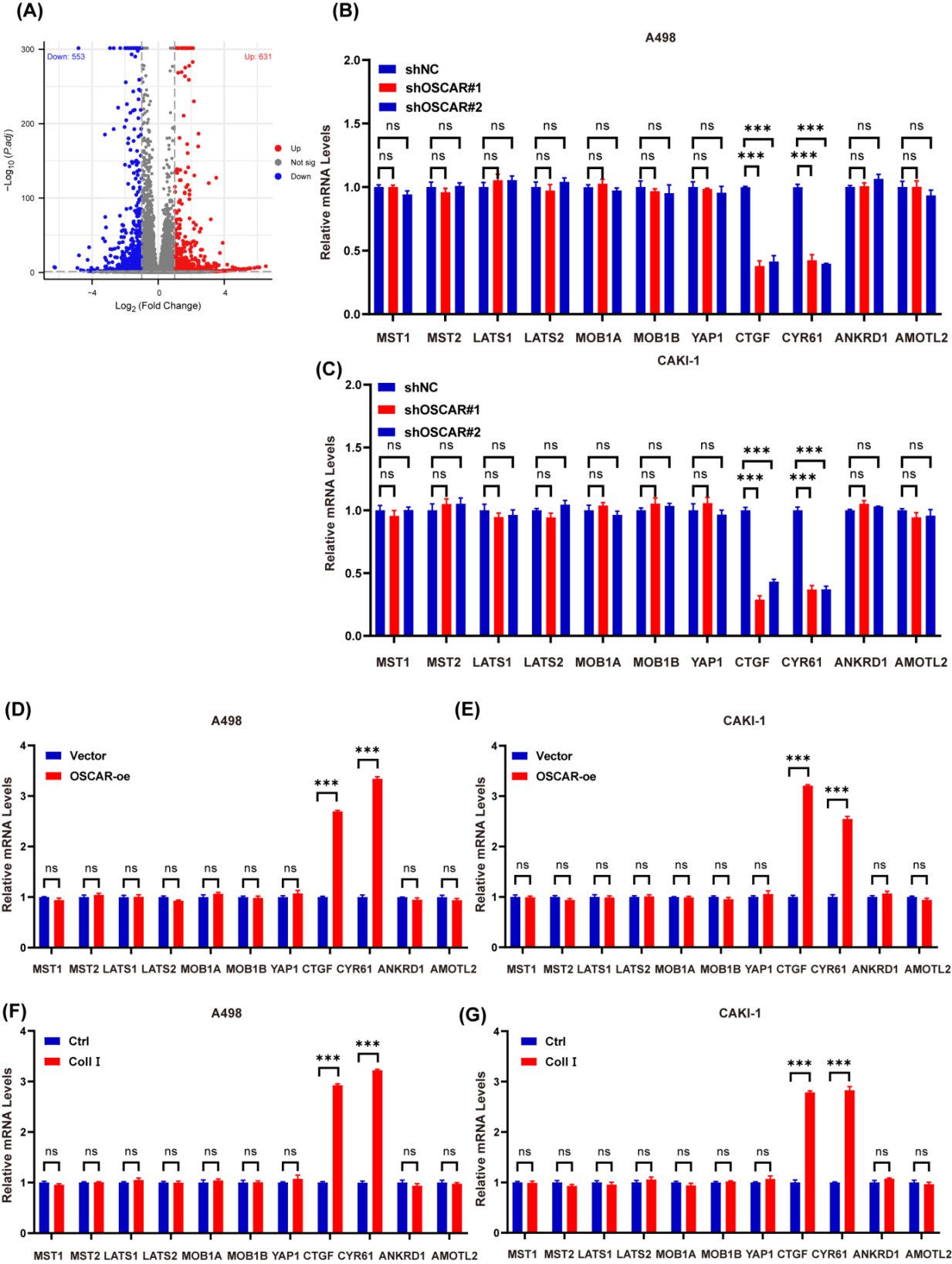


**FigureS8**

Collagen I–OSCAR inhibits the Hippo pathway. (A) Volcano plot of RNA-seq results comparing OSCAR knockdown cells with control cells after collagen I treatment. (B–C) mRNA levels of key Hippo pathway components in OSCAR knockdown and control cells after collagen I treatment(n = 3, one-way ANOVA followed by Dunnett’s post-hoc test). (D–E) mRNA levels of key Hippo pathway components in OSCAR-overexpressing and control cells after collagen I treatment(n = 3, one-way ANOVA followed by Dunnett’s post-hoc test). (F–G) mRNA levels of key Hippo pathway components in cells treated with collagen I compared with control cells(n = 3, one-way ANOVA followed by Dunnett’s post-hoc test). Data are representative of at least three independent experiments (*P < 0.05, **P < 0.01, ***P < 0.001).


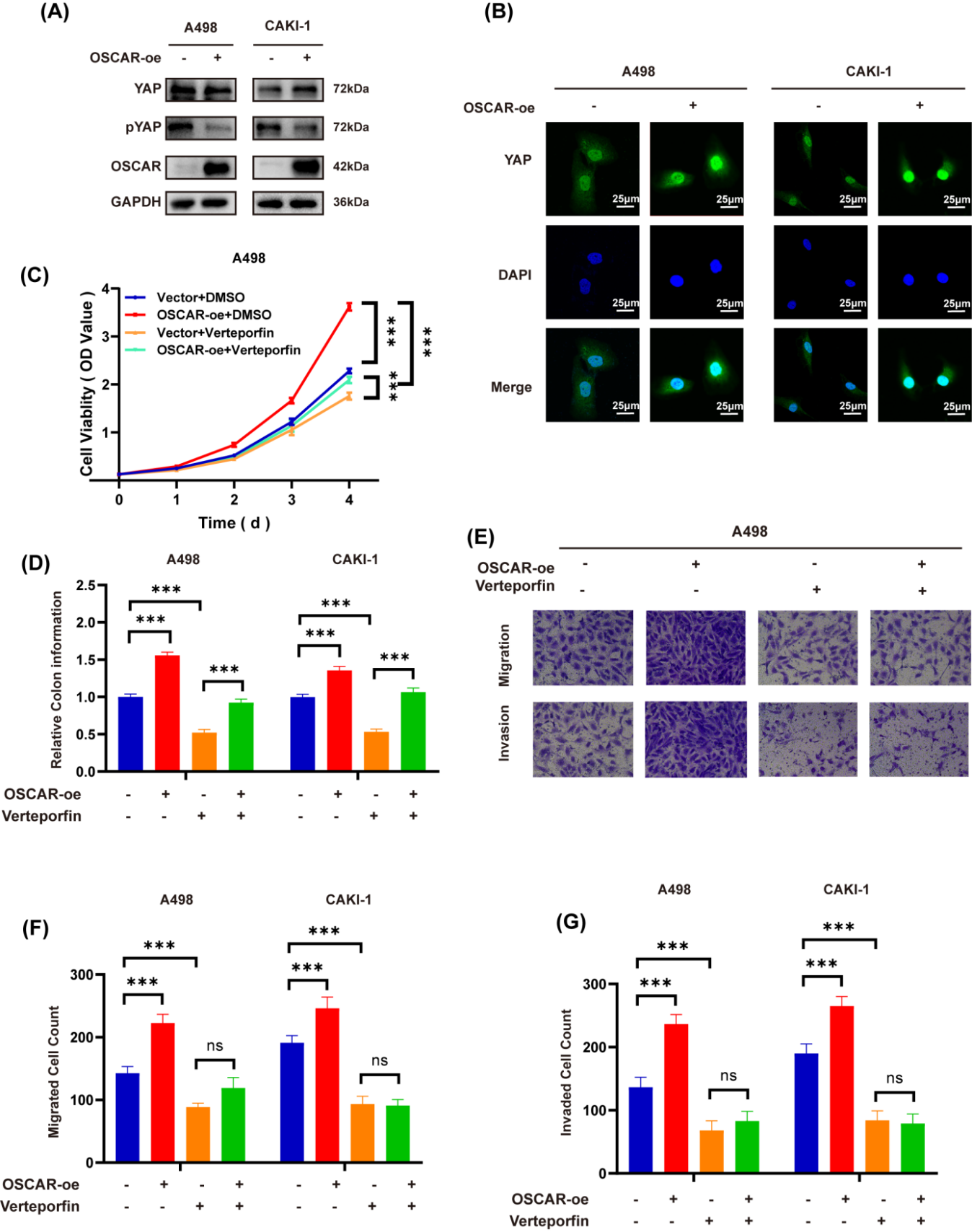


**FigureS9**

Collagen I–OSCAR promotes ccRCC progression primarily by inhibiting the Hippo pathway. (A) Western blotting showing the levels of phosphorylated YAP and total YAP in OSCAR-overexpressing and control cells after collagen I treatment. (B) Immunofluorescence analysis showing the subcellular localization of YAP in OSCAR-overexpressing cells following collagen I treatment. (C) CCK-8 assay of cell proliferation curves in the indicated ccRCC cells (n=3, two-way ANOVA with appropriate post-hoc tests). (D) Colony formation assay in the indicated ccRCC cells(n = 3, one-way ANOVA followed by Dunnett’s post-hoc test). (L) Transwell assay in the indicated ccRCC cells (n = 3, one-way ANOVA followed by Dunnett’s post-hoc test). Data represent at least three independent experiments (*P < 0.05, **P < 0.01, ***P < 0.001).


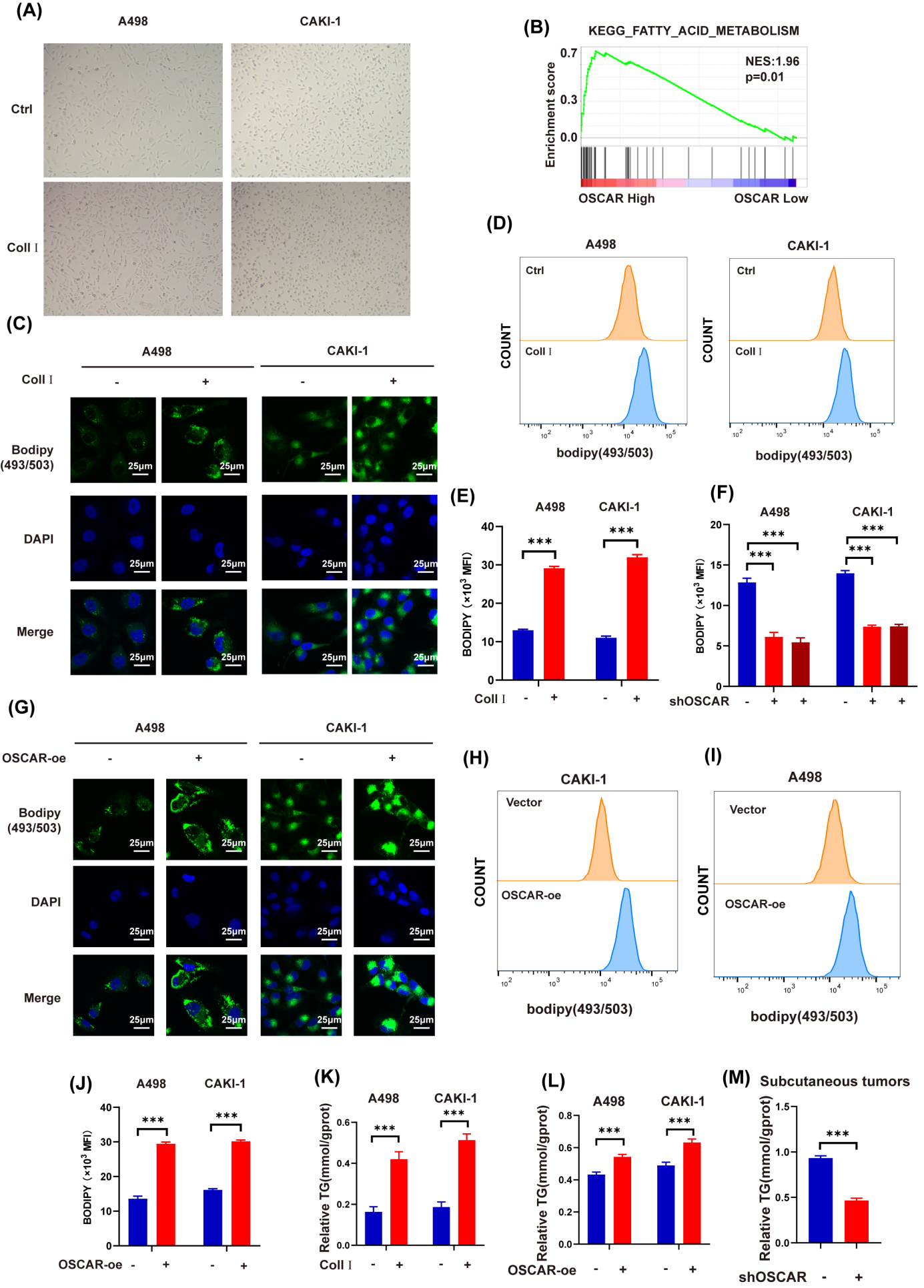
**FigureS10**

Collagen I–OSCAR induces lipid droplet accumulation in ccRCC cells. (A) Microscopic images of cells treated with collagen I and control cells. (B) GSEA analysis showing the correlation between lipid metabolism and OSCAR mRNA levels in ccRCC. (C–E) Confocal microscopy and flow cytometry analysis of lipid droplet levels in Bodipy (498/503)-stained collagen I-treated and control ccRCC cells (n = 3, statistical analysis: t-test). (F) Flow cytometry analysis of lipid droplet levels in Bodipy (498/503)-stained collagen I-treated OSCAR knockdown and control ccRCC cells (n = 3, one-way ANOVA followed by Dunnett’s post-hoc test). (G–J) Confocal microscopy and flow cytometry analysis of lipid droplet levels in Bodipy (498/503)-stained collagen I-treated OSCAR-overexpressing and control ccRCC cells (n = 3, statistical analysis: t-test). (K) Relative TG content (mmol/gprot) in collagen I-treated and control ccRCC cells determined using a TG assay kit (n = 3, statistical analysis: t-test). (L) Relative TG content (mmol/gprot) in collagen I-treated OSCAR-overexpressing and control ccRCC cells determined using a TG assay kit (n = 3, statistical analysis: t-test). (M) Relative TG content (mmol/gprot) in subcutaneous tumors from OSCAR knockdown and control ccRCC cells determined using a TG assay kit (n = 3, statistical analysis: t-test). Data represent at least three independent experiments (*P < 0.05, **P < 0.01, ***P < 0.001).


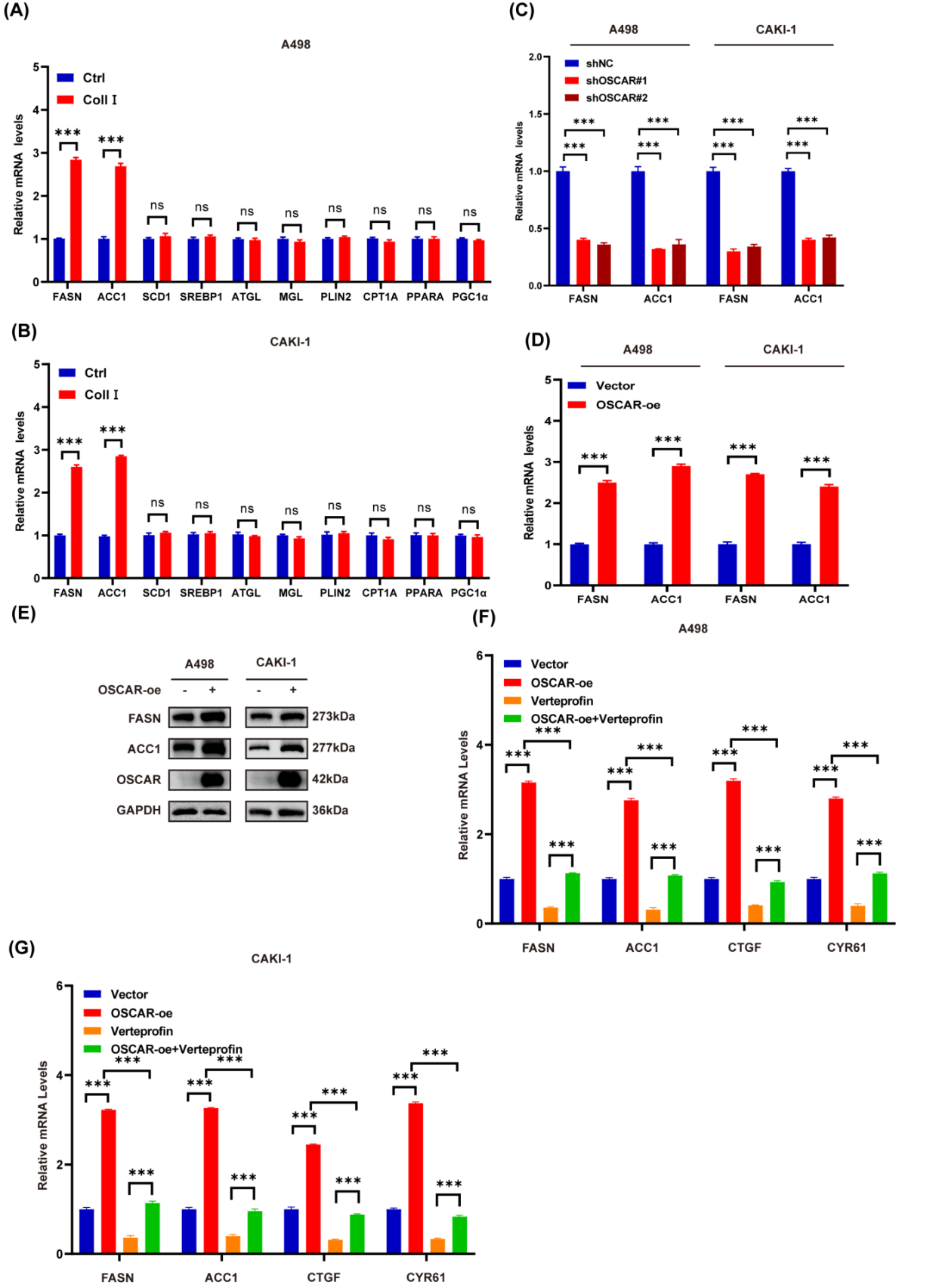


**FigureS11**

Collagen I–OSCAR promotes lipid synthesis by inhibiting the Hippo pathway. (A–B) Comparison of mRNA levels of key lipid metabolism genes between collagen I-treated and control cells(n = 3, one-way ANOVA followed by Dunnett’s post-hoc test). (C) mRNA levels of FASN and ACC1 in collagen I-treated OSCAR knockdown and control cells(n = 3, one-way ANOVA followed by Dunnett’s post-hoc test). (D) mRNA levels of FASN and ACC1 in collagen I-treated OSCAR-overexpressing and control cells (n = 3, statistical analysis: t-test). (E–F) mRNA levels of FASN, ACC1, CTGF, and CYR61 in the indicated treated and control cells (n = 3, one-way ANOVA followed by Dunnett’s post-hoc test). Data represent at least three independent experiments (*P < 0.05, **P < 0.01, ***P < 0.001).


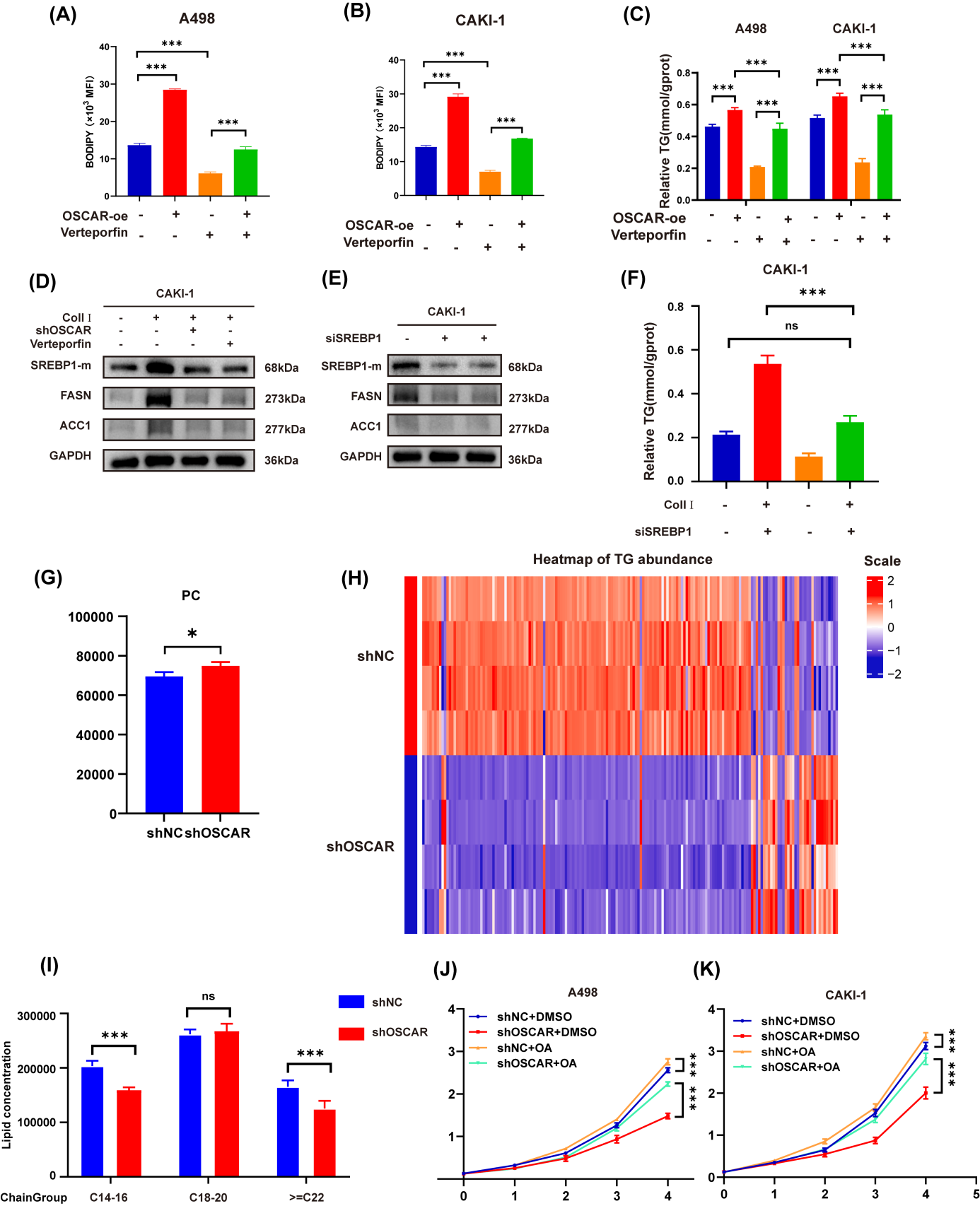
**FigureS12**

Collagen I–OSCAR induces lipid reprogramming in ccRCC by inhibiting the Hippo pathway. (A–B) Flow cytometry analysis of lipid droplet levels in Bodipy (498/503)-stained cells under the indicated treatments (n = 3, one-way ANOVA followed by Dunnett’s post-hoc test). (C) Relative triglyceride (TG) levels (mmol/gprot) in cells under the indicated treatments, assessed using a TG assay kit (n = 3, one-way ANOVA followed by Dunnett’s post-hoc test). (D) Immunoblot analysis of mature SREBP1 (SREBP1-m), FASN, and ACC1 in CAKI-1 cells treated with collagen I in the presence or absence of OSCAR knockdown or YAP inhibition by verteporfin.(E) Immunoblot analysis of SREBP1-m, FASN, and ACC1 in CAKI-1 cells transfected with control or SREBP1-targeting siRNA.

(F) Quantification of intracellular triglyceride (TG) levels in CAKI-1 cells treated with collagen I with or without SREBP1 knockdown(n = 3, one-way ANOVA followed by Dunnett’s post-hoc test). (G) Lipidomic analysis of phosphatidylcholine (PC) content in collagen I-treated OSCAR knockdown and control cells (n = 4, statistical analysis: t-test). (H) Lipidomic analysis of the effects of collagen I treatment on triglyceride (TG) levels in OSCAR knockdown and control cells. (I) Quantification of fatty acid species grouped by chain length (C14–16, C18–20, and ≥C22) in CAKI-1 cells following OSCAR knockdown. (J-K) Proliferation curves of A498 and CAKI-1 cells cultured under collagen I stimulation and transfected with control or OSCAR-targeting shRNA, with or without oleic acid (OA) supplementation, assessed by CCK-8 assays (n=3, two-way ANOVA with appropriate post-hoc tests). Data represent at least three independent experiments (*P < 0.05, **P < 0.01, ***P < 0.001).


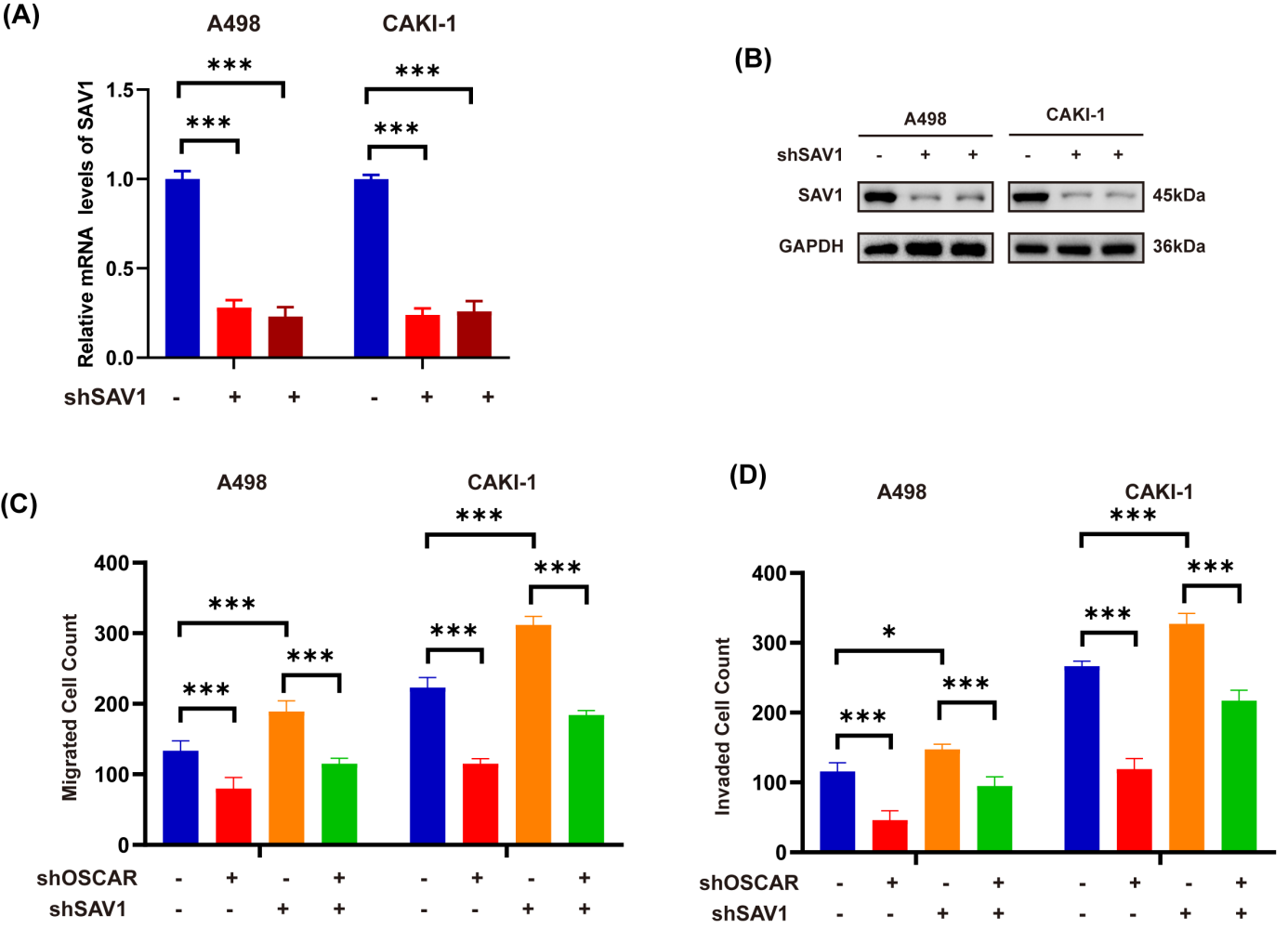
**FigureS13**

SAV1 is a functional target of the collagen I–OSCAR axis in promoting ccRCC progression. (A) SAV1 mRNA levels in SAV1 knockdown and control cells(n = 3, one-way ANOVA followed by Dunnett’s post-hoc test). (B) Western blotting analysis of OSCAR protein levels in SAV1 knockdown and control cells. (C–D) Transwell assays of cells under the indicated treatments(n = 3, one-way ANOVA followed by Dunnett’s post-hoc test). Data represent at least three independent experiments (*P < 0.05, **P < 0.01, ***P < 0.001).


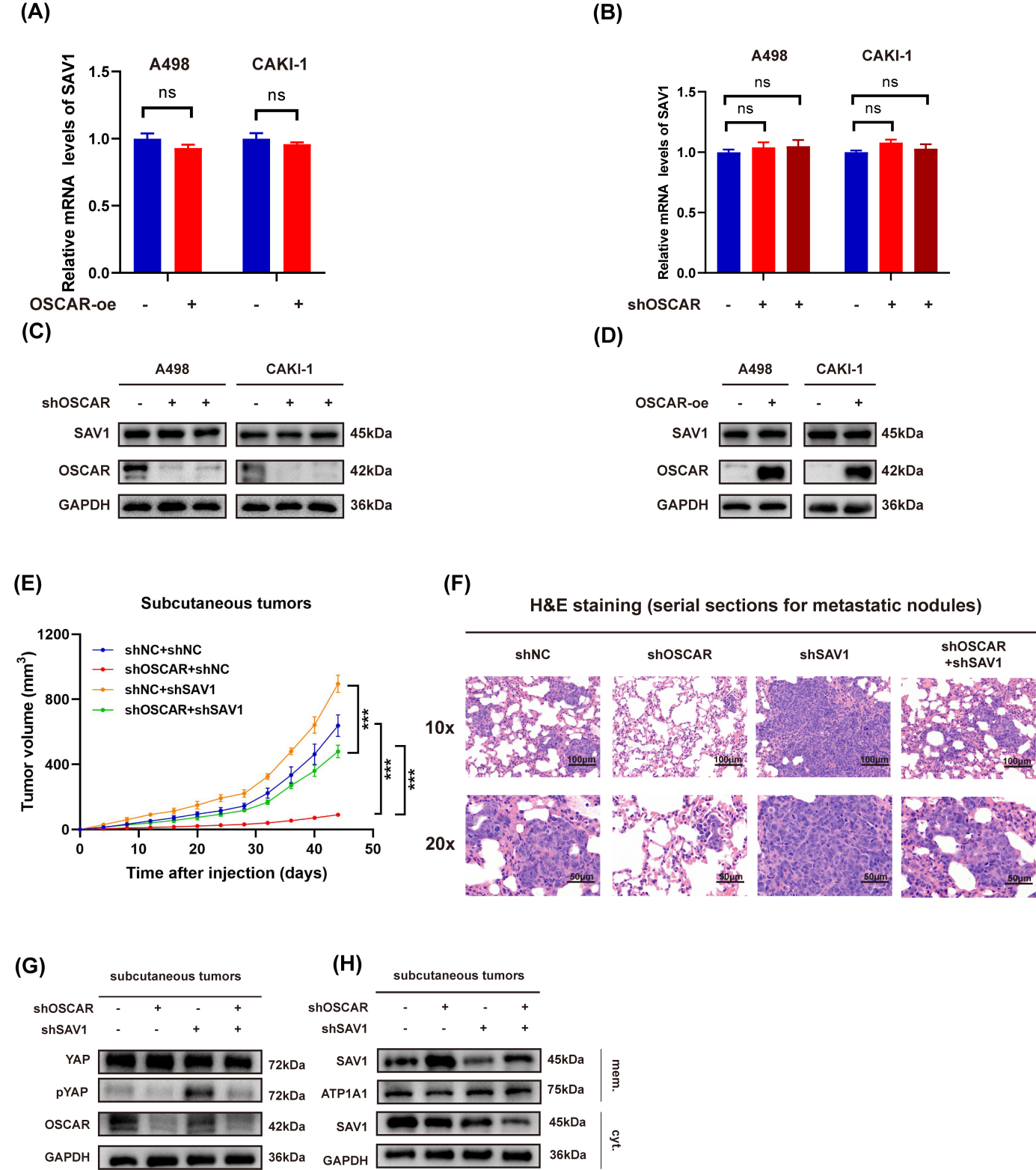
**FigureS14**

Collagen I–OSCAR promotes ccRCC progression by restricting the membrane localization of SAV1. (A–B) SAV1 mRNA levels in OSCAR knockdown or overexpression cells compared with control cells (n = 3, statistical analysis: t-test and one-way ANOVA followed by Dunnett’s post-hoc test). (C–D) Western blotting analysis of SAV1 protein levels in OSCAR knockdown or overexpression cells compared with control cells. (E) Stable OSCAR knockdown CAKI-1 cells were subcutaneously injected into nude mice (n = 5), and tumor volumes were measured every 4 days until day 44 (n=5, two-way ANOVA with appropriate post-hoc tests). (F) Representative H&E staining images of lung tissues from the indicated metastatic models.(G) Immunoblot analysis of YAP, phosphorylated YAP (p-YAP), and OSCAR in subcutaneous tumor tissues derived from control, shOSCAR, shSAV1, or combined shOSCAR + shSAV1 xenografts.(H) Subcellular fractionation followed by immunoblot analysis of SAV1 in membrane and cytoplasmic fractions of subcutaneous tumor tissues. Data represent at least three independent experiments (*P < 0.05, **P < 0.01, ***P < 0.001).

**
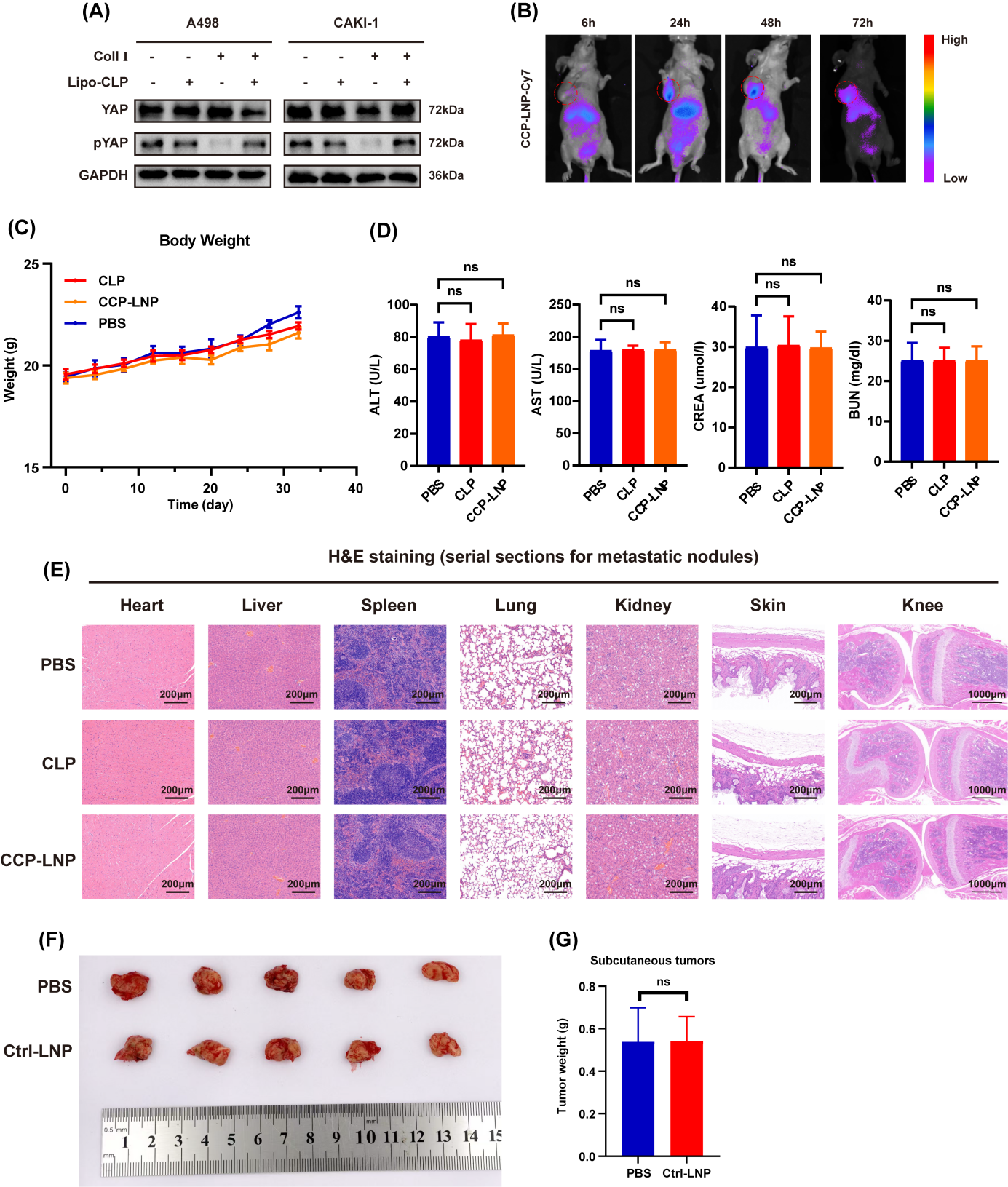
FigureS15**

CCP-LNP targeting the collagen I–OSCAR axis suppresses ccRCC progression. (A) Western blotting analysis of total YAP and phosphorylated YAP levels in the indicated treated cells. (B) Time-dependent in vivo biodistribution and tumor retention of CCP-LNP.(C) CAKI-1 cells were subcutaneously implanted into nude mice (n = 5), and body weight was monitored every 4 days during treatment until day 32. (D) Serum liver and kidney function parameters were assessed in each group (n = 3, one-way ANOVA followed by Dunnett’s post-hoc test). (E) Representative H&E staining images of major organs from each treatment group.(F) Representative images of excised subcutaneous tumors from mice treated with PBS or Ctrl-LNP.(G) Quantification of tumor weight from PBS and Ctrl-LNP-treated mice (n = 5, t-test).

# **Supplementary Tables**

**Supplementary Table S1** Univariate and multivariate Cox analyses of overall survival of 534 patients with ccRCC.

| Characteristics | Total(N) | Univariate analysis | Univariate analysis | Multivariate analysis | Multivariate analysis |
| --- | --- | --- | --- | --- | --- |
|  |  | HR(95% CI) | P value | HR(95% CI) | P value |
| Pathologic T stage | 532 |  |  |  |  |
| T1 | 272 | Reference |  | Reference |  |
| T2&T3&T4 | 260 | 2.838 (2.044 - 3.940) | < 0.001 | 0.569 (0.166 - 1.952) | 0.370 |
| Pathologic N stage | 256 |  |  |  |  |
| N0 | 240 | Reference |  | Reference |  |
| N1 | 16 | 3.395 (1.803 - 6.395) | < 0.001 | 1.730 (0.856 - 3.494) | 0.127 |
| Pathologic M stage | 500 |  |  |  |  |
| M0 | 421 | Reference |  | Reference |  |
| M1 | 79 | 4.343 (3.184 - 5.924) | < 0.001 | 3.254 (2.009 - 5.270) | < 0.001 |
| Pathologic stage | 529 |  |  |  |  |
| Stage I | 266 | Reference |  | Reference |  |
| Stage II&Stage III&Stage IV | 263 | 3.202 (2.280 - 4.496) | < 0.001 | 2.765 (0.750 - 10.195) | 0.127 |
| Gender | 532 |  |  |  |  |
| Female | 187 | Reference |  |  |  |
| Male | 345 | 0.944 (0.694 - 1.284) | 0.714 |  |  |
| Age | 532 |  |  |  |  |
| <= 60 | 264 | Reference |  | Reference |  |
| > 60 | 268 | 1.779 (1.310 - 2.416) | < 0.001 | 1.861 (1.215 - 2.848) | 0.004 |
| OSCAR | 532 |  |  |  |  |
| Low | 266 | Reference |  | Reference |  |
| High | 266 | 1.761 (1.296 - 2.394) | < 0.001 | 1.616 (1.032 - 2.531) | 0.036 |

## **Supplementary Table S2** siRNA and shRNA target sequences

| Gene | Sequences (5'- -3') |
| --- | --- |
| SiDDR1#1 | CCUGGUUACUCUUCAGCGAAA**TT** |
| SiDDR1#2 | GUGUGGCUCGCUUUCUGCAGU**TT** |
| SiDDR2#1 | CCGUCCCUCAUUCCAAGAAAU**TT** |
| SiDDR2#2 | GCCAACAAGAAUGCCAGGAAU**TT** |
| SiGP6#1 | CAACUGAGACUCUAGGAGUA**TT** |
| SiGP6#2 | CCUACAAGAAUCCCGAGAGAU**TT** |
| SiITGA1#1 | GCACGACAUCAGUGGCAAUAA**TT** |
| SiITGA1#2 | GCUCUUACUUUGGGAGUGAAATT |
| SiITGA2#1 | CCUCAGAAGCUAUCCCUAAAU**TT** |
| SiITGA2#2 | CCGGCCAGAUAGUGCUAUAUATT |
| SiITGA11#1 | GCUCUUACUUUGGGAGUGAAA**TT** |
| SiITGA11#2 | GCACGACAUCAGUGGCAAUAA**TT** |
| SiITGB1#1 | CCAAAUCAUGUGGAGAAUGUA**TT** |
| SiITGB1#2 | GCCUUGCAUUACUGCUGAUAU**TT** |
| SiOSCAR#1 | GCCUCUGUGUCACACAGACAU**TT** |
| SiOSCAR#2 | GCGGAACAUGAGCUUCGUGCU**TT** |
| ShOSCAR#1 | GCCTCTGTGTCACACAGACAT |
| ShOSCAR#2 | GCGGAACATGAGCTTCGTGCT |
| ShSAV1#1 | CGGCTACATCTCTAGGGAATT |
| ShSAV1#2 | CGAGTAGAGTCATCAGAATTT |

## **Supplementary Table S3** Sequences of primers of qPCR

| Gene | | Sequences (5'- -3') |
| --- | --- | --- |
| COL1A1 | Forward | TGACGAGACCAAGAACTGCC |
|  | Reverse | GCACCATCATTTCCACGAGC |
| GAPDH | Forward | CCAGAACATCATCCCTGCCT |
|  | Reverse | CCTGCTTCACCACCTTCTTG |
| DDR1 | Forward | CGGGAAGAGCGATGAGAGAG |
|  | Reverse | AGACAGGAGTCCATCCCTCC |
| DDR2 | Forward | TTGCATCAGCCTGTGGATGT |
|  | Reverse | GAGTCCAGCCAAAGGTCTCC |
| GP6 | Forward | CCCTCTTCTGTCTTGGGCTG |
|  | Reverse | GTAGGGTTACGTCCCCTCCT |
| ITGA1 | Forward | GGGAAGCTGCCAGTGAGATT |
|  | Reverse | GGCCAACTAACGGAGAACCA |
| ITGA2 | Forward | GGCTGGCCCAGAGTTTACAT |
|  | Reverse | ATCGCCCCCTCTCCTAACTT |
| ITGA11 | Forward | AGGGTTCACGGACACCTTCAA |
|  | Reverse | GCATGTTGTCTTTCCGCTCG |
| ITGB1 | Forward | CGCCGCGCGGAAAAGATG |
|  | Reverse | AAACACCAGCAGCCGTGTAA |
| OSCAR | Forward | GACATCACTCCGTCTGTCCC |
|  | Reverse | GGAGGGCGTGTGATAGTAGC |
| MST1 | Forward | CTGTGGGGCTGGTTCTGTAT |
|  | Reverse | GTTGACCTGCTACCCCAAAA |
| MST2 | Forward | CATGAGGAACAGCAACGAGA |
|  | Reverse | TATCACCATGGTCCCCAAGT |
| LATS1 | Forward | GACCTGGAATGCAGAATGGT |
|  | Reverse | ATGACGAAGGAGCAGCAGAT |
| LATS2 | Forward | TTCATCCACCGAGACATCAA |
|  | Reverse | CTCCATGCTGTCCTGTCTGA |
| MOB1A | Forward | TCTGCACTGAAGCAAGCTGT |
|  | Reverse | GGGCATAAACCCTGAACAGA |
| MOB1B | Forward | GTCCCGTTCCCAAAGAATTT |
|  | Reverse | CAGTTCTTGGAGTGGTGCAA |
| YAP | Forward | GCAGTTGGGAGCTGTTTCTC |
|  | Reverse | GCCATGTTGTTGTCTGATCG |
| CTGF | Forward | CACCCGGGTTACCAATGACA |
|  | Reverse | TCCGGGACAGTTGTAATGGC |
| CYR61 | Forward | CAGGACTGTGAAGATGCGGT |
|  | Reverse | GCCTGTAGAAGGGAAACGCT |
| ANKRD1 | Forward | AGAACTGTGCTGGGAAGACG |
|  | Reverse | GCCATGCCTTCAAAATGCCA |
| AMOTL2 | Forward | GGCTGACTACAGCAGACAGAG |
|  | Reverse | GCAAGTGAGAAGGTGAGGCT |
| FASN | Forward | CCCTCATCTCCCCACTCATC |
|  | Reverse | CAGCGTCTTCCACACTATGC |
| ACC1 | Forward | CAACTTTGTGCCCACGGTTA |
|  | Reverse | TTTGTCAGGAAGAGGCGGAT |
| SCD1 | Forward | CTTGCGATATGCTGTGGTGC |
|  | Reverse | CCGGGGGCTAATGTTCTTGT |
| SREBP1 | Forward | GTTTCCGAGGAACTTTTCGCCG |
|  | Reverse | GACTTCACCTTCGATGTCGGT |
| ATGL | Forward | TTCAACTCCAAGGACGAGCT |
|  | Reverse | GAACTGGATGCTGGTGTTGG |
| MGL | Forward | AAAGACTACCCTGGGCTTCC |
|  | Reverse | AAGGTTGAGCACTTTCGCAG |
| PLIN2 | Forward | AGTCTTGGGGAGTCGGATGA |
|  | Reverse | TGTCCATCTCTGCACCTTGG |
| CPT1A | Forward | CAGCATATGTATCGCCTCGC |
|  | Reverse | CTGGACACGTACTCTGGGTT |
| PPARA | Forward | TCCTCTCAGGAAAGGCCAGT |
|  | Reverse | ACAGAAGACAGCATGGCGAA |
| PGC1A | Forward | TGAAGGGTACTTTTCTGCCCC |
|  | Reverse | TTTGAGAAGCTCCGAGCAGG |
| SAV1 | Forward | ACGAGCCCCTGTGAAATATG |
|  | Reverse | CACTGCTGTCTCTGCTTTCG |

## **Supplementary Table S4** Antibodies used in this study

| Antibody | Vendors | Cat# | Working concentration (application) |
| --- | --- | --- | --- |
| COL1A1 | HUABIO | ET1609-68 | 1:1000(WB) |
| GAPDH | Proteintech | 60004-1-Ig | 1:10000(WB) |
| OSCAR | Proteintech | 21996-1-AP | 1:500(WB)1:200(IF) |
| YAP | Proteintech | 66900-1-Ig | 1:1000(WB)1:200(IF) |
| pYAP | CST | #13619 | 1:1000(WB) |
| Lamin B1 | Proteintech | 12987-1-AP | 1:10000(WB) |
| MST1 | HUABIO | HA500043 | 1:1000(WB) |
| pMST1 | CST | #49332 | 1:1000(WB) |
| LATS1 | Santa Cruz | sc-398560 | 1:250(WB) |
| pLATS1 | CST | #8654 | 1:1000(WB) |
| CTGF | ABclonal | A11067 | 1:1000(WB) |
| CYR61 | HUABIO | HA723070 | 1:1000(WB) |
| ATP1A1 | Proteintech | 14418-1-AP | 1:10000(WB) |
| FASN | ABclonal | A19050 | 1:1000(WB) |
| ACC1 | ABclonal | A15606 | 1:1000(WB) |
| FLAG | Sigma | F1804 | 1:10000(WB)1:100(IP) |
| HA | ABclonal | AE105 | 1:10000(WB)1:100(IP) |
| SAV1 | HUABIO | HA722130 | 1:1000(WB)1:200(IF) |
| Anti-mouse | Jackson ImmunoResearch | 115-035-003 | 1:5000(WB) |
| Anti-rabbit | Jackson ImmunoResearch | 111-035-003 | 1:5000(WB) |
| Anti-mouse-FITC | Jackson ImmunoResearch | 115-095-003 | 1:200(IF) |
| Anti-rabbit-FITC | Jackson ImmunoResearch | 111-095-003 | 1:200(IF) |
| Anti-rabbit-TRITC | Jackson ImmunoResearch | 111-025-003 | 1:200(IF) |
| 594 Antibody Labeling Kit for Rabbit IgG | Proteintech | KFA509 | 1 rxn(IF) |
