# Supplementary figures and images for: OSCAR functions as a collagen I receptor to suppress hippo signaling and reprogram lipid metabolism in clear-cell renal cell carcinoma
Source: Cell Death Dis. 2026 Apr 8;17(1):499. doi: 10.1038/s41419-026-08713-1 (PMC13187165; doi:10.1038/s41419-026-08713-1)

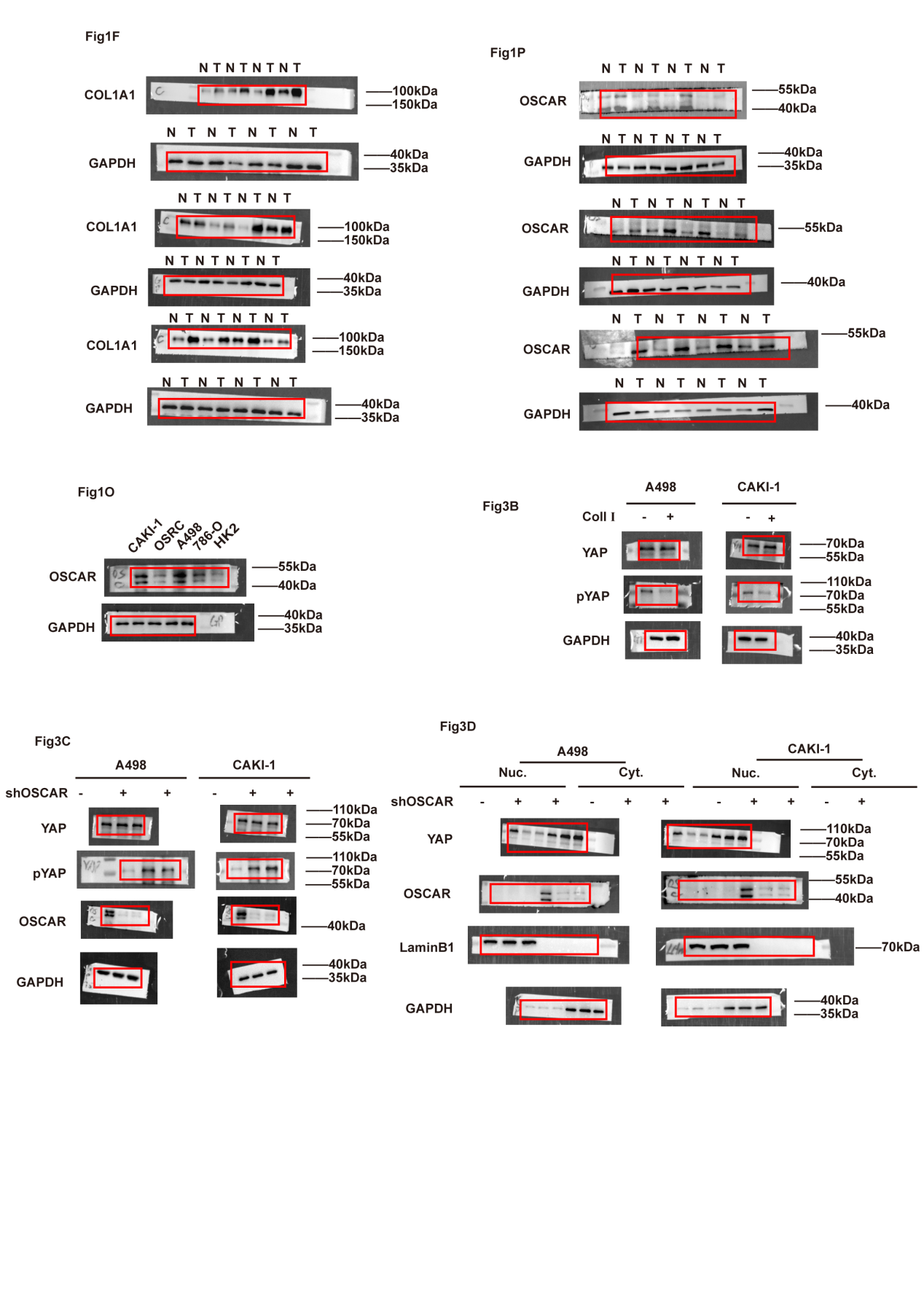

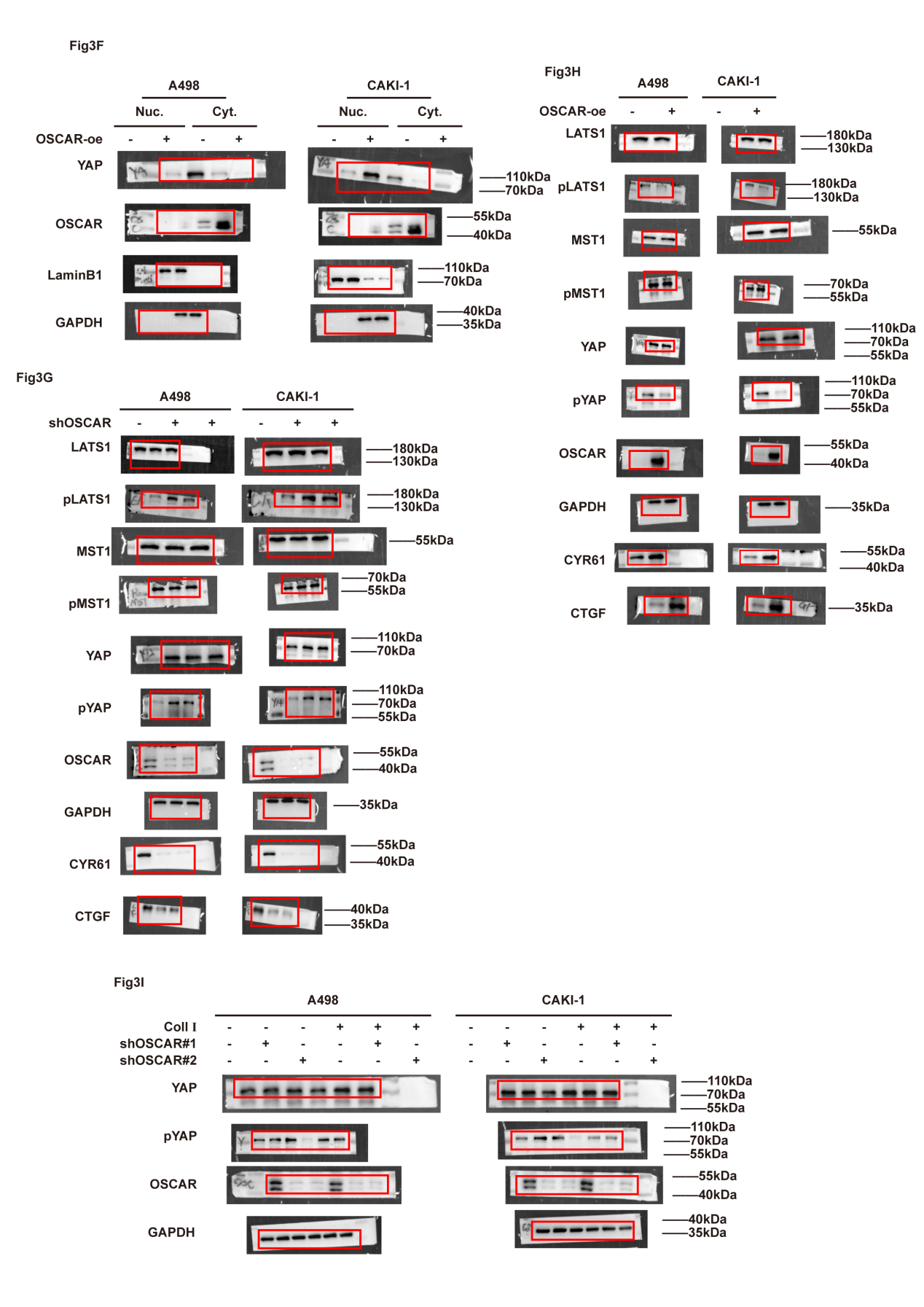

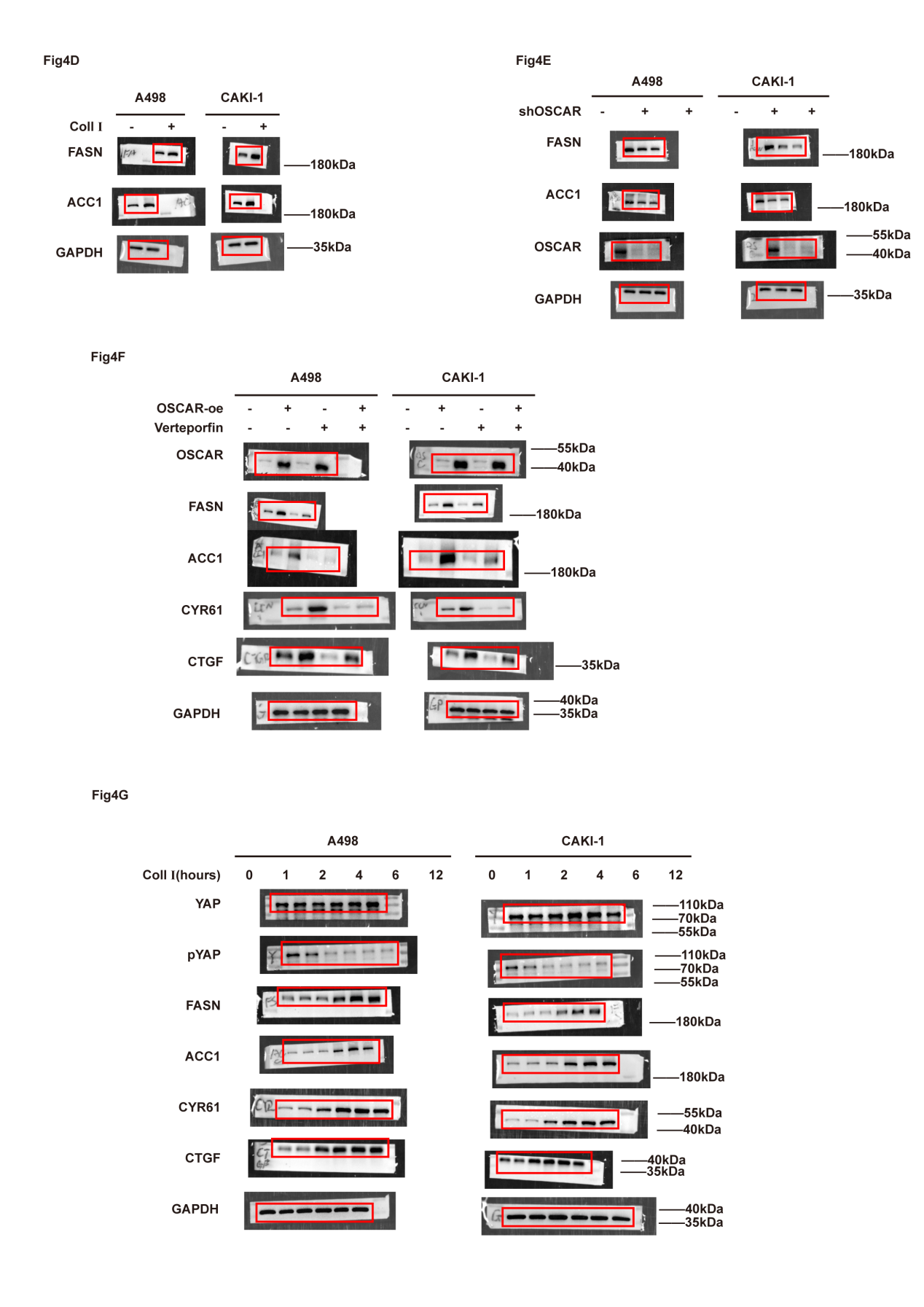

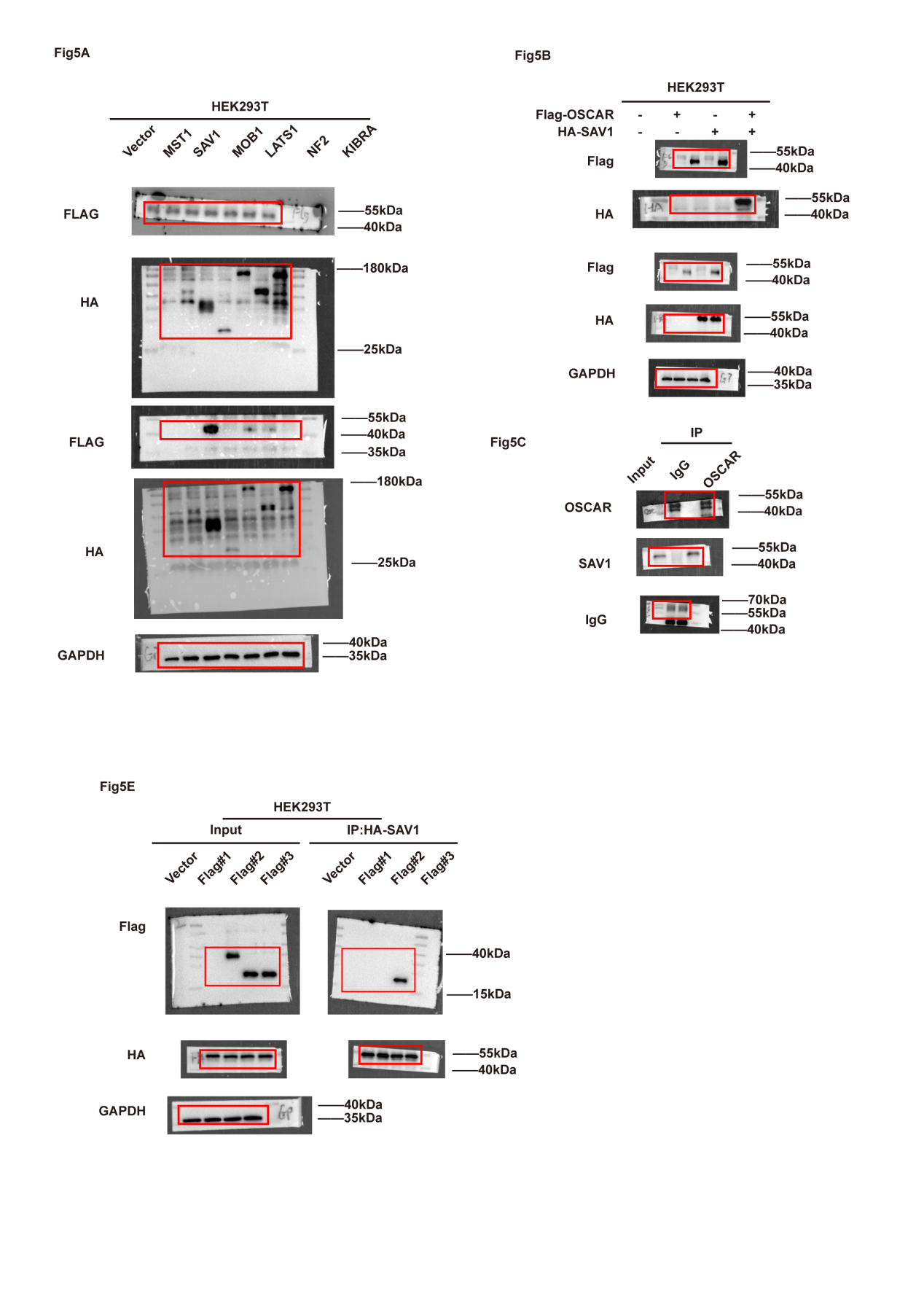

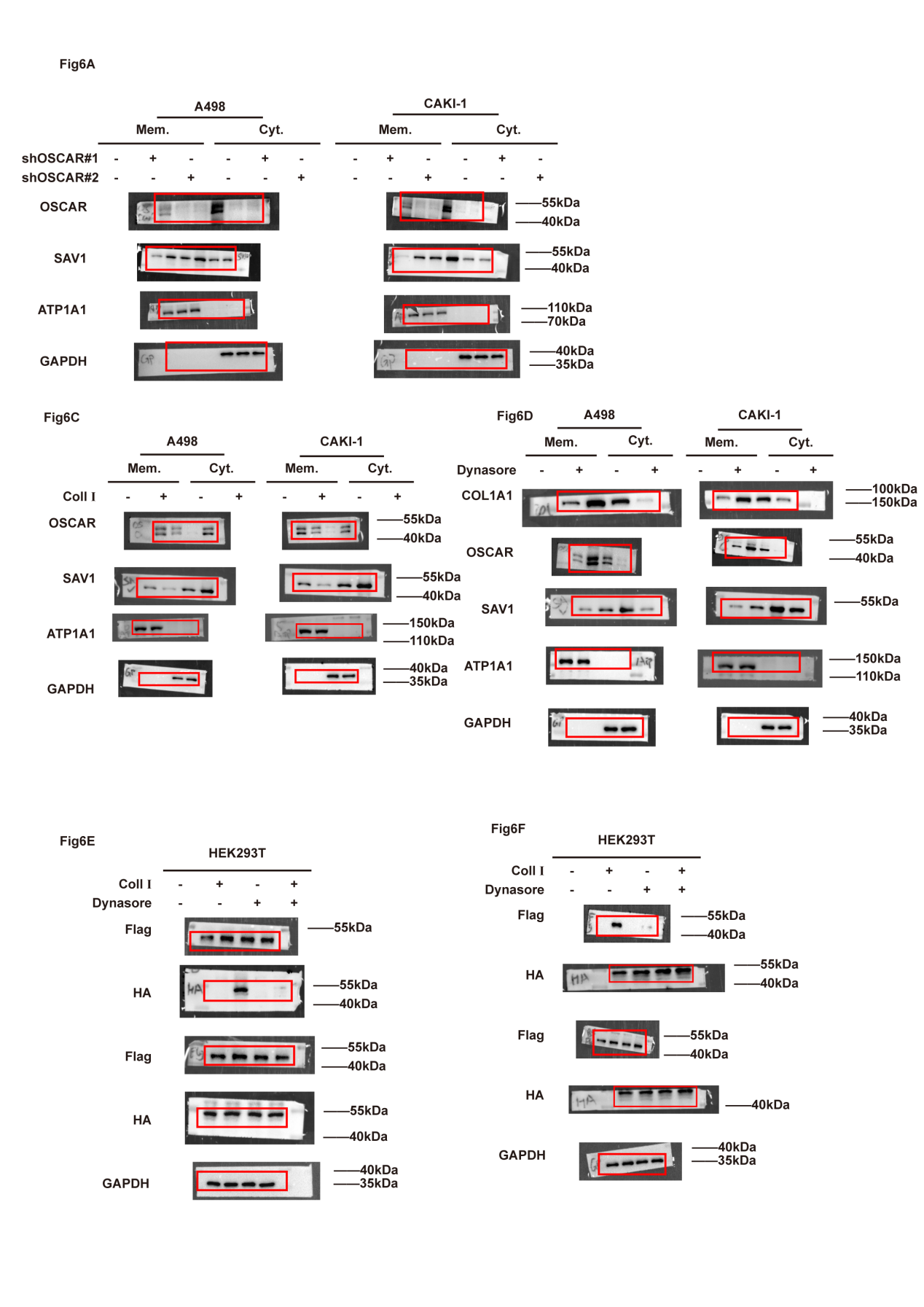

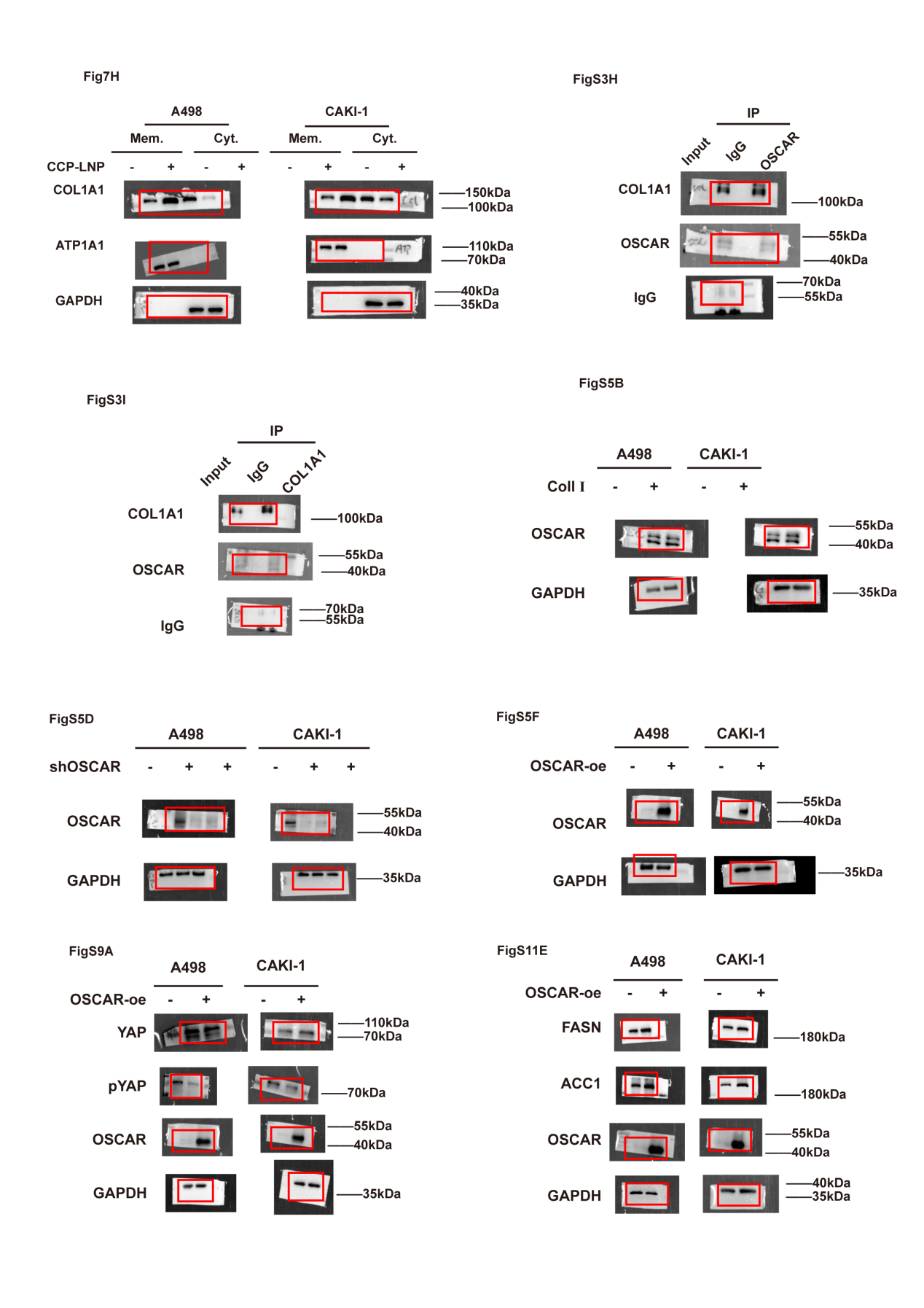

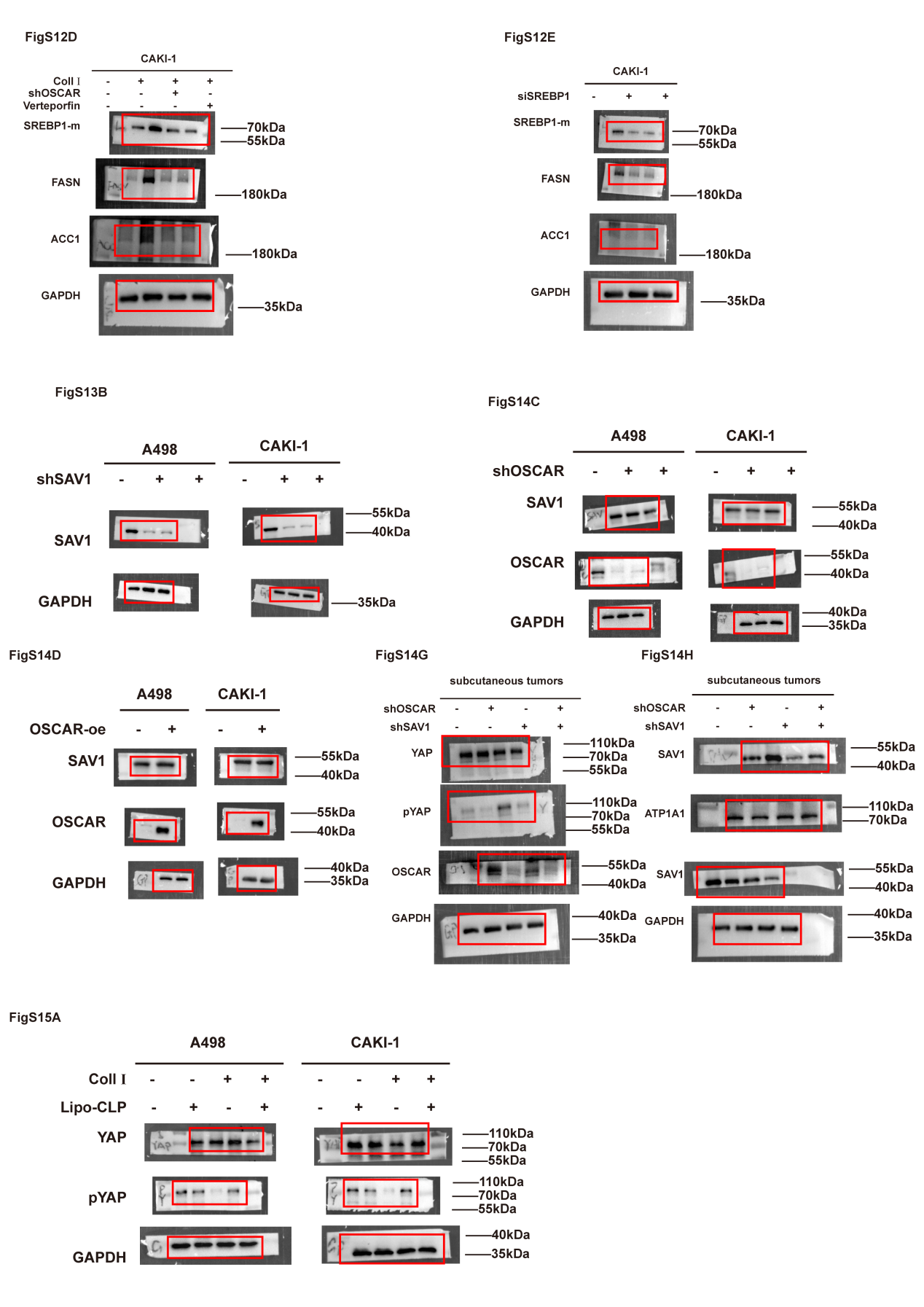

Supplement: Supplementary file 2 — Original uncropped images of all blots [file 41419_2026_8713_MOESM2_ESM.docx]
